# Impact of Biofilm Formation by Vaginal *Candida albicans* and *Candida glabrata* Isolates and Their Antifungal Resistance: A Comprehensive Study in Ecuadorian Women

**DOI:** 10.3390/jof11090620

**Published:** 2025-08-25

**Authors:** Ariana Cecibel Cedeño-Pinargote, Nicolás Renato Jara-Medina, Carlos C. Pineda-Cabrera, Darío F. Cueva, María P. Erazo-Garcia, Eduardo Tejera, António Machado

**Affiliations:** 1 Laboratorio de Bacteriología, Instituto de Microbiología, Colegio de Ciencias Biológicas y Ambientales COCIBA, Universidad San Francisco de Quito (USFQ), Calle Diego de Robles y Pampite, Quito 170901, Ecuador; accedenop@alumni.usfq.edu.ec (A.C.C.-P.); nrjara@alumni.usfq.edu.ec (N.R.J.-M.);; 2 Grupo de Bioquimioinformática, Facultad de Ingeniería y Ciencias Agropecuarias Aplicadas, Universidad de Las Américas (UDLA), De Los Colimes esq, Quito 170513, Ecuador; 3 Centro de Biotecnologia dos Açores (CBA), Departamento de Biologia, Faculdade de Ciências e Tecnologia, Universidade dos Açores, 9500-321 Ponta Delgada, Portugal

**Keywords:** biofilms, antimicrobial resistance, biofilm inhibition and eradication assays, antifungal treatments, *Candida albicans*, *Candida glabrata*

## Abstract

*Candida albicans* and *Candida glabrata* are key fungal pathogens linked to candidiasis, with rising concerns due to antifungal resistance and biofilm abilities. However, data from Latin America remains limited. This study assessed biofilm formation and antifungal susceptibility of vaginal *Candida* isolates from Ecuadorian women. Biofilm formation at 24 and 48 h was evaluated using biomass and CFU assays and the biofilm formation index. Antifungal resistance in planktonic cells and patient microbiota profiles were also analyzed. Biofilm assessment showed 57.14% of isolates were high biofilm formers, 33.33% intermediate, 4.76% low, and 4.76% non-formers. Planktonic susceptibility testing included fluconazole, voriconazole, posaconazole, caspofungin, anidulafungin, micafungin, flucytosine, and amphotericin B. Micafungin showed the lowest MBEC90 value among tested antifungals, with an average MIC of 0.15 µg/mL, MBIC90 of 1.26 µg/mL, and MBEC90 of 1.86 µg/mL. Fluconazole followed with MIC, MBIC90, and MBEC90 values of 4.19, 63.33, and 66.59 µg/mL. Flucytosine had the highest values (MIC = 11.36 µg/mL; MBIC90 = 244.71 µg/mL; MBEC90 = 245.33 µg/mL). Both micafungin and flucytosine produced similar reductions in viable biofilm cells (1.44 log CFU), while fluconazole induced a slightly lower reduction of 1.39 log CFU. Findings suggest echinocandins may be effective against biofilm-forming *Candida* in this Ecuadorian population subset.

## 1. Introduction

*Candidiasis*, an infection caused by *Candida* species, encompasses both superficial and systemic mycoses. While invasive candidiasis is a serious healthcare-associated fungal infection that can lead to bloodstream and deep-seated organ involvement, vulvovaginal candidiasis is a localized, mucosal infection and one of the most common fungal infections affecting women of reproductive age. *Candida* yeasts are commensal organisms commonly found in the human microbiota, including the vaginal mucosa, oral cavity, skin, gastrointestinal tract, nasal passages, and urethra [1]. These yeasts exhibit remarkable adaptability to diverse environments and are typically harmless; however, under certain conditions, they can become opportunistic pathogens [2,3,4].

In recent years, the global incidence of *Candida* infections has been rising [5,6,7,8]. To date, more than 30 *Candida* species have been identified as capable of causing human disease, and this number continues to grow [9,10,11]. Although *Candida albicans* remains the most frequently isolated species, non-*albicans Candida* (NAC) species such as *Candida glabrata* (also known as *Nakaseomyces glabrata* or *Nakaseomyces glabratus*) have shown increasing prevalence, especially in clinical settings [12,13,14].

One of the most important virulence factors of *Candida* species involves the formation of biofilms [15,16]. Biofilms are defined as structured microbial communities that are attached to a surface and encased in an extracellular matrix (ECM), forming a complex three-dimensional architecture on biotic and abiotic surfaces [17,18]. In recent decades, research in the field of biofilms has increased because it is known to be the normal growth state for most microorganisms [19,20,21].

The microorganisms of this type of community show a lower growth rate and a higher rate of resistance to treatments, behaving very differently from planktonic cells. *Candida* biofilms are different depending on the species, morphology, and metabolic activity. Due to these general characteristics, biofilms enhance the establishment of persistent infections in the human body [17,22]. Additionally, it is known that biofilms are inherently resistant to antifungals, especially amphotericin and fluconazole [23].

Candidiasis can reappear quickly, and poor medical care usually causes resistant candidiasis, so it is important to evaluate the antifungal activity of *Candida* species since there are still not many studies carried out in Ecuador [24,25,26,27]. The present study was carried out to isolate *Candida* species from vaginal samples, quantify the rate of biofilm formation, compare antifungal susceptibility profiles in planktonic cells, and characterize the efficacy of antifungals at different minimum biofilm inhibitory concentration (MBIC) and minimal biofilm eradication concentration (MBEC) values to prevent and eradicate *Candida*-associated biofilms.

## 2. Materials and Methods

### 2.1. Candida Isolates and Growth Conditions

Twenty-one vaginal *Candida* isolates were selected for the present study, which eighteen *C. albicans* isolates (designated as V118, V130, V134, V161, V196, V202, V218, V251, V252, V415, V448, V449, V450, V451, V527, V535, V540, and V580) and three *C. glabrata* isolates (designated as V197, V543, and V601) belong to the microbial collection of the Institute of Microbiology at Universidad San Francisco de Quito (IM-USFQ). All selected isolates included in this study were previously retrieved from a previous epidemiological study performed by our research group [27]. In that study, vaginal samples were collected to assess the presence of any type of infection or dysbiosis. Microbiological identification of the isolates followed a standardized protocol that included Gram staining procedures, wet mount smears, growth culture, biochemical properties, and molecular confirmation via PCR assays. This comprehensive methodology was consistently applied to all isolates to ensure accurate species identification and selection for subsequent analysis. As previously reported, *Candida* isolates were kept in Sabouraud dextrose broth (SBD; Dipco Cía. Ltda., Quito, Ecuador) with glycerol 20% at −80 °C, and 24 h before each assay, a new culture in Sabouraud dextrose agar (SBD; Dipco Cía. Ltda., Quito, Ecuador) was made and incubated at 37 °C for 24 h. Then, yeast cells were harvested and suspended in 5 mL of phosphate-buffered saline (PBS) to obtain a cellular density adjusted at $1\times 10^{7}$ colony-forming unit (CFU)/mL using a UV-Vis spectrometer (GENESYS™ 20 Thermo Scientific™, Waltham, MA, USA), as described before [16]. Finally, *C. albicans* of the American Type Culture Collection (ATCC) 10231 was included as a reference strain in both antifungal susceptibility and biofilm formation assays to serve as an internal control for experimental validation; it was the only standard strain available in our institutional laboratory at the time of this study.

### 2.2. Evaluation of the Antifungal Resistance of Candida Planktonic Cells

#### 2.2.1. CANDIDA EU Commercial Kit

Fungal susceptibility to the drugs fluconazole, voriconazole, posaconazole, caspofungin, anidulafungin, micafungin, flucytosine, and amphotericin B was performed using the SensiQuattro CANDIDA EU commercial kit (Liofilchem, Roseto degli Abruzzi, Italy). Briefly, 150 μL of *Candida* suspension whose turbidity was adjusted to 1 McFarland ($1\times 10^{8}$ CFU/mL) equivalent standards (ref. 80401) were transferred to each well of the 32-well panel containing the eight previously mentioned dried antimycotics in different concentrations. As indicated in the commercial kit, the 8 antimycotics were evaluated in the following concentration range: fluconazole, 1 to 8 µg/mL; voriconazole, 0.06 to 0.5 µg/mL; posaconazole and caspofungin, 0.03 to 0.25 µg/mL; anidulafungin, 0.015 to 0.12 µg/mL; micafungin, 0.03 to 0.25 µg/mL; flucytosine, 4 to 32 µg/mL; and finally, amphotericin, 0.5 to 2 µg/mL. The plate was covered with the lid provided in the kit and incubated at 34.5–35.5 °C for 24 ± 2 h. The results were interpreted according to the colour change and according to EUCAST clinical breakpoints [28]. The 21 *Candida* isolates and *C. albicans* ATCC 10231 in the planktonic growth form were classified as S (susceptible) or R (resistant) through the commercial kit SensiQuattro CANDIDA EU Panel for Determining the Susceptibility Testing of *Candida* spp. with antimycotics agents (Ref. 76033–79033; Liofilchem, Italy). Further evaluation was realized with a representative member of each antifungal family, more precisely fluconazole, micafungin, and flucytosine, through the microdilution method for minimum inhibitory concentration (MIC) assays and biofilm inhibition and eradication assays. The antimicrobial activity was evaluated by biomass quantification and CFU counting assays.

#### 2.2.2. Minimum Inhibitory Concentration (MIC)

The antifungal activity of the selected antifungals (fluconazole, micafungin, and flucytosine) against all *Candida* isolates was further evaluated using the microdilution testing for MIC assays described by Berkow and colleagues [29] as established by the Clinical Laboratory Standards Institute (CLSI) and European Committee on Antimicrobial Susceptibility Testing (EUCAST).

In the MIC assays, a serial dilution was carried out from 128 µg/mL for fluconazole, 512 µg/mL for flucytosine, and 4 µg/mL for micafungin powder in 190 µL of Sabouraud dextrose broth (SBD; Dipco Cía. Ltda., Quito, Ecuador) in 96-well plates, as previously mentioned [16,30]. The remaining 96-well plate well lines were then prepared with concentrations below 128 µg/mL, 512 µg/mL, and 4 µg/mL, respectively, in 190 µL SBD. Subsequently, 10 µL of each *Candida* species was added to increase the total volume to 200 µL with a final concentration of $1\times 10^{5}$ cells/mL of pathogen, and the 96-well plate was incubated for 24 h at 35 ± 2 °C [31]. A row of SBD plus inoculum was used as a *Candida* growth control (or positive control), and a row of wells with medium without inoculum was applied as a sterility control (negative control). Fluconazole was used as a reference control, with concentrations ranging from 1 to 128 µg/mL. The MIC values were measured by the unaided eye and a spectrophotometer, Biotek Instruments ELx808IU (Biotek, Winooski, VT, USA), at 630 nm (OD630) by comparing the results obtained with the negative control. Finally, the MIC was defined as the lowest concentration of antibiotic that inhibited the *Candida* growth after overnight incubation. All the assays were performed in triplicate on different days with independent repetitions.

### 2.3. Biofilm Formation

The previous inoculum of $1\times 10^{7}$ CFU/mL in PBS was centrifuged at 400 rpm for 10 min, and the pellet was resuspended in 5 mL of sterile SDB [16]. Two different approaches with 2 growth times (24 and 48 h) were used to evaluate biofilm ability through biomass formation by optical density assays, according to previous studies [16,32,33]. Briefly, into each well of the 96-well plate, 200 μL of prepared *Candida* medium suspension was transferred in quadruplicate wells for each *Candida* isolate/strain. The plates were incubated at 37 °C in static conditions for 24 h, and a second plate was set for 48 h. The wells were then washed with 200 μL PBS three times after incubation before the biofilm quantification (see next subsection). A negative control of medium without inoculum and a positive control of medium plus inoculum without the antifungal drug (when evaluating the antifungal resistance of different drugs) were also included in 6 wells of the 96-well plate. Then, the methodology was performed according to Kivanc & Er [1]. All assays were performed in triplicate on different days, and each assay had at least quadruplicate samples.

### 2.4. Quantification of Biofilm Formation

To screen the strain’s ability to form a biofilm, we used a biomass optical density (OD) assay with crystal violet (CV) staining and phosphate-buffered saline (PBS) suspension using a modified version of the method suggested by Gulati and colleagues [34]. Briefly, each optical density assay is described below.

#### 2.4.1. Phosphate-Buffered Saline Suspension

After 24 h of growth, the samples were washed three times with 200 μL of PBS. Then, the optical density values were measured at 570 nm and 630 nm for the 96-well plate using an ELISA Elx808 microplate spectrophotometer (Biotek, Winooski, VT, USA) [32]. All biofilm samples and negative controls were measured and further classified.

#### 2.4.2. Crystal Violet Staining

Additionally, another set of two 96-well plates was set at the same growth culture conditions previously mentioned for 24 and 48 h. Both were emptied and washed three times with 200 μL of PBS. Then, wells were fixed with 200 μL of 99% methanol for 15 min. After this period, the wells were emptied and left to dry at room temperature. Each well was then stained with 200 μL of 2% crystal violet solution for 5 min. After the wells were washed with 200 μL PBS, and dried. Finally, the wells were treated with 160 μL of 33% glacial acetic acid solution, and the 96-well plate was read in the ELISA Elx808 microplate spectrophotometer at 570 nm and 630 nm [1]. All biofilm samples and the negative controls were measured and further classified.

### 2.5. Biofilm Classification

The classification of the *Candida* isolate’s ability to form biofilm was realized through the biofilm formation index (BFI), as previously performed by Atiencia and colleagues [16]. Due to their variability in form, biofilms, biofilm-forming microorganisms are generally classified as non-forming (NBF), low (LBF), intermediate (IBF), and high (HBF), where BFI can be evaluated using different biofilm assay approaches [35]. Briefly, the biofilm-forming ability was assessed using crystal violet and PBS suspension assays. As previously described, *Candida* biofilms were obtained in 96-well plates at 37 °C for 24 h for PBS suspension, as well as 24 and 48 h for the crystal violet method. The biomass of biofilm samples was measured, and then each isolate was classified according to its biomass level.

For the PBS suspension assay, non-biofilm formers (NBFs) showed a biomass production less than or equal to the cut-off values ($OD_{c})$ ($OD\;\leq \;OD_{c})$. The cut-off values are obtained from the mean values of the negative controls and the standard deviation. Low biofilm formers (LBFs) evidenced a biomass production with greater cut-off values and lower than or equal to two times cut-off values ($OD_{c}<OD>2\times OD_{C})$, intermediate biofilm formers (IBFs) demonstrated a biomass production greater than two times cut-off values and lower than or equal to four times cut-off values ($2\times OD_{c}<OD>4\times OD_{C})$, and finally, high biofilm formers (HBFs) established a biomass production higher than four times the cut-off values ($OD\;>4\times OD_{C}),$ as previously described by Turan & Demirbilek [32].

For the crystal violet assay, *Candida* isolates were classified as non-biofilm formers (NBFs) when they had an OD value between 0≤OD570≤0.120, low biofilm formers (LBFs) when the biomass production was 0.121≤OD570≤0.240, intermediate biofilm formers (IBFs) when biofilm samples showed an OD in the range 0.241≤OD570≤0.500, and finally, high biofilm formers (HBFs) when their OD values are higher than OD570>0.500, as previously described by Kivanc & Er [1].

### 2.6. Antibiofilm Activity

#### 2.6.1. Biofilm Inhibition Assays

*Candida* samples were grown overnight, and an inoculum of $1\times 10^{7}$ CFU/mL in phosphate-buffered saline (PBS) was realized using a 1 McFarland standard turbidity ($1\times 10^{8}$ CFU/mL and then diluted 1:10). The previous inoculum was centrifuged at 400 rpm for 10 min, and the pellet was resuspended in 5 mL of sterile SDB [16]. Then, 190 µL of the prepared media suspension was added to 96-well plates and supplemented with 10 µL of concentrated antifungals (fluconazole, micafungin, and flucytosine) to fill 200 µL of the final volume in each well at 1 × to 32 × MIC values [16,31,36]. As previously mentioned, a row of SDB medium plus inoculum without the antifungal drug was used as a *Candida* growth control (or positive control), and a row of wells with medium without inoculum was applied as a sterility control (negative control).

#### 2.6.2. Biofilm Eradication Assays

*Candida* samples were grown overnight, and an inoculum of $1\times 10^{7}$ CFU/mL in phosphate-buffered saline (PBS) was realized using a 1 McFarland standard turbidity ($1\times 10^{8}$ CFU/mL and then diluted 1:10). The previous inoculum was centrifuged at 400 rpm for 10 min, and the pellet was resuspended in an equal volume of sterile SDB [16,30]. Then, 200 µL of the prepared media suspension was added to each well, as well as the negative control, and the plate was incubated for 24 h at 37 °C for biofilm formation. Subsequently, the SDB was gently removed from the wells, and a gentle wash with PBS was performed, adding the solution through 45 degrees in the well’s wall without touching the end of the well. The biofilms were then supplemented with 1 × to 32 × MIC values of concentrated antifungals (fluconazole, micafungin, and flucytosine) [16,36]. Plates were again incubated for 24 h at 37 °C, and then two washing steps were realized with PBS before biomass evaluation and CFU counting assays.

### 2.7. Biomass Evaluation

To measure the biomass growth and to further realize the CFU counting, the 96-well plates were washed two times with 200 µL of PBS. Then, the optical density values were measured at 630 nm for the 96-well plate using an ELISA Elx808 microplate spectrophotometer (Biotek, Winooski, VT, USA). The minimum biofilm inhibitory concentration (MBIC) and minimal biofilm eradication concentration (MBEC) at 90% were determined by biomass evaluation, as described in previous studies [37,38]. MBIC90 was defined as the minimum concentration of antifungal agents, leading to a 90% inhibition in biofilm formation compared with the control group. The biofilm reduction rate (%) was calculated according to the following formula:

$$Inhibition\;\left(\%\right)\;=\;\left(OD_{control}-OD_{sample}\right)/OD_{control}\times 100$$

MBEC90 was defined as the minimum concentration of antifungal agents that eradicated mature biofilm by 90% compared with the control group. The biofilm eradication rate (%) was calculated according to the following formula:

$$Eradication\;\left(\%\right)\;=\;\left(OD_{control}-\;OD_{sample}\right)/OD_{control}\times 100$$

Next, 100 μL of each well was used for CFU counting assays, as previously described [16].

### 2.8. Colony-Forming Unit Counting Assays

After biomass evaluation, MBIC and MBEC concentrations were selected for CFU evaluation and compared with *Candida* growth controls. Briefly, to enumerate sessile cells that could be cultured, a three-drop assay was used for CFU counting, and at least four individual PBS suspensions of each biofilm sample were used in a serial tenfold dilution, by adding 100 µL of sample (from each well) in 900 µL of sterile PBS. Each dilution was thoroughly vortexed, and pipette tips were changed before the next dilution or experimental step. Dilutions of 10^−3^, 10^−4^, 10^−5^, and 10^−6^ were plated in triplicate on sterile agar SDB and then incubated for 24 h at 37 °C, after which colonies were counted [16]. Therefore, several dilutions were available for each CFU assay being collected, and all data. For the statistical analysis, the dilution with a growth between 25 and 250 CFU was chosen according to Thomas et al. [39].

### 2.9. Statistical Analysis

All data in the present study were obtained from triplicate assays on different days, and each assay had at least quadruplicate samples. Data were analyzed using SPSS (version 28.0; SPSS Inc., New York, NY, USA) [40]. Hierarchical clustering was performed with the nearest neighbor method and using Euclidean distance to compare the resistance profile between the different classes of antifungals across all samples, as previously described in previous studies [26,41,42]. The dendrogram was constructed in SPSS, and the chemical drawing was performed using the online MolView website (https://molview.org). Additionally, we used the Chi-square test on the biofilm formation capacity data to determine the non-random association between the categorical variables under study, such as the obtained biofilm formation indexes using different approaches of biofilm assays. The non-parametric Kruskal–Wallis test was used to determine if there are statistically significant differences between the minimum inhibitory concentration (MIC) data of the eight antifungals and between the MIC, MBIC, and MBEC data of the antifungals (i.e., fluconazole, micafungin, and flucytosine). A clustering model was carried out using Ward’s Minimum Variance Clustering Method with Euclidean squared distance to perform hierarchical clustering obtained from the inhibition and eradication assays of the 22 strains with the three antifungals of interest previously mentioned using SPSS [26]. The Mann–Whitney U test was used to analyze MBIC and MBEC data, specifically to compare the outcomes between treatment and control groups of each antifungal [43].

## 3. Results

### 3.1. Diagnosis of Samples in the Study

All *Candida* isolates were retrieved from a previous epidemiological study performed by our research group [27], where vaginal samples were taken to identify the presence of any type of infection through microbiological and molecular tests. From an initial sample set of 414 volunteer women, vulvovaginal swabs and a medical survey were collected. Most women were between 21 and 30 years old, with 61.8% (256/414) in this age range, and all the samples with candidiasis. All women who presented with candidiasis were single (7/7), 71.4% (5/7) of them did not have a sexual partner at the time, and 28.6% (2/7) had a sexual partner. Additionally, 71.4% (5/7) used a contraceptive method, such as condoms or hormonal control. As a result, 21 *Candida* isolates from these women were collected and used in this study to evaluate their ability to form biofilm and their antifungal resistance (see Supplementary Table S1).

The 21 *Candida* isolates were obtained from different women, and their vaginal samples were diagnosed and classified into different types of microbiota (see Supplementary Table S2). The volunteered women were between 19 and 33 years old, but most were in their twenties. Of these women, 47.62% (10/21) indicated that they had a sexual partner, where 6/9 of the women had healthy microbiota, 3/7 of the women had candidiasis, and 1 woman had a mixed infection. Meanwhile, 33.33% (7/21) of the women used condoms (6/9 healthy microbiota; 1/3 with mixed infection) and 33.33% (7/21) of the women used hormonal birth control (2/9 healthy microbiota; 4/7 candidiasis; 1/3 mixed infection). Finally, 28.57% (6/21) of women did not use any contraceptive method (2/2 intermediate microbiota; 3/7 candidiasis; 1/3 mixed infection).

In our study set, 42.85% (9/21) of the vaginal samples were diagnosed with healthy microbiota, 9.52% (2/21) with intermediate microbiota, 33.33% (7/21) with vulvovaginal candidiasis (VVC), and 14.30% (3/21) with mixed infection (candidiasis plus another infection/dysbiosis such as aerobic vaginitis or bacterial vaginosis). The 30% (3/10) of VVC cases showed coinfections, with two combining VVC and aerobic vaginitis (AV) and one combining VVC and bacterial vaginosis (BV). Fourteen women indicated vaginal discharge at the time of the sample collection (6/9 healthy microbiota; 2/2 intermediate microbiota; 3/7 candidiasis; 3/3 mixed infection), with some reporting strong odor (2/9 healthy microbiota; 2/3 mixed infection) and discomfort (2/9 healthy microbiota; 1/7 candidiasis; 1/3 mixed infection).

**Figure 1:**
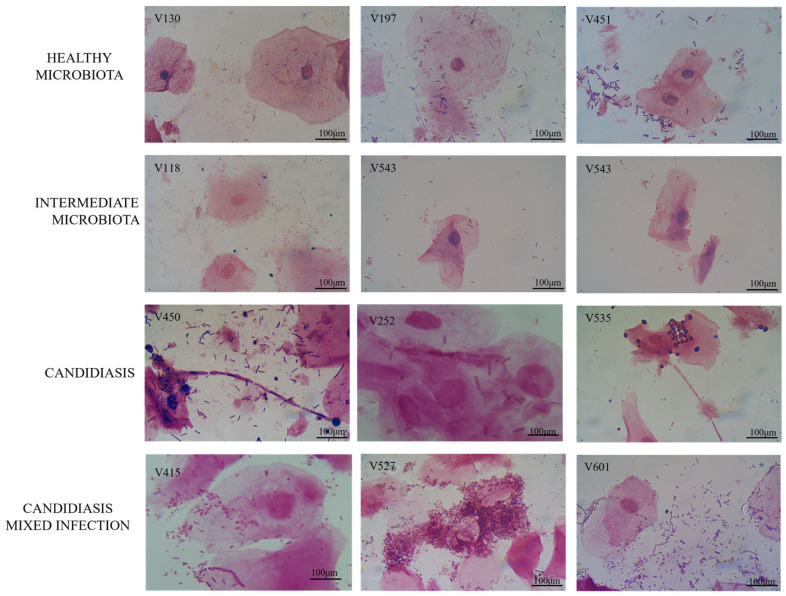
Representative images of each type of vaginal microbiota observed in the vaginal samples selected for the present study. These are original pictures from our research in this study.

Microscopically, the healthy microbiota samples showed well-formed epithelial cells protected by lactobacilli (V130, V197, and V451), while intermediate microbiota samples had reduced *Lactobacillus* species (V118 and V543). Candidiasis and VVC-related mixed infection samples demonstrated epithelial cell disruption and low or absent lactobacilli. Pure VVC samples showed fungal hypha growth, while VVC-related mixed infection samples had lower hyphal growth forms due to other opportunistic pathogens (see Figure 1).

### 3.2. Evaluation of the Antifungal Resistance of Candida Planktonic Cells

**Figure 2:**
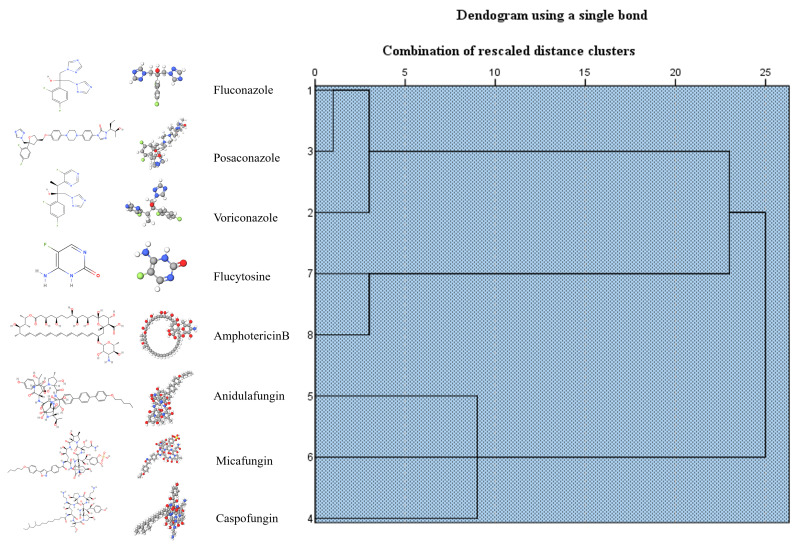
Cluster evaluation of the resistance profile obtained by different classes of antifungal agents against the *Candida* isolates in our study set.

The SensiQuattro CANDIDA EU Panel used to evaluate the susceptibility and resistance of eight antifungal agents against the *Candida* isolates, following the European Committee on Antimicrobial Susceptibility Testing (EUCAST) and Clinical Laboratory and Standards Institute (CLSI) guidelines for the breakout points [28,44] (see Supplementary Table S3). As shown in Figure 2, fluconazole and posaconazole revealed similar resistance profiles, while anidulafungin, micafungin, and caspofungin also demonstrated the same resistance profile, and finally, although flucytosine and amphotericin B are structurally not related, these antifungal agents were grouped in the same cluster, showing a higher rate of resistance among our *Candida* isolates.

**Figure 3:**
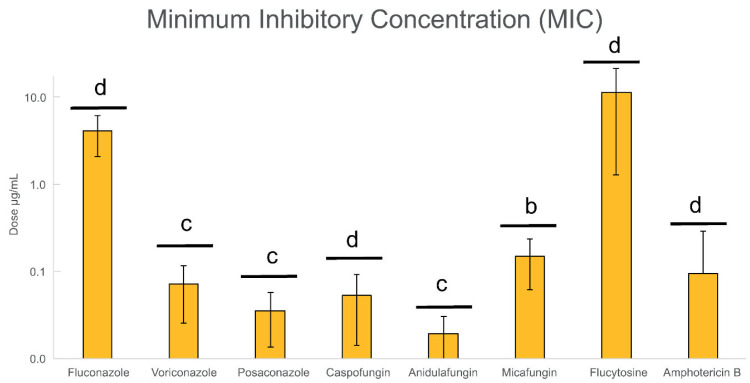
Determined values of the minimum inhibitory concentration (MIC) for the eight antifungal agents across all 21 *Candida* vaginal isolates. Each bar represents the average MIC value for each antifungal. The Kruskal–Wallis test was used to evaluate differences between MIC distributions for each antifungal agent, in order to determine whether there is a significant difference between all the variables. Statistical significance is indicated as follows: a *p* < 0.05; b *p* < 0.01; c *p* < 0.001; d *p* < 0.0001. The appearance of some error bars approaching zero (anidulafungin) reflects low variability and graphical rendering limitations in the plotting SPSS software (version 28.0).

Regarding antifungal concentrations on *Candida* planktonic cells, the number of susceptible and resistant isolates against antifungal agents of the same family showed almost the same resistance profile (see Supplementary Figure S1). Furthermore, when comparing to EUCAST and CLSI breakout points, triazoles had 9 resistant isolates (42.9%), echinocandins had 7–9 resistant isolates (33.3–42.9%), flucytosine had 19 resistant isolates (90.5%), and amphotericin B showed resistance in all isolates. The inhibition of planktonic growth by fluconazole, micafungin, and flucytosine was confirmed using the microdilution method, determining the minimal inhibitory concentration (MIC) values for the eight antifungal agents (see Figure 3).

Visible inhibition of growth of all *Candida* isolates was observed at various concentrations: 4 µg/mL fluconazole, 0.07 µg/mL voriconazole, 0.04 µg/mL posaconazole, 0.05 µg/mL caspofungin, 0.02 µg/mL anidulafungin, 0.15 µg/mL micafungin, 16 µg/mL flucytosine, and 0.10 µg/mL amphotericin B. Therefore, these concentrations were considered MIC values for further biofilm inhibition and eradication assays. To better analyze the selected variables, statistical comparison tests were conducted using the Kruskal–Wallis test between the different MIC values at different treatment concentrations. Anidulafungin (*p* < 0.001) was the most efficient antifungal within the study set of the eight antifungals, followed by micafungin (*p* < 0.01). On the other hand, the *Candida* isolates under study were mostly resistant to flucytosine (*p* < 0.0001) despite showing a MIC value. Considering that a high number of resistant isolates were found in our study set on *Candida* planktonic cells, further evaluation of antimicrobial activity through biofilm inhibition and eradication assays was considered to fully characterize the increment of antifungal resistance in this microbial form of growth on sensitive versus resistant *Candida* isolates.

### 3.3. Biofilm Formation

**Figure 4:**
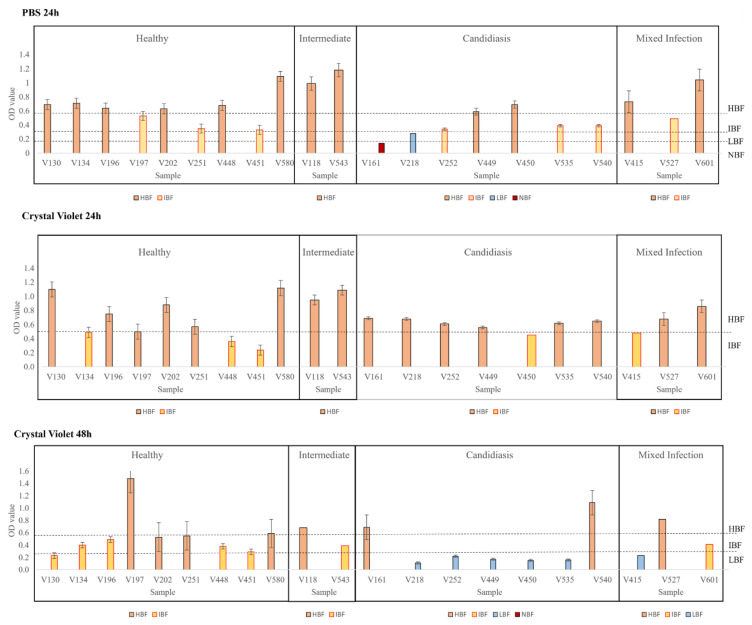
Illustration of the biofilm-forming ability of *Candida* isolates and their classification according to Turan & Demirbilek (2018) [32] and Kıvanç & Er (2020) [1].

Little is known about the ability of *Candida* isolates to form biofilms in studies involving vaginal samples in Latin America, especially in Ecuador. Although several methods allow analyzing the formation of biofilms, such as XTT, crystal violet (CV) staining, phosphate-buffered saline (PBS) suspension, and other assays [12], it is not a standard procedure in most microbiological laboratories. As shown in Supplementary Table S4, all *Candida* isolates were classified through the biofilm formation index (BFI) according to the Turan & Demirbilek (2018) and Kıvanç & Er (2020) approaches [1,32]. Regarding Turan & Demirbilek’s (2018) [32] first BFI approach, biofilm samples were grown for 24 h in PBS suspension and then measured (see Figure 4). Our *Candida* study set revealed that 57.14% (12/21) of the isolates were classified as HBFs (6/9 healthy microbiota; 2/2 intermediate microbiota; 2/7 candidiasis; 2/3 mixed infection), followed by 33.33% (7/21) of isolates as IBFs (3/9 healthy microbiota; 3/7 candidiasis; 1/3 mixed infection), and 4.76% (1/21) of the isolates were classified as LBFs (1/7 candidiasis) and NBFs (1/7 candidiasis). Meanwhile, concerning Kıvanç & Er’s (2020) [1] second BFI approach at 24 h, all *Candida* isolates were classified into merely two categories (see Figure 4), more precisely, 76.19% (16/21) as HBFs (6/9 healthy microbiota; 2/2 intermediate microbiota; 6/7 candidiasis; 2/3 mixed infection) and 23.81% (5/21) as IBFs (3/9 healthy microbiota; 1/7 candidiasis; 1/3 mixed infection). When further evaluating at 48 h of biofilm growth culture (see Figure 4), the approach demonstrated a more diverse categorization showing 38.1% (8/21) of isolates as HBFs (4/9 healthy microbiota; 1/2 intermediate microbiota; 2/7 candidiasis; 1/3 mixed infection), followed by 33.33% (7/21) of isolates as IBFs (5/9 healthy microbiota; 1/2 intermediate microbiota; 1/3 mixed infection), and 28.57% (6/21) of isolates as LBFs (5/7 candidiasis; 1/3 mixed infection).

When comparing the two BFI approaches, both methodologies showed a dominance of high biofilm-forming *Candida* isolates in our study set. However, there is a huge ambiguity in the results between 24 and 48 h by CV staining, contrasting also from the results obtained by the PBS suspension approach. Therefore, Chi-square tests were performed between BFI approaches using the PBS suspension approach as a reference because of our previous study [16]. A *p*-value of 0.481 was obtained between the PBS suspension at 24 h and the CV staining at 48 h, demonstrating a coincidence of 57.14% between the results and thus a lack of relationship between these two approaches. However, when comparing the PBS suspension and CV staining both at 24 h, a coincidence of 90.48% was obtained between the results of the PBS suspension and CV staining at 24 h, justifying the application of the 24 h PBS method in the evaluation of the antifungal resistance on *Candida* biofilm cells.

### 3.4. Biofilm Inhibition and Eradication Assays

**Figure 5:**
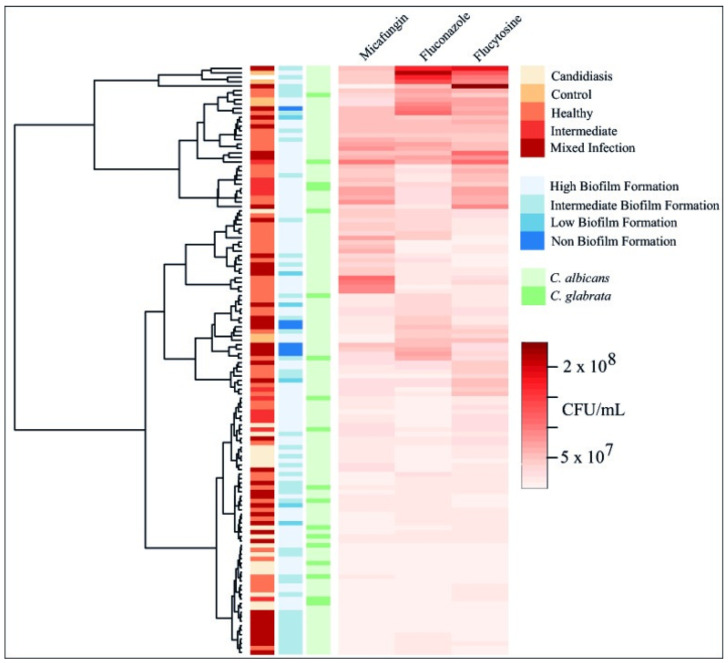
Heatmap with hierarchical clustering (Ward method) of biofilm inhibition profiles of 21 vaginal *Candida* isolates and the reference strain *C. albicans* ATCC 10231 following exposure to micafungin, fluconazole, and flucytosine. Columns display patient microbiota classification, biofilm-forming capacity, *Candida* species, and log CFU reduction by antifungal agent. Color intensity represents inhibition strength, with darker shades indicating greater biofilm inhibition.

Both biofilm inhibition and eradication assays were realized in the 21 *Candida* isolates plus *C. albicans* ATCC 10231 using the 24 h PBS method and evaluating three representative antifungal agents, more precisely fluconazole, micafungin, and flucytosine. As shown in Figure 5, Ward’s hierarchical cluster methodology was applied to evaluate the overall results of biofilm inhibition assays with three distinct categorizations of the samples, i.e., vaginal microbiota classification, biofilm formation, and *Candida* species.

Surprisingly, the Ward’s hierarchical cluster analysis showed no clear pattern by vaginal microbiota classification, biofilm formation, or *Candida* species when treated with the three antifungals. The effectiveness of the three antifungals did not correlate with the classification of the microbiota type, as expected, nor the intrinsic ability to form biofilm, nor even the *Candida* species. The three antifungals managed to inhibit the development of biofilm biomass from values of MIC 1× to 32× for 24 h at 37 °C, ranging between 0.3% and 99% of biofilm inhibition (see Supplementary Table S3), respectively. Surprisingly, flucytosine showed statistical differences in biofilm inhibition in all *Candida* isolates between 25 and 98% of samples (*p* < 0.0001), despite the greater resistance obtained by the group set in the planktonic growth evaluation. Moreover, the biofilm inhibition ranges obtained by micafungin, fluconazole, and flucytosine were 0.3–99%, 7–99%, and 25–98% (see Supplementary Table S5), respectively. Next, the MBIC and MBEC values were also calculated to compare the effectiveness between planktonic and biofilm treatments, as well as the differences between MBEC and MBIC values, as previously described in similar studies [32,45]. As shown in Table 1, MBIC90 values ranged from 4 to 128 µg/mL for fluconazole, 0.25 to 4 µg/mL for micafungin, and 64 to 512 µg/mL for flucytosine, demonstrating an increased antifungal resistance in biofilm inhibition treatment with fluconazole, micafungin, and flucytosine when compared with MIC values from planktonic evaluation. More precisely, MBIC values showed an average incremental resistance of fluconazole, micafungin, and flucytosine by a factor of 16. However, several exceptions of increased resistance were observed within our group set, where the isolates (V527 and V218) evidenced an increased factor of 64 for fluconazole and flucytosine, respectively, and another isolate (V415) showed an increased factor of 33 to micafungin. Meanwhile, some isolates demonstrated reduced resistance to fluconazole (5/22), micafungin (3/22), and flucytosine (1/22) with a resistance factor of 4 for all three antifungals. Finally, some cases showed no MBIC values, more precisely 6, 10, and 14 *Candida* isolates for fluconazole, micafungin, and flucytosine, respectively. It is also worth noting that the evaluation of biofilm biomass assays at optical densities of 570 nm and 630 nm revealed no statistically significant difference between the measurements (Pearson correlation = 0.971; see Supplementary Figure S2).

**Figure 6:**
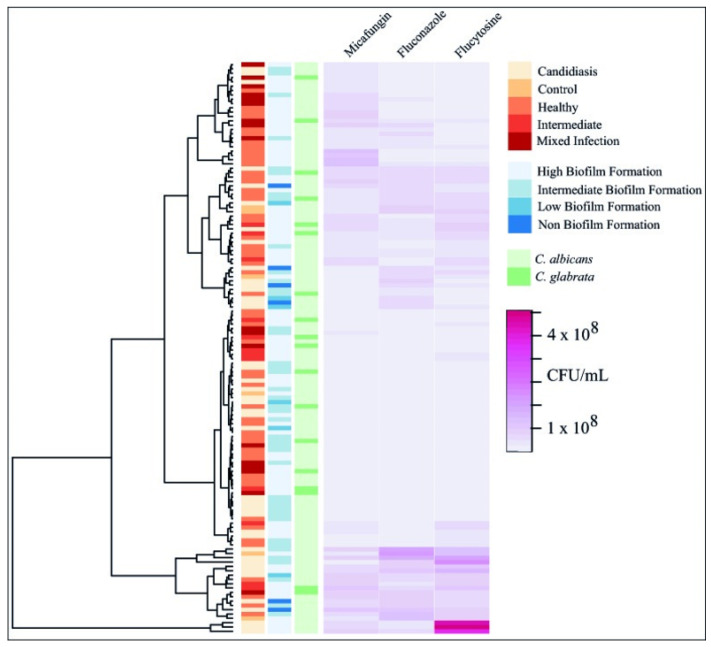
Heatmap with hierarchical clustering (Ward method) of biofilm eradication profiles of 21 vaginal *Candida* isolates and the reference strain *C. albicans* ATCC 10231 after treatment with micafungin, fluconazole, and flucytosine. Columns include microbiota classification, biofilm-forming ability, *Candida* species, and log CFU reduction. Color gradients indicate the extent of eradication, with darker hues denoting higher antifungal efficacy.

Further evaluation was realized on the number of sessile cells that could be cultured (viable cells) after MBIC treatment and control samples. All *Candida* isolates revealed a significant reduction in the CFU log (see Supplementary Figure S3). This reduction was consistent with approximately 1.43 CFU logs for the three antifungal agents when compared with control samples (*p* < 0.0001). In the control group, all strains exhibited growth in the medium of approximately CFU/mL, whereas the growth of treated samples showed a reduction to roughly CFU/mL. Treated samples with fluconazole and flucytosine evidenced a reduction of 1 to 2 CFU logs across the 22 *Candida* strains. Micafungin showed a reduction of 1 to 3 CFU logs across the group set, evidencing the greatest reduction. Regarding MBEC values, all *Candida* isolates demonstrated higher antifungal resistance during the treatment when compared with MBIC values, showing an average incremental resistance to fluconazole (11/22), micafungin (7/22), and flucytosine (9/22) by a factor of 1. Once again, several exceptions were also observed within our group set, where 2/22 exhibited increased MBEC/MBIC resistance by factors of 2 and 8 for fluconazole and by factors of 4 and 8 for flucytosine. Whereas 3/22 showed increased resistance to micafungin by factors of 4, 8, and 33. Likewise, certain strains displayed reduced MBEC/MBIC resistance to fluconazole (4/22) by factors of 0.5 and 0.25, micafungin (7/22) by factors of 0.5, 0.25, and 0.13, and flucytosine (5/22) by a factor of 0.5. Finally, MBEC values could not be determined in 4/22 isolates for both fluconazole and micafungin, as well as 5/22 isolates for flucytosine. When evaluating patterns, Ward’s hierarchical cluster analysis on biofilm eradication assays evidenced the same trend of results previously observed through biofilm inhibition assays (see Figure 6), showing clusters formed independently of the vaginal microbiota type, *Candida* species, or the ability to form biofilm. As shown in Table 1, MBEC90 values were generally higher, reaching up to 128 µg/mL for fluconazole, 4 µg/mL for micafungin, and 512 µg/mL for flucytosine. Resistant isolates were also identified in samples from healthy women, and several isolates showed no inhibition or eradication under the tested conditions. Further evaluation of biofilm eradication confirmed that higher antifungal concentrations were required compared with inhibition assays. MBEC90 values for fluconazole ranged from 4 to 128 µg/mL, for micafungin from 0.5 to 4 µg/mL, and for flucytosine from 32 to 512 µg/mL. Several isolates did not show complete biofilm eradication at the highest concentrations tested, which is reflected by the hyphens (-) in Table 1. Overall, the biofilm eradication rates for micafungin, fluconazole, and flucytosine were 10–99%, 1.95–99%, and 3.85–99% (see Supplementary Table S6), respectively.

The 22 vaginal *Candida* isolates exhibited comparable reductions in CFU counts following treatment with the three antifungal agents, averaging a decrease of approximately 1.43 log CFU compared with untreated controls (*p* < 0.0001; see Supplementary Figure S4). In the control group, *Candida* biofilms demonstrated robust growth, reaching approximately 2.35 × 10^8^ CFU/mL, whereas treated samples showed markedly reduced viability, with counts ranging from 1.13 × 10^5^ to 4.76 × 10^6^ CFU/mL.

**Figure 7:**
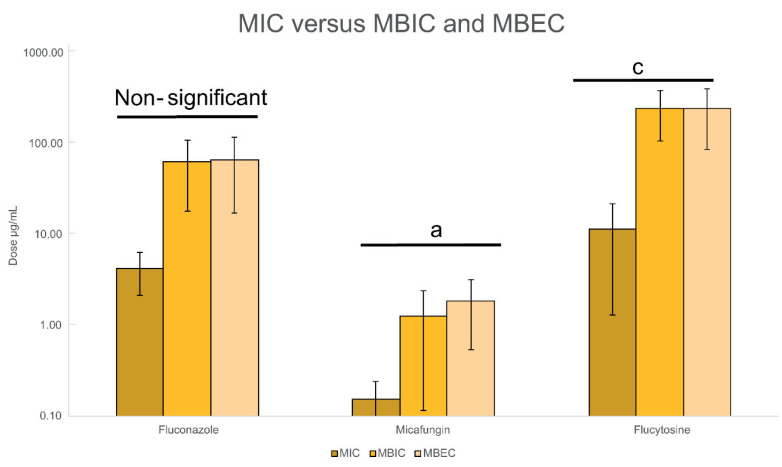
Mean MIC, MBIC, and MBEC values (μg/mL) for fluconazole, micafungin, and flucytosine. Statistically significant differences between the parameters were analyzed by the Kruskal–Wallis test (a *p* < 0.05; b *p* < 0.01; c *p* < 0.001; d *p* <0.0001).

As shown in Figure 7, micafungin demonstrated the lowest MBEC90 values among the tested antifungals, with average MICs of 0.15 µg/mL, MBIC90 of 1.26 µg/mL, and MBEC90 of 1.86 µg/mL. Fluconazole followed with MIC, MBIC90, and MBEC90 values of 4.19, 63.33, and 66.59 µg/mL, respectively. Flucytosine exhibited the highest values (MIC = 11.36 µg/mL; MBIC90 = 244.71 µg/mL; MBEC90 = 245.33 µg/mL). Both micafungin and flucytosine produced similar average reductions in viable biofilm cells (1.44 log CFU), while fluconazole induced a slightly lower reduction of 1.39 log CFU.

Statistically significant differences were observed in the MIC, MBIC90, and MBEC90 values among the 22 isolates for micafungin (*p* < 0.05) and flucytosine (*p* < 0.001), whereas fluconazole showed no statistically significant variation. Overall, these findings reinforce the elevated antifungal resistance associated with the biofilm phenotype and underscore the superior efficacy of micafungin for both biofilm inhibition and eradication, with observed inhibition ranging from 0.3% to 99% (*p* < 0.0001) and eradication from 10% to 99% (*p* < 0.001).

## 4. Discussion

*Candida* species constitute yeasts that can act as an opportunistic pathogen once there is a disruption of the host’s immune defense [7,46]. The increase in the misuse of antifungals and the number of immunocompromised patients or patients receiving invasive treatments has caused candidiasis to become an alarming opportunistic disease [19,47,48]. The present study evidenced the presence of *Candida* species in every type of vaginal microbiota. As expected, the most predominant vaginal *Candida* species in our study set was *C. albicans* (85.71%), followed by *C. glabrata* (14.29%). Our findings are similar to data reported by Marak et al. [49] and Tortelli et al. [50], which stated prevalences of 45.5–90% of *C. albicans* and 3.33–10% of *C. glabrata* in their study set. In agreement, this study showed *C. albicans* as the dominant *Candida* species in the vaginal microbiome. However, the prevalence of non-*albicans Candida* (NAC) species in the vaginal microbiome varies among women, ranging from ~10 to 30% [50]. Moreover, vaginal *Candida* colonization could lead to the development of candidiasis in women, as an opportunistic infection characterized by an overgrowth of *Candida* species and the diminution of the probiotic lactobacilli [51,52], leading to the destruction of vaginal epithelial cells and thus an aggressive immune response in the host [7,46].

Vulvovaginal candidiasis (VVC) frequently occurs in different age groups, as observed in the present study, when compared with VVC mixed with vaginal dysbiosis, more precisely, aerobic vaginitis (AV) in an age group between 20 and 23 years old, and a single case of bacterial vaginosis (BV) at age 21. According to the surveys carried out by Nasir et al., the presence of VVC was found in the age group between 21 and 40 years old, agreeing with our study, which reported VVC in women between 21 and 30 years old [53]. Taking into consideration single women as the group with the greatest susceptibility for VVC development, our results agree with Sajjan et al. [54], who reported that the greatest recovery of *Candida* isolates came from vaginal swabs of single women. Meanwhile, the majority of BV cases were obtained from single women who did not use a safe contraceptive method, where three of them had a sexual partner and manifested symptoms (discomfort), suggesting that behavior factors have a direct effect on the risk of acquiring candidiasis, as postulated by many authors [55,56,57]. It is estimated that condom use at the time of sexual intercourse is an important factor in terms of the health of the vaginal microbiota [27,58]. In our group set, six of the nine women with a healthy microbiota used condoms, and no symptoms were reported, contrasting with women with the presence of mixed infection or dysbiosis who, despite the use of condoms, already reported symptoms of both infections and previous clinical treatments. Therefore, the success of clinical treatments is vital for these cases, and it is important to monitor the antifungal resistance among *Candida* isolates of a specific population, such as Ecuadorian women, allowing optimal treatments with higher efficiency and lower rates of reinfection among patients.

In our study, all 21 vaginal *Candida* isolates exhibited resistance to amphotericin B across the tested concentrations. Amphotericin B resistance has also been reported in previous studies evaluating vaginal *Candida albicans* and *C. glabrata* isolates, indicating reduced susceptibility among certain subsets of clinical vaginal isolates [59,60,61]. Sarpong et al. [59] reported that 46.1% of vaginal *C. albicans* isolates from symptomatic women in Ghana were resistant to amphotericin B, despite the overall classification of the drug as largely effective. Danby et al. [60] demonstrated that under acidic vaginal conditions (pH 4.0), the MIC90 of amphotericin B increased significantly for both *C. glabrata* and fluconazole-resistant *C. albicans* strains, suggesting diminished efficacy in the vaginal environment. Similarly, Khan et al. [61] documented the progressive development of amphotericin B resistance in sequential vaginal *C. glabrata* isolates during prolonged antifungal therapy, with MICs exceeding 32 µg/mL in a single-strain case. These findings collectively support our observation that amphotericin B resistance may be more common in vaginal *Candida* isolates than previously recognized, particularly in specific clinical contexts. However, our results should be interpreted as representative of this particular study population and not as evidence of a nationwide trend in Ecuador. Moreover, 42.9% of *Candida* isolates were resistant to triazoles in our study, more specifically, fluconazole, voriconazole, and posaconazole. Although azole antifungals have long provided effective treatment [62,63], current studies showed the intrinsic resistance to azoles in various *Candida* species [13,64,65]. Even though the majority of our group set was *C. albicans*, the three *C. glabrata* isolates also showed resistance against triazoles, which agrees with the findings of Fothergill et al. [66]. In fact, these authors already reported an increase in the resistance rate previously established by the Clinical and Laboratory Standards Institute (CLSI), evidencing a resistance increase from 6.1% to 18.4% for voriconazole. The echinocandins family showed a resistance range between 33.3% and 42.9% of *Candida* isolates within our study set, demonstrating the largest number of resistant strains with caspofungin in its highest concentration against planktonic cells. Studies carried out in Europe showed that resistance to echinocandins still seems insignificant, with a resistance rate between 0.5% and 10% [67,68]. However, our results demonstrated an alarming increase in the resistance to caspofungin since almost half of the *Candida* isolates were not inhibited. In addition, Galia et al. [69] evidenced lower resistance rates in their group set of 30 women, more precisely, 1.4% of resistant strains for caspofungin, 2.9% for anidulafungin, and 1.3% for micafungin, thus contrasting with our results. The present study revealed a higher percentage of resistance to caspofungin among Ecuadorian women. Finally, little has been studied about the flucytosine resistance rate in microbiological studies. However, studies carried out by Charlier et al. [70] and Kim et al. [71] discovered that *Candida* isolates from patients became resistant to flucytosine after the treatment was finished, during the period of 6 days to 6 months. Moreover, Kim et al. [71] also observed an increase in flucytosine resistance in clinical settings during 2013–2023, whereas *C. albicans* isolates were susceptible to caspofungin and micafungin, which agrees with the results of the present study. However, it is necessary to carry out more studies regarding this antifungal to make better comparisons with our preliminary analysis.

In this study, eight antifungal agents from four pharmacological classes (azoles, echinocandins, pyrimidine analogs, and polyenes) were tested against vaginal *C. albicans* and *C. glabrata* isolates. The azole compounds fluconazole, voriconazole, and posaconazole inhibit the fungal cytochrome P450 enzyme lanosterol 14α-demethylase (encoded by ERG11), disrupting ergosterol biosynthesis and leading to altered membrane structure and function [72]. Resistance to azoles has been widely documented and is often mediated by *ERG11* mutations, overexpression of efflux pumps such as CDR1, CDR2, and MDR1, or by chromosomal alterations that enhance drug efflux or reduce target binding affinity [73,74]. Meanwhile, echinocandins (anidulafungin, micafungin, and caspofungin) act by non-competitively inhibiting the enzyme 1,3-β-D-glucan synthase (FKS1/FKS2), thereby blocking the synthesis of an essential component of the fungal cell wall [75]. Resistance to this class has been primarily associated with mutations in conserved hotspot regions of the *FKS* genes, which reduce drug binding and are particularly problematic in biofilm-forming *Candida* strains [76,77]. Furthermore, flucytosine, as a fluorinated pyrimidine analog, is converted intracellularly into 5-fluorouracil (5-FU), which inhibits thymidylate synthase and incorporates into RNA, disrupting DNA and protein synthesis [78]. Resistance can arise through mutations in *FCY2* (cytosine permease), *FCY1* (cytosine deaminase), or *FUR1* (uracil phosphoribosyltransferase), which impair drug uptake or activation [79]. Finally, amphotericin B is a polyene antifungal that binds to ergosterol in the fungal cell membrane, forming pores that lead to leakage of ions and cell death [80]. Resistance, although rare, has been described in *Candida* isolates and is often related to reduced ergosterol content or altered sterol composition because of mutations in *ERG* genes, which reduce polyene binding affinity [73,81,82]. These known resistance mechanisms may help explain the reduced antifungal susceptibility observed in our vaginal *Candida* isolates, especially in the context of prior antifungal exposure, host factors, and biofilm-related tolerance. Future studies are needed to fully characterize the resistance mechanisms observed in this specific study set.

The ability to establish biofilms is essential for the pathogenicity and virulence of *Candida* species during vulvovaginal candidiasis [12,47]. As expected, the different vaginal isolates and *Candida* species demonstrated their ability to form biofilms. The present study proved that biofilm production was 100% among all *Candida* isolates using the 24 h PBS suspension assays, although the remaining applied methodologies (24 h CV and 48 h CV staining assays) showed a lower rate. In recent studies that applied similar methodologies [16,32], it is shown that the application of this biomass assay (in particular, 24 h PBS suspension) in the evaluation of biofilm formation is more accurate in the data analysis and evidences a formation rate of 90–80% among their clinical isolates. When comparing *Candida* species, both *C. albicans* and *C. glabrata* demonstrated a good ability to form biofilms, since the vaginal isolates studied evidenced a greater high–intermediate biofilm-forming classification despite a few exceptions when obtaining low–non-biofilm-forming strains by other methodologies. Once the planktonic activity tests of the previously explained antifungals were carried out, an additional evaluation was realized through biofilm inhibition and eradication assays using three antifungals through biomass and colony-forming unit (CFU) counting assays. Regarding biomass evaluation, as explained previously, 90 percent inhibition and eradication were contemplated. Fluconazole achieved 90% biofilm inhibition at different concentrations in the 22 samples under study, of which the majority showed significant inhibition at 64 µg/mL (16× MIC value). Most *Candida* isolates demonstrated a significant reduction at 128 µg/mL (32× MIC value) in the eradication assays. Several studies reported higher biofilm inhibitions of 90% (MBIC90) with fluconazole, ranging from 640 to 2048 µg/mL, against *C. albicans* from different sources, such as samples obtained from vaginitis, candiduria, mouth, and blood cultures [32,83]. Concerning biofilm eradication assays, the results obtained in this study are more similar to those obtained by Romera et al. [84], where 90% eradication was obtained at 256 µg/mL in *C. albicans* ATCC 10231. However, in the same study, the MBEC90 value was 128 µg/mL against *C. parapsilosis* ATCC 22019, obtaining a similar eradication percentage [84]. As previously reported in the literature, the echinocandins had strong activity against *Candida* spp. biofilms [85,86,87]. In the present study, micafungin showed 90% biomass inhibition in most samples at 0.5 µg/mL (4× MIC value), and biomass eradication at 2 µg/mL (16× MIC value). This contrasts with results obtained by Fernandes et al. [88], which reported higher concentrations for *C. albicans*, more precisely MBIC90 values from 16 to 128 µg/mL; meanwhile, other *Candida* species, such as *C. tropicalis* and *C. parapsilosis,* obtained MBIC90 values ranging from 2 to 64 µg/mL. Regarding eradication, different *Candida* species treated with micafungin demonstrated MBEC50 values of 3–16 µg/mL [88]. The vaginal tract samples reported by Rodrigues et al. [89] obtained 50–80% of biomass eradication at 5.5–6 µg/mL for *C. glabrata* and 50% of biomass eradication at 3.5 µg/mL for *C. albicans*. Furthermore, eradication assays have been carried out on other opportunistic fungal pathogens such as *Lomentospora prolificans*, which evidenced 50% biomass eradication with micafungin at 1 µg/mL [90]. Meanwhile, in the present study, flucytosine showed 90% biomass inhibition at concentrations of 256 µg/mL (16× MIC value). Further evaluation demonstrated that flucytosine at 128 and 256 µg/mL (8× and 16× MIC value) was able to eradicate 90% of *C. albicans* and *C. glabrata* biofilms. These results contrast with the previous literature that reported 50% biofilm eradication for *Candida glabrata* at 32 and 64 µg/mL of flucytosine [91]. Due to the high resistance and lack of studies with flucytosine, little is still known about its effectiveness against *Candida* species in biofilms. There are not many literature studies related to the cellular viability results (CFU/mL) in biofilm inhibition and eradication with *Candida* species using the same type of antifungals, and, therefore, this is one of the novelties of the present study. Future studies should also evaluate the cell viability of *Candida* biofilms after treatment. The present study demonstrated significant and different biofilm reduction in the CFU log counting among different antifungal agents. Beyond classical planktonic resistance mechanisms, *Candida* biofilms present a unique and multifactorial mode of antifungal tolerance that significantly complicates the treatment of vulvovaginal candidiasis [92]. Biofilm-associated cells exhibit phenotypic heterogeneity, including the presence of a subpopulation of persister cells that can survive high antifungal concentrations without acquiring genetic mutations [93,94]. According to Kaur and Nobile [95], these biofilm-resident persisters are particularly enriched during echinocandin and azole treatment and can regrow once drug pressure is removed, contributing to treatment failure and recurrence. Additionally, the biofilm matrix, composed of β-glucans, extracellular DNA, and proteins, acts as a physical and chemical barrier that limits antifungal penetration [46,95]. This is especially relevant in the context of our findings, where micafungin and flucytosine showed partial biofilm eradication at concentrations much higher than MIC values. Biofilm-specific upregulation of stress response pathways and transcriptional regulators (such as *BCR1*, *EFG1*, and *ZAP1*) also plays a role in modulating antifungal tolerance by enhancing matrix production, cell adhesion, and metabolic flexibility [96]. Moreover, efflux pump expression appears to be upregulated specifically in early biofilm phases, contributing to reduced intracellular drug accumulation [95,97]. Taken together, these biofilm-specific resistance mechanisms may help explain the limited efficacy of conventional antifungals observed in our assays and highlight the need for combinatorial or biofilm-targeted therapeutic strategies in managing vaginal *Candida* infections, as urged by several authors [17,46,48,98].

Finally, it is important to mention that only 3 of 21 vaginal isolates were *C. glabrata,* and it is not possible to reach a reliable conclusion; however, these vaginal isolates evidenced a high–intermediate biofilm formation classification, and further evaluation of this *Candida* species should be performed in future studies. Additionally, our findings should be interpreted as representative of this particular study population and not as evidence of a nationwide trend. Our results have also emphasized that resistant isolates were detected even among women with healthy microbiota, highlighting the relevance of colonization as a possible reservoir for resistance, and so future studies need to characterize their resistance mechanisms. This study also analyzed the antifungal resistance in planktonic cells among eight antifungal agents, and, therefore, an additional evaluation of the resistance to the missing antifungals in biofilms of the present group should be carried out in future studies, which constitutes a limitation in the present work. The characterization of *C. albicans* and *C. glabrata* biofilms is currently an important field of research because of the large increase in persistent and severe vaginal infections among women of reproductive age [27], their involvement in mixed vaginal infections, and increased antimicrobial resistance worldwide [22,99]. The present study possesses additional deficiencies, such as the lack of molecular and genomic analyses on *Candida albicans* and *Candida glabrata* biofilms, and the small sample set used in the study does not allow the generalization of the results on antifungal resistance in Ecuadorian women. The use of these commercial antifungals is the most commonly used practice to treat candidiasis in women in Ecuador and worldwide. In general, our results with the different antifungals from various families suggest that echinocandins (anidulafungin and micafungin) constitute a viable alternative in the treatment of candidiasis.

## 5. Conclusions

This study reveals that the majority of vaginal *Candida* isolates in Ecuadorian women exhibit strong biofilm-forming abilities and heightened antifungal resistance, particularly in the biofilm state. Echinocandins, especially micafungin, were the most effective agents for both biofilm inhibition and eradication, while high resistance rates to fluconazole, flucytosine, and amphotericin B were observed across both planktonic and biofilm cells. Overall, our findings emphasize the limitations of standard susceptibility testing and support the incorporation of biofilm-targeted diagnostics to better guide clinical decision making. However, these findings should be interpreted as representative of this particular study set, such as echinocandins constitute an alternative treatment, and not as evidence of a nationwide trend.

By addressing a gap in regional surveillance and focusing on clinically relevant isolates, our study reinforces the need for tailored antifungal strategies in managing vulvovaginal candidiasis. Further genomic and transcriptomic evaluations are needed to elucidate the molecular resistance mechanisms in biofilm-associated infections, inform personalized therapies, and improve treatment outcomes for women affected by these persistent infections.

## Figures and Tables

**Table 1:** Antifungal susceptibility test results for planktonic and biofilm growth of the 21 vaginal *Candida* isolates.

Isolates	Sample	P vs. B *		Fluconazole	Micafungin	Flucytosine
**Healthy Microbiota**						
*C. albicans*	V130	Planktonic	Classification	Sensitive	Resistant	Resistant
			MIC (µg/mL)	4	-	-
		Biofilm	MBIC90 (µg/mL)	128	4	256
			MBEC90 (µg/mL)	128	4	256
*C. albicans*	V134	Planktonic	Classification	Sensitive	Sensitive	Sensitive
			MIC (µg/mL)	1	0.03	4
		Biofilm	MBIC90 (µg/mL)	4	0.50	128
			MBEC90 (µg/mL)	8	0.50	128
*C. albicans*	V196	Planktonic	Classification	Sensitive	Resistant	Resistant
			MIC (µg/mL)	4	-	-
		Biofilm	MBIC90 (µg/mL)	16	1	256
			MBEC90 (µg/mL)	16	1	256
*C. albicans*	V197	Planktonic	Classification	Resistant	Sensitive	Resistant
			MIC (µg/mL)	-	0.03	-
		Biofilm	MBIC90 (µg/mL)	64	0.50	64
			MBEC90 (µg/mL)	64	0.50	128
*C. albicans*	V202	Planktonic	Classification	Sensitive	Resistant	Sensitive
			MIC (µg/mL)	4	-	16
		Biofilm	MBIC90 (µg/mL)	16	-	128
			MBEC90 (µg/mL)	16	-	32
*C. albicans*	V251	Planktonic	Classification	Sensitive	Sensitive	Resistant
			MIC (µg/mL)	4	0.25	-
		Biofilm	MBIC90 (µg/mL)	64	1	256
		Biofilm	MBEC90 (µg/mL)	64	2	512
*C. albicans*	V448	Planktonic	Classification	Resistant	Sensitive	Resistant
			MIC (µg/mL)	-	0.25	-
		Biofilm	MBIC90 (µg/mL)	128	2	-
		Biofilm	MBEC90 (µg/mL)	128	2	-
*C. albicans*	V451	Planktonic	Classification	Sensitive	Resistant	Resistant
			MIC (µg/mL)	2	-	-
		Biofilm	MBIC90 (µg/mL)	64	1	256
		Biofilm	MBEC90 (µg/mL)	64	1	256
*C. albicans*	V580	Planktonic	Classification	Resistant	Resistant	Resistant
			MIC (µg/mL)	-	-	-
		Biofilm	MBIC90 (µg/mL)	-	-	256
			MBEC90 (µg/mL)	-	-	32
**Intermediate Microbiota**						
*C. albicans*-*E. coli*	V118	Planktonic	Classification	Sensitive	Sensitive	Sensitive
			MIC (µg/mL)	2	0.25	16
		Biofilm	MBIC90 (µg/mL)	8	1	256
			MBEC90 (µg/mL)	16	1	512
*C. glabrata*-Gram-positive coccus	V543	Planktonic	Classification	Sensitive	Sensitive	Resistant
			MIC (µg/mL)	8	0.06	-
		Biofilm	MBIC90 (µg/mL)	32	1	128
			MBEC90 (µg/mL)	32	2	128
**Candidiasis**						
*C. albicans*	V161	Planktonic	Classification	Resistant	Resistant	Resistant
			MIC (µg/mL)	-	-	-
		Biofilm	MBIC90 (µg/mL)	-	-	-
			MBEC90 (µg/mL)	-	-	-
*C. albicans*	V218	Planktonic	Classification	Sensitive	Sensitive	Sensitive
			MIC (µg/mL)	4	0.25	8
		Biofilm	MBIC90 (µg/mL)	-	-	512
			MBEC90 (µg/mL)	-	2	512
*C. albicans*	V252	Planktonic	Classification	Sensitive	Sensitive	Resistant
			MIC (µg/mL)	4	0.12	-
		Biofilm	MBIC90 (µg/mL)	64	0.50	128
			MBEC90 (µg/mL)	64	4	256
*C. albicans*	V449	Planktonic	Classification	Sensitive	Sensitive	Sensitive
			MIC (µg/mL)	8	0.12	16
		Biofilm	MBIC90 (µg/mL)	64	2	512
			MBEC90 (µg/mL)	-	4	-
*C. albicans*	V450	Planktonic	Classification	Sensitive	Sensitive	Resistant
			MIC (µg/mL)	4	0.25	-
		Biofilm	MBIC90 (µg/mL)	8	0.50	128
			MBEC90 (µg/mL)	4	0.12	128
*C. albicans*	V535	Planktonic	Classification	Sensitive	Resistant	Sensitive
			MIC (µg/mL)	4	-	32
		Biofilm	MBIC90 (µg/mL)	64	0.25	128
		Biofilm	MBEC90 (µg/mL)	128	0.50	256
*C. albicans*	V540	Planktonic	Classification	Sensitive	Resistant	Sensitive
			MIC (µg/mL)	4	-	32
		Biofilm	MBIC90 (µg/mL)	128	0.50	512
			MBEC90 (µg/mL)	128	1	512
**Mixed Infection**						
*C. albicans* C.-AV	V415	Planktonic	Classification	Sensitive	Sensitive	Sensitive
			MIC (µg/mL)	8	0.12	16
		Biofilm	MBIC90 (µg/mL)	128	4	-
			MBEC90 (µg/mL)	128	-	128
*C. albicans* C.-AV	V527	Planktonic	Classification	Sensitive	Sensitive	Sensitive
			MIC (µg/mL)	2	0.12	16
		Biofilm	MBIC90 (µg/mL)	128	-	-
			MBEC90 (µg/mL)	16	4	128
*C. glabrata* C.-BV	V601	Planktonic	Classification	Resistant	Sensitive	Resistant
			MIC (µg/mL)	-	0.03	-
		Biofilm	MBIC90 (µg/mL)	32	0.50	256
		Biofilm	MBEC90 (µg/mL)	16	2	256

Legend—Classification as susceptible or resistant refers exclusively to the planktonic growth form, determined using the SensiQuattro CANDIDA EU Panel according to EUCAST breakpoints. The antifungal activities of fluconazole, micafungin, and flucytosine were further confirmed by microdilution assays following CLSI and EUCAST guidelines. Hyphens (-) indicate that no inhibition or eradication was observed under the tested conditions. P vs. B *: Planktonic vs. Biofilm; C.: Candidiasis; AV: Aerobic vaginitis; and BV: Bacterial vaginosis.

## Data Availability

The original contributions presented in this study are included in the article and Supplementary Materials. Further inquiries can be directed to the corresponding authors.

## Supplementary Materials

The following supporting information can be downloaded at: https://www.mdpi.com/article/10.3390/jof11090620/s1, Figure S1: Illustration of the antifungal susceptibility and resistance evaluation obtained on planktonic cells of the *Candida* isolates in the present study. The orange squares indicate the concentration at which the antifungal is resistant according to the previously mentioned literature; Figure S2: Correlation between the two absorbances used in the biomass evaluation assays (ABS570 and ABS630); Figure S3: Illustration of the colony-forming units (CFUs) counting in the minimum biofilm inhibitory concentration (MBIC) assays of *Candida albicans* and *Candida glabrata* strains by fluconazole (a), micafungin (b), and flucytosine (c) antifungals. The illustrated statistical analysis between control and MBIC for each *Candida* species. Using the Mann–Whitney U test to create confidence intervals for all pairwise differences, where the bars show the minimum biofilm inhibitory concentration for each outcome, in order to determine whether there is a significant difference between variables (growth control against MBIC values for each isolate), more specifically a *p* < 0.05; b *p* < 0.01; c *p* < 0.001; d *p* < 0.0001; Figure S4: Illustration of the colony-forming unit (CFU) counting in the minimal biofilm eradication concentration (MBEC) assays of *Candida albicans* and *Candida glabrata* strains by fluconazole (a), micafungin (b), and flucytosine (c) antifungals. The illustrated statistical analysis between control and MBEC for each *Candida* species. Using the Mann–Whitney U test to create confidence intervals for all pairwise differences, where the bars show the minimal biofilm eradication concentration for each outcome, in order to determine whether there is a significant difference between variables (growth control against MBEC values for each isolate), more specifically a *p* < 0.05; b *p* < 0.01; c *p* < 0.001; d *p* < 0.0001; Table S1: General information was extracted from data on women in this study with healthy microbiota, intermediate microbiota, candidiasis, and other vaginal infections or dysbiosis, as previously reported by our research group [27]; Table S2: General information was extracted from initial data on women with healthy microbiota, intermediate microbiota, candidiasis, and mixed vaginal infections or dysbiosis; Table S3: EUCAST and CLSI recommended ranges for the classification of susceptible (S) and resistant (R) isolates against different antifungal agents; Table S4: Biofilm classification of *Candida* isolates according to Turan & Demirbilek (2018) [32] and Kıvanç & Er (2020) [1]; Table S5: Overall results of biofilm inhibition assays by biomass and CFU counting methodologies using fluconazole, micafungin, and flucytosine; Table S6: Overall results of biofilm eradication assays by biomass and CFU counting methodologies using fluconazole, micafungin, and flucytosine; Table S7: Overall results of sensitivity of planktonic cells of all clinical isolates using fluconazole, voriconazole, posaconazole, caspafungin, anidulafungin, micafungin, flucytosine and amphotericin B.

## Abbreviations

The following abbreviations are used in this manuscript:

CFU	Colony-forming unit
MIC	Minimum inhibitory concentration
MBIC	Minimum biofilm inhibitory concentration
MBEC	Minimum biofilm eradication concentration
VVC	Vulvovaginal candidiasis
AV	Aerobic vaginitis
BV	Bacterial vaginosis
CV	Crystal violet
PBS	Phosphate-buffered saline
