# Supplementary material for: Impact of Biofilm Formation by Vaginal Candida albicans and Candida glabrata Isolates and Their Antifungal Resistance: A Comprehensive Study in Ecuadorian Women
Source: J Fungi (Basel). 2025 Aug 25;11(9):620. doi: 10.3390/jof11090620 (PMC12470957; doi:10.3390/jof11090620)
Supplement: Supplementary file 1 [file jof-11-00620-s001.zip › jof-3765520-supplementary.pdf]

Supplementary Material

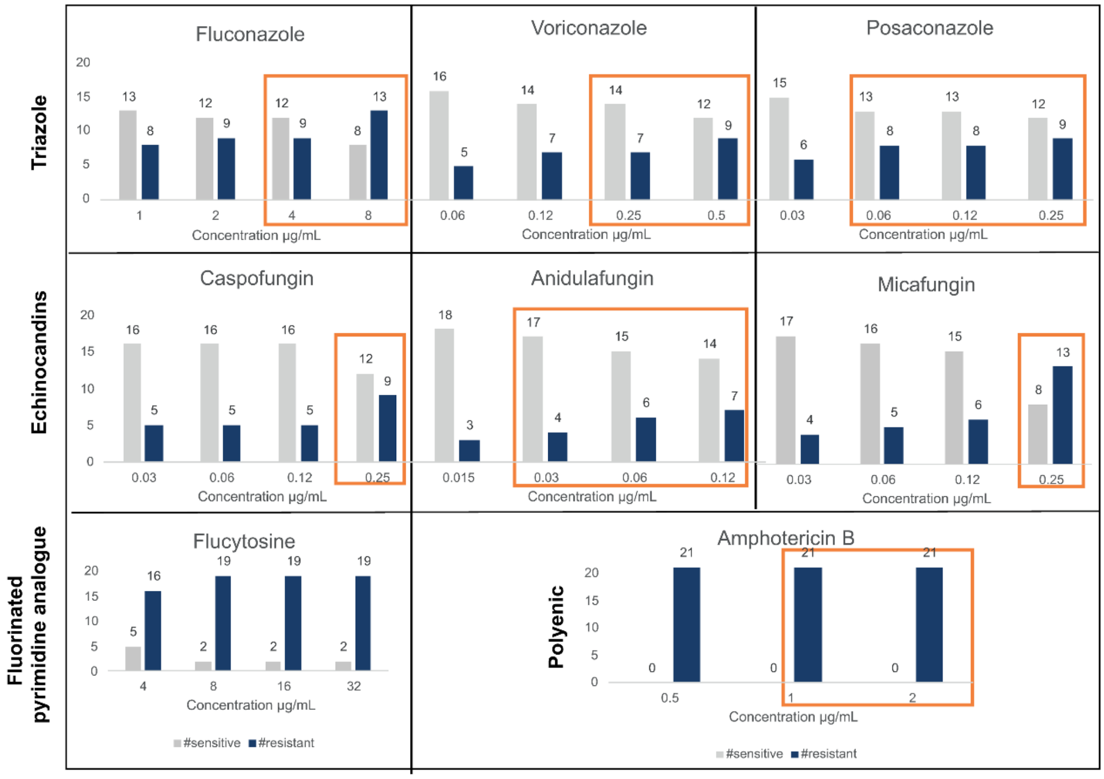

Supplementary Figure S1. Illustration of the antifungal susceptibility and resistance evaluation obtained on planktonic cells of the *Candida* isolates in the present study. The orange squares indicate the concentration at which the antifungal is resistant according to the previously mentioned literature.

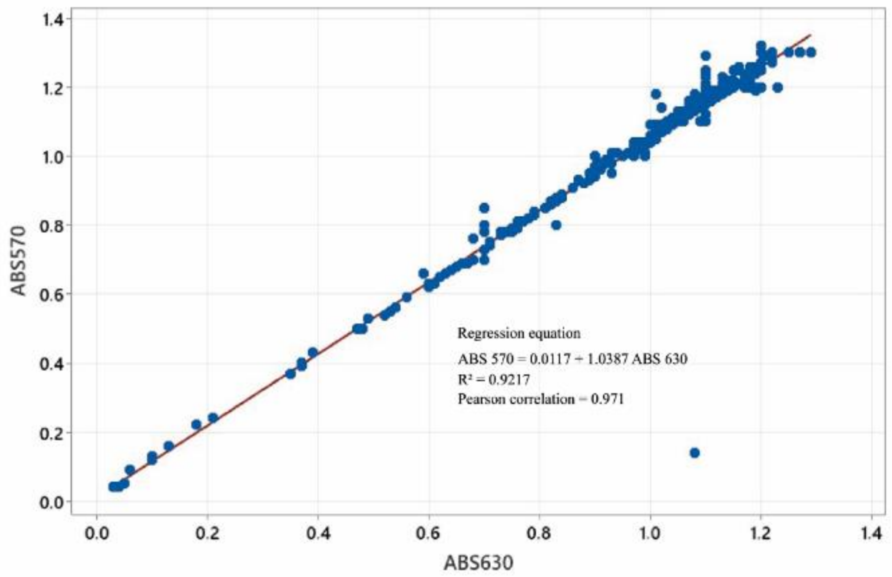

Supplementary Figure S2. Correlation between the two absorbances used in the biomass evaluation assays (ABS570 and ABS630).

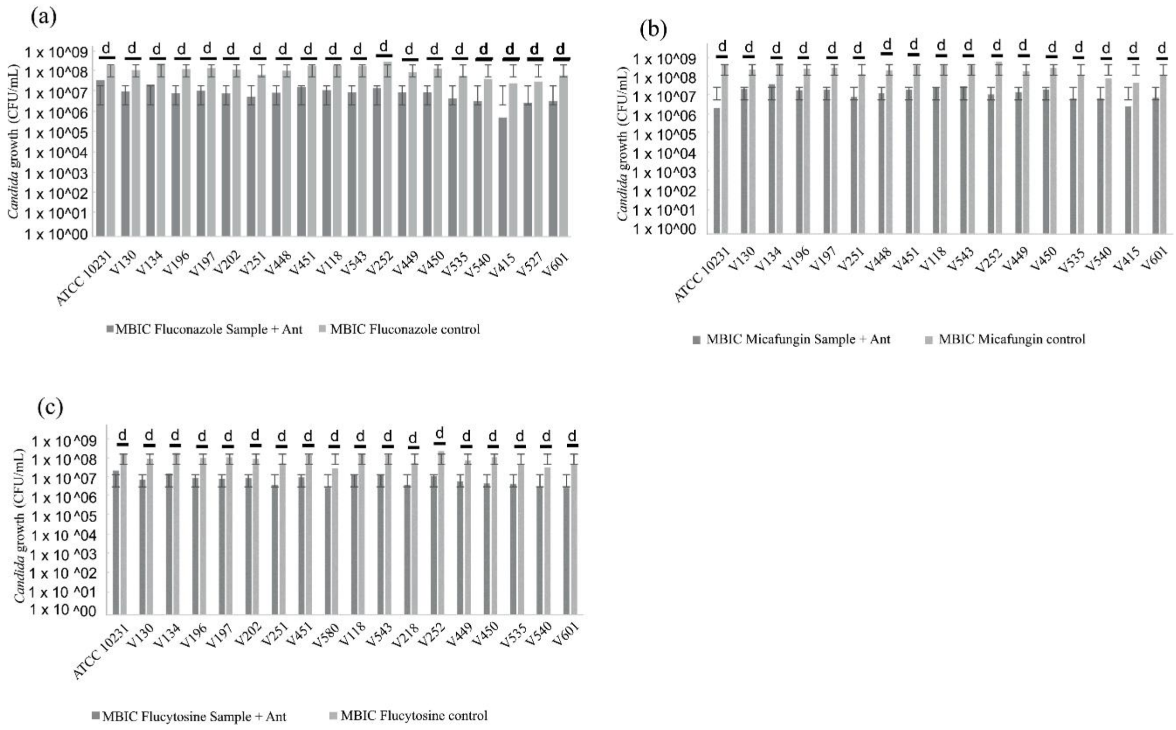

Supplementary Figure S3. Illustration of the colony-forming units (CFU) counting in the minimum biofilm inhibitory concentration (MBIC) assays of *Candida albicans* and *Candida glabrata* strains by fluconazole (a), micafungin (b) and flucytosine (c) antifungals. The illustrated statistical analysis between control and MBIC, for each *Candida* species. Using the Mann-Whitney U test to create confidence intervals for all pairwise differences, where the bars show the minimum biofilm inhibitory concentration for each outcome, in order to determine whether there is a significant difference between variables (growth control against MBIC values for each isolate), more specifically a $p<0.05$; b $p<0.01$; c $p<0.001$; d $p<0.0001$.

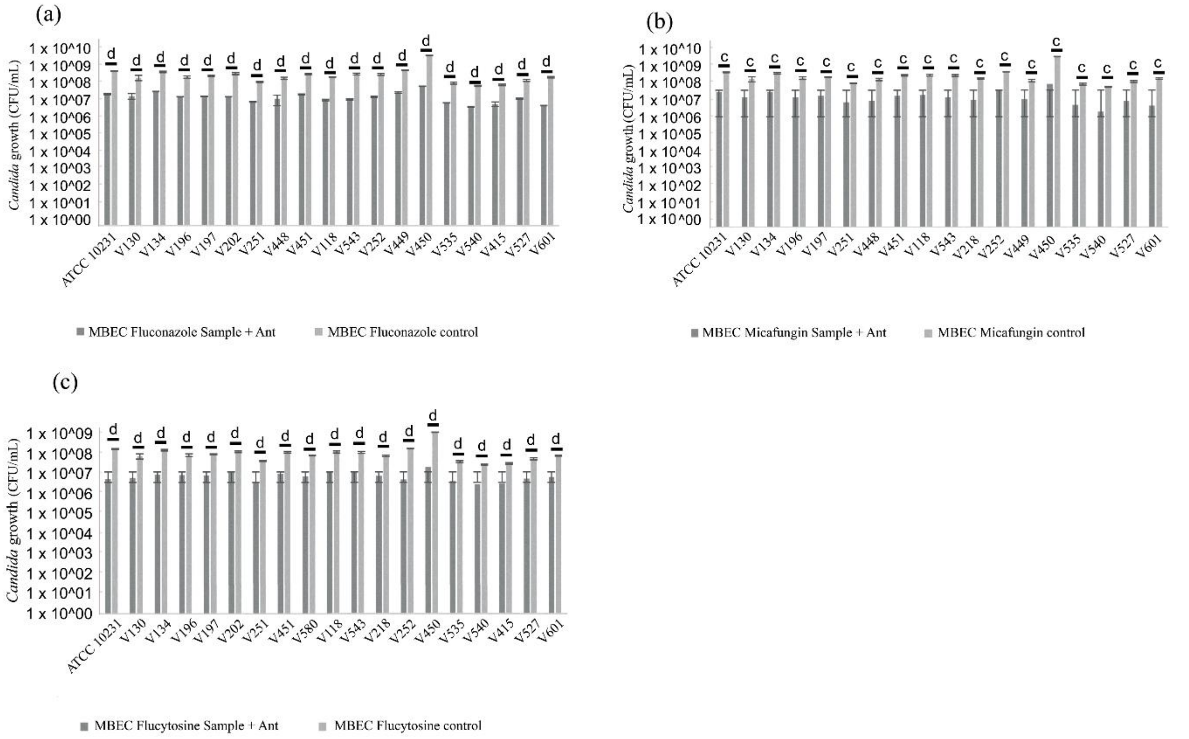

Supplementary Figure S4. Illustration of the colony-forming units (CFU) counting in the minimal biofilm eradication concentration (MBEC) assays of *Candida albicans* and *Candida glabrata* strains by fluconazole (a), micafungin (b) and flucytosine (c) antifungals. The illustrated statistical analysis between control and MBEC, for each *Candida* species. Using the Mann-Whitney U test to create confidence intervals for all pairwise differences, where the bars show the minimal biofilm eradication concentration for each outcome, in order to determine whether there is a significant difference between variables (growth control against MBEC values for each isolate), more specifically a $p<0.05$; b $p<0.01$; c $p<0.001$; d $p<0.0001$.

Supplementary Table S1. General information was extracted from the data of women in the study with healthy microbiota, intermediate microbiota, candidiasis, and other vaginal infections or dysbiosis, as previously reported by our research group [17].

		Healthy microbiota N (%)	Intermediate microbiota N (%)	Candidiasis N (%)	Other infections N (%)	Total N (%)
**Total incidence**		276 (66.7)	43 (10.4)	7 (1.7)	88 (21.3)	414 (100.0)
**Age**	Under 20	57 (20.7)	9 (20.9)	2 (28.6)	21 (23.9)	89 (21.5)
	21-30	175 (63.4)	27 (62.8)	5 (71.4)	49 (55.7)	256 (61.8)
	31-40	27 (9.8)	3 (7.0)	0 (0.0)	10 (11.4)	40 (9.7)
	41-50	13 (4.7)	2 (4.7)	0 (0.0)	3 (3.4)	18 (4.3)
	Over 50	4 (1.4)	2 (4.7)	0 (0.0)	5 (5.7)	11 (2.7)
**Civil status**	Single	229 (83.0)	36 (83.7)	7 (100.0)	71 (80.7)	343 (82.9)
	Free union*	4 (1.4)	1 (2.3)	0 (0.0)	4 (4.5)	9 (2.2)
	Married	39 (14.1)	5 (11.6)	0 (0.0)	9 (10.2)	53 (12.8)
	Divorced	4 (1.4)	1 (2.3)	0 (0.0)	4 (4.5)	9 (2.2)
**Sexual partner**	Not having	101 (36.6)	25 (58.1)	5 (71.4)	37 (42.0)	168 (40.6)
	Having	175 (63.4)	18 (41.9)	2 (28.6)	51 (58.0)	246 (59.4)
**Contraceptive use**	No	101 (36.6)	26 (60.5)	2 (28.6)	33 (37.5)	162 (39.1)
	Yes	175 (63.4)	17 (39.5)	5 (71.4)	55 (62.5)	252 (60.9)
**Birth control methods**	Condom	82 (29.7)	7 (16.3)	4 (57.1)	32 (36.4)	125 (30.2)
	Hormonal contraception	47 (17.0)	2 (4.7)	1 (14.3)	11 (12.5)	61 (14.7)
	Combined	38 (13.8)	6 (14.0)	0 (0.0)	9 (10.2)	53 (12.8)
	Others	8 (2.9)	2 (4.7)	0 (0.0)	3 (3.4)	13 (3.1)
	None or don't answer	101 (36.6)	26 (60.5)	2 (28.6)	33 (37.5)	162 (39.1)

*Free Union: Free Union couples living together for at least 3 years without being married. Epidemiological and behavioral variables among women in the study by [1] with healthy microbiota, intermediate microbiota, candidiasis, and other infections. N number of women who answered the survey within each category; % percentage assigned for each classification.

Supplementary Table S2. General information was extracted from the initial data with healthy microbiota, intermediate microbiota, candidiasis, and mixed vaginal infections or dysbiosis.

Isolates	Sample	Age	Behaviour										
			Sexual partner	Contraceptive use	Number of sexual partners in the last year	Vaginal discharge	Strong odor of vaginal discharge	Number of treatments	Discomfort due to vaginal secretion	Have Children	Medical consultation for infection	Number of treatments during life	Coinfections
**HEALTHY MICROBIOTA**													
C. albicans	V130	33	Yes	HC	1	Yes	Yes	1	Yes	No	No	3	2
C. albicans	V134	25	Yes	Condom	1	No	No	1	No	No	Yes	1	1
C. albicans	V196	22	Yes	Condom	1	Yes	Yes	1	No	No	Yes	NA	1
C. glabrata	V197	21	Yes	HC	1	No	No	0	No	No	No	NA	1
C. albicans	V202	19	No	NA	NA	Yes	No	NA	NA	NA	NA	NA	NA
C. albicans	V251	20	Yes	Condom	1	Yes	No	1	No	No	Yes	1	1
C. albicans	V448	24	No	Condom	2	Yes	No	1	Yes	No	No	NA	1
C. albicans	V451	22	No	Condom	1	Yes	No	NA	No	No	No	NA	1
C. albicans	V580	24	Yes	Condom	NA	NA	NA	NA	NA	No	No	NA	1
**INTERMEDIATE MICROBIOTA**													
C. albicans-E. coli	V118	22	No	None	NA	Yes	NA	NA	NA	NA	NA	NA	1
C. glabrata-Gram positive coccus	V543	20	NA	None	NA	Yes	No	1	No	No	NA	NA	1
**CANDIDIASIS**													
C. albicans	V161	30	Yes	None	1	No	No	2	No	Yes	No	NA	1
C. albicans	V218	23	No	None	NA	Yes	No	1	No	No	No	NA	1
C. albicans	V252	21	NA	HC	NA	Yes	No	NA	No	No	No	NA	1

C. albicans	V449	24	Yes	HC	1	Yes	NA	NA	Yes	Yes	No	NA	1
C. albicans	V450	23	Yes	HC	1	NA	NA	NA	No	No	Yes	1	1
C. albicans	V535	24	No	None	NA	No	No	NA	No	No	No	NA	1
C. albicans	V540	19	No	HC	NA	No	No	1	No	No	No	NA	1
**MIXED INFECTION**													
C. albicans Candidiasis-Aerobic vaginitis	V415	20	No	HC	NA	Yes	Yes	1	Yes	No	Yes	3	1
C. albicans Candidiasis-Aerobic Vaginitis	V527	23	Yes	Condom	>4	Yes	Yes	1	No	No	No	NA	3
C. glabrata Candidiasis-Bacterial vaginosis	V601	21	No	None	NA	Yes	No	NA	No	No	No	NA	1

HC: hormonal birth control. NA: No answer. Behaviour variables of the 21 samples of women in the study with healthy microbiota, intermediate microbiota, candidiasis, and with the presence of mixed infection.

Supplementary Table S3. EUCAST and CLSI recommended ranges for the classification of susceptible (S) and resistant (R) isolates against different antifungal agents.

Family	Antifungal Agent	Species	S ≤ (mg/L)	R > (mg/L)	Reference
Triazoles	Fluconazole	*C. albicans*	2	4	(EUCAST. n.d.)
		C. glabrata	0.001	16	(EUCAST. n.d.)
	Voriconazole	*C. albicans*	0.06	0.25	(EUCAST. n.d.)
		C. glabrata	ND	ND	(EUCAST. n.d.)
	Posaconazole	*C. albicans*	0.06	0.06	(EUCAST. n.d.)
		C. glabrata	ND	ND	(EUCAST. n.d.)
Echinocandins	Caspofungin	*C. albicans*	0.25	1	(Alexander & CLSI. n.d.)
		C. glabrata	0.12	0.5	(Alexander & CLSI. n.d.)
	Anidulafungin	*C. albicans*	0.03	0.03	(EUCAST. n.d.)
		C. glabrata	0.06	0.06	(EUCAST. n.d.)
	Micafungin	*C. albicans*	0.016	0.016	(EUCAST. n.d.)
		C. glabrata	0.03	0.03	(EUCAST. n.d.)
Fluorinated pyrimidine analog	Flucytosine	*C. albicans*	50	150	(Inderbir Padda & Mayur Parmar. 2022)
		C. glabrata	50	150	(Inderbir Padda & Mayur Parmar. 2022)
Polyenic	Amphotericin B	*C. albicans*	1	1	(EUCAST. n.d.)
		C. glabrata	1	1	(EUCAST. n.d.)

Supplementary Table S4. Biofilm classification of *Candida* isolates according to Turan & Demirbilek (2018) [19] and Kıvanç & Er (2020) [1].

#	Isolates	Sample	Biofilm formation capacity according to Turan & Demirbilek (2018) [19]		Biofilm formation capacity according to Kıvanç & Er (2020) [1]		
			PBS OD value 24h (± standard deviation)	Biofilm formation categories	CV OD value 24h (± standard deviation)	CV OD value 48h (± standard deviation)	Biofilm formation categories
**HEALTHY MICROBIOTA**							
1	*C. albicans*	V130	0.69 (± 0.21)	HBF	1.10(± 0.29)	0.23 (± 0.33)	HBF/IBF
2	*C. albicans*	V134	0.71 (± 0.21)	HBF	0.49(± 0.29)	0.40 (± 0.33)	IBF
3	*C. albicans*	V196	0.64 (± 0.21)	HBF	0.75(± 0.29)	0.49 (± 0.33)	HBF/IBF
4	*C. glabrata*	V197	0.53 (± 0.21)	IBF	0.50(± 0.29)	1.48 (± 0.33)	HBF
5	*C. albicans*	V202	0.63 (± 0.21)	HBF	0.88(± 0.29)	0.53 (± 0.33)	HBF
6	*C. albicans*	V251	0.35 (± 0.21)	IBF	0.57(± 0.29)	0.55 (± 0.33)	HBF
7	*C. albicans*	V448	0.68 (± 0.21)	HBF	0.36(± 0.29)	0.38 (± 0.33)	IBF
8	*C. albicans*	V451	0.33 (± 0.21)	IBF	0.24 (± 0.29)	0.29 (± 0.33)	IBF
9	*C. albicans*	V580	1.09 (± 0.21)	HBF	1.12 (± 0.29)	0.59 (± 0.33)	HBF
**INTERMEDIATE MICROBIOTA**							
10	*C. albicans*-*E. coli*	V118	0.99 (± 0.21)	HBF	0.95 (± 0.29)	0.68 (± 0.33)	HBF
11	*C. glabrata*-Gram positive coccus	V543	1.18 (± 0.21)	HBF	1.09 (± 0.29)	0.39 (± 0.33)	HBF/IBF
**CANDIDIASIS**							
12	*C. albicans*	V161	0.14 (± 0.21)	NBF	0.50(± 0.29)	0.69 (± 0.33)	HBF
13	*C. albicans*	V218	0.28 (± 0.21)	LBF	0.68(± 0.29)	0.11 (± 0.33)	HBF/LBF
14	*C. albicans*	V252	0.34 (± 0.21)	IBF	0.61(± 0.29)	0.22 (± 0.33)	HBF/LBF
15	*C. albicans*	V449	0.59 (± 0.21)	HBF	0.56(± 0.29)	0.17 (± 0.33)	HBF/LBF
16	*C. albicans*	V450	0.69 (± 0.21)	HBF	0.45(± 0.29)	0.15 (± 0.33)	IBF/LBF
17	*C. albicans*	V535	0.39 (± 0.21)	IBF	0.62(± 0.29)	0.16 (± 0.33)	HBF/LBF
18	*C. albicans*	V540	0.39 (± 0.21)	IBF	0.65(± 0.29)	1.09 (± 0.33)	HBF
**MIXED INFECTION**							
19	*C. albicans* Candidiasis-Aerobic vaginitis	V415	0.73 (± 0.21)	HBF	0.48(± 0.29)	0.23(± 0.33)	IBF/LBF
20	*C. albicans* Candidiasis-Aerobic Vaginitis	V527	0.49 (± 0.21)	IBF	0.68(± 0.29)	0.82 (± 0.33)	HBF
21	*C. glabrata* Candidiasis-Bacterial vaginosis	V601	1.04 (± 0.21)	HBF	0.86 (± 0.29)	0.41 (± 0.33)	HBF/IBF

HBF: High biofilm formers; IBF: Intermediate biofilm formers; LBF: Low biofilm formers; NBF: non-biofilm formers. Classification of the biofilm formation capacity of the 21 samples analyzed in the study.

Supplementary Table S5. Overall results of biofilm inhibition assays by biomass and CFU counting methodologies using fluconazole, micafungin, and flucytosine.

Biofilm Inhibitory concentration								
**Fluconazole**								
**Reference control strain**	**Parameter**	**Concentration (µg/ml)**						
		**Control**	**4**	**8**	**16**	**32**	**64**	**128**
Candida albicans ATCC 10231	Abs 570 (SD)	1.31 (0.02)	1.28 (0.03)	1.25 (0.02)	1.00 (0.05)	0.80 (0.06)	0.60 (0.05)	0.34 (0.03)
	Abs 630 (SD)	1.28 (0.02)	1.24 (0.03)	1.22 (0.02)	0.97 (0.05)	0.78 (0.06)	0.58 (0.05)	0.31 (0.03)
	Mean CFU (SD)	2.35E+08 (4.85E+07)	2.16E+08 (3.45E+07)	1.35E+08 (3.72E+07)	8.77E+07 (3.03E+06)	7.02E+07 (1.09E+07)	5.61E+07 (1.88E+06)	4.50E+07 (2.03E+06)
	% Inhibition (%SD)	- (20.66)	7.87 (15.96)	42.31 (27.48)	62.63 (3.44)	70.10 (15.58)	76.09 (3.30)	80.84 (4.50)

**Healthy Microbiota**	**Parameter**	**Concentration (µg/ml)**						
		**Control**	**4**	**8**	**16**	**32**	**64**	**128**
Candida albicans (V130)	Abs 570 (SD)	1.23 (0.014)	1.22 (0.008)	1.24 (0.02)	1.21 (0.006)	1.18 (0.015)	1.17 (0.02)	1.12 (0.02)
	Abs 630 (SD)	1.18 (0.012)	1.17 (0.009)	1.19 (0.020)	1.16 (0.007)	1.13 (0.018)	1.12 (0.017)	1.07 (0.02)
	Mean CFU (SD)	1.45E+08 (4.62E+07)	6.16E+07 (1.76E+07)	3.09E+07 (1.59E+07)	2.92E+07 (1.18E+07)	3.10E+07 (1.77E+07)	2.95E+07 (1.97E+07)	1.28E+07 (3.89E+06)
	% Inhibition (%SD)	- (31.93)	57.46 (28.54)	78.68 (51.48)	79.86 (40.48)	78.57 (57.13)	79.66 (66.89)	91.18 (30.46)
Candida albicans (V134)	Abs 570 (SD)	1.18 (0.01)	1.12 (0.02)	1.16 (0.03)	1.09 (0.003)	0.89 (0.02)	0.67 (0.005)	0.53 (0.003)
	Abs 630 (SD)	1.13 (0.01)	1.07 (0.02)	1.10 (0.03)	1.04 (0.003)	0.84 (0.01)	0.64 (0.004)	0.49 (0.004)
	Mean CFU (SD)	2.96E+08 (2.84E+07)	2.84E+07 (1.14E+06)	2.27E+07 (2.25E+05)	1.94E+07 (8.38E+05)	1.08E+07 (3.00E+05)	4.41E+06 (2.13E+05)	2.39E+06 (8.75E+04)
	% Inhibition (%SD)	- (9.58)	90.41 (4.00)	92.35 (0.99)	93.44 (4.31)	96.35 (2.78)	98.51 (4.81)	99.19 (3.66)
Candida albicans (V196)	Abs 570 (SD)	1.20 (0.08)	1.15 (0.009)	1.17 (0.02)	0.81 (0.43)	0.78 (0.40)	0.98 (0.07)	0.95 (0.03)
	Abs 630 (SD)	1.12 (0.07)	1.08 (0.008)	1.09 (0.02)	0.76 (0.40)	0.73 (0.38)	0.91 (0.06)	0.93 (0.06)
	Mean CFU (SD)	1.62E+08	2.30E+07	1.77E+07	1.08E+07	6.79E+06	5.36E+06	1.84E+06

**Healthy Microbiota**	**Parameter**	**Concentration (µg/ml)**						
		**Control**	**4**	**8**	**16**	**32**	**64**	**128**
Candida albicans (V251)	Abs 570 (SD)	1.24 (0.03)	1.23 (0.009)	1.25 (0.02)	1.22 (0.014)	1.19 (0.007)	1.18 (0.02)	1.13 (0.02)
	Abs 630 (SD)	1.19 (0.01)	1.18 (0.007)	1.20 (0.018)	1.17 (0.009)	1.14 (0.016)	1.13 (0.015)	1.08 (0.02)
	Mean CFU (SD)	8.34E+07 (5.35E+06)	4.81E+07 (1.36E+06)	1.40E+07 (3.34E+06)	1.44E+07 (6.00E+05)	8.39E+06 (1.51E+06)	7.01E+06 (1.21E+06)	4.99E+06 (1.63E+05)
	% Inhibition (%SD)	- (6.41)	42.31 (2.83)	83.17 (23.78)	82.73 (4.17)	89.94 (18.03)	91.59 (17.29)	94.02 (3.26)
Candida albicans (V448)	Abs 570 (SD)	1.17 (0.01)	1.11 (0.02)	1.15 (0.03)	1.08 (0.002)	0.87 (0.02)	0.66 (0.004)	0.50 (0.003)
	Abs 630 (SD)	1.12 (0.01)	1.06 (0.02)	1.09 (0.03)	1.03 (0.003)	0.82 (0.01)	0.63 (0.004)	0.47 (0.004)
	Mean CFU (SD)	1.33E+08 (1.73E+07)	7.14E+07 (1.02E+07)	5.02E+07 (5.34E+06)	4.70E+07 (5.73E+06)	2.21E+07 (2.23E+06)	1.62E+07 (2.00E+05)	1.13E+07 (1.41E+06)
	% Inhibition (%SD)	- (12.99)	46.37 (14.31)	62.31 (10.63)	64.69 (12.17)	83.44 (10.09)	87.87 (1.23)	91.48 (12.45)
Candida albicans (V451)	Abs 570 (SD)	1.21 (0.07)	1.16 (0.007)	1.18 (0.02)	0.87 (0.33)	0.84 (0.30)	0.75 (0.05)	0.7 (0.01)
	Abs 630 (SD)	1.13 (0.06)	1.07 (0.006)	1.08 (0.02)	0.82 (0.30)	0.79 (0.28)	0.7 (0.04)	0.69 (0.04)
	Mean CFU (SD)	2.30E+08 (1.84E+07)	1.15E+08	7.63E+07	5.75E+07	4.52E+07	2.18E+07	1.34E+07 (2.61E+06)

		(2.80E+07)	(1.83E+06)	(5.38E+05)	(4.75E+05)	(4.38E+05)	(4.63E+05)	(8.13E+05)				(1.14E+07)	(1.45E+07)	(1.01E+06)	(2.69E+06)	(2.56E+06)	
	% Inhibition (%SD)	- (17.31)	85.77 (7.92)	89.09 (3.04)	93.34 (4.40)	95.80 (6.44)	96.68 (8.62)	98.86 (44.21)		% Inhibition (%SD)	- (7.98)	49.85 (9.88)	66.80 (19.04)	74.99 (1.76)	80.36 (5.95)	90.51 (11.74)	94.17 (19.51)
Candida glabrata (V197)	Abs 570 (SD)	1.07 (0.02)	0.73 (0.08)	0.73 (0.03)	0.39 (0.17)	0.54 (0.07)	0.36 (0.15)	0.26 (0.0075)	*Candida albicans* (V580)	Abs 570 (SD)	1.08 (0.02)	1.11 (0.03)	1.13 (0.02)	1.03 (0.06)	1.03 (0.10)	1.02 (0.06)	0.98 (0.07)
	Abs 630 (SD)	1.01 (0.02)	0.70 (0.08)	0.70 (0.03)	0.37 (0.16)	0.52 (0.07)	0.33 (0.15)	0.24 (0.007)		Abs 630 (SD)	1.04 (0.02)	1.05 (0.03)	1.08 (0.02)	0.97 (0.05)	0.98 (0.10)	0.97 (0.05)	0.93 (0.07)
	Mean CFU (SD)	1.77E+08 (8.53E+06)	6.70E+07 (1.06E+07)	6.59E+07 (1.07E+07)	3.43E+07 (1.64E+07)	1.91E+07 (3.36E+06)	1.37E+07 (9.75E+05)	4.76E+06 (3.44E+06)		Mean CFU (SD)	4.54E+07 (6.63E+06)	2.95E+07 (1.43E+06)	2.11E+07 (6.38E+05)	4.33E+07 (1.36E+06)	3.80E+07 (4.31E+06)	3.85E+07 (4.75E+06)	5.85E+06 (8.50E+05)
	% Inhibition (%SD)	- (4.81)	62.18 (15.87)	62.77 (16.27)	80.60 (47.79)	89.20 (17.59)	92.26 (7.11)	97.31 (7.17)		% Inhibition (%SD)	- (14.60)	34.98 (4.83)	53.47 (3.01)	4.60 (3.14)	16.17 (11.3)	15.15 (12.3)	87.10 (14.5)
Candida albicans (V202)	Abs 570 (SD)	1.11 (0.03)	1.1 (0.009)	1 (0.02)	0.92 (0.01)	0.89 (0.04)	0.88 (0.04)	0.75 (0.02)									
	Abs 630 (SD)	1.04 (0.03)	1.02 (0.008)	1.01 (0.02)	0.9 (0.01)	0.82 (0.03)	0.82 (0.03)	0.73 (0.02)									
	Mean CFU (SD)	1.46E+08 (7.85E+06)	1.78E+07 (9.38E+05)	1.49E+07 (8.13E+05)	1.03E+07 (7.38E+05)	5.44E+06 (2.63E+05)	4.91E+06 (3.13E+05)	4.11E+06 (1.88E+05)									
	% Inhibition (%SD)	- (5.37)	87.79 (5.25)	89.82 (5.46)	92.95 (7.16)	96.27 (4.82)	96.63 (6.36)	97.18 (4.55)									
**Intermediate Microbiota**	**Parameter**	**Control**	**4**	**8**	**16**	**32**	**64**	**128**	**Intermediate Microbiota**	**Parameter**	**Control**	**4**	**8**	**16**	**32**	**64**	**128**
Candida albicans - Escherichia coli (V118)	Abs 570 (SD)	1.23 (0.04)	1.21 (0.01)	1.22 (0.02)	1.04 (0.007)	0.24 (0.02)	0.22 (0.02)	0.16 (0.04)	*Candida albicans - Gram positive coccus* (V543)	Abs 570 (SD)	1.26 (0.02)	1.22 (0.01)	1.25 (0.01)	1.19 (0.04)	1.17 (0.03)	1.1 (0.05)	1 (0.02)
	Abs 630 (SD)	1.17 (0.04)	1.16 (0.01)	1.18 (0.02)	0.98 (0.008)	0.21 (0.02)	0.18 (0.02)	0.13 (0.04)		Abs 630 (SD)	1.18 (0.02)	1.15 (0.01)	1.15 (0.009)	1.12 (0.01)	1.1 (0.01)	1.09 (0.04)	1.05 (0.01)
	Mean CFU (SD)	2.37E+08 (2.82E+07)	2.72E+07 (2.03E+06)	1.52E+07 (1.40E+06)	7.61E+06 (1.88E+05)	3.44E+06 (1.63E+05)	1.24E+06 (8.75E+04)	3.88E+05 (1.13E+05)		Mean CFU (SD)	2.25E+08 (2.58E+07)	5.84E+07 (2.31E+06)	2.72E+07 (6.88E+05)	2.89E+07 (8.88E+05)	1.16E+07 (1.70E+06)	4.76E+06 (5.13E+05)	2.73E+06 (2.75E+05)
	% Inhibition (%SD)	- (11.90)	88.51 (7.43)	93.58 (9.21)	96.78 (2.46)	98.55 (4.72)	99.47 (7.07)	99.83 (29.03)		% Inhibition (%SD)	- (11.46)	73.99 (3.95)	87.90 (2.52)	87.14 (3.07)	94.86 (14.71)	97.88 (10.76)	98.78 (10.09)
**Candidiasis**	**Parameter**	**Control**	**4**	**8**	**16**	**32**	**64**	**128**	**Candidiasis**	**Parameter**	**Control**	**4**	**8**	**16**	**32**	**64**	**128**
	Abs 570 (SD)	1.07 (0.09)	0.98 (0.02)	1.01 (0.004)	0.97 (0.01)	1.01 (0.04)	0.98 (0.01)	0.99 (0.01)		Abs 570 (SD)	1.35 (0.02)	1.27 (0.01)	1.26 (0.01)	1.21 (0.02)	1.21 (0.02)	1.2 (0.05)	1.16 (0.06)

Candida albicans (V161)	Abs 630 (SD)	0.95 (0.01)	0.91 (0.02)	0.94 (0.006)	0.90 (0.01)	0.93 (0.03)	0.91 (0.01)	0.92 (0.008)	*Candida albicans* (V450)	Abs 630 (SD)	1.27 (0.02)	1.20 (0.02)	1.19 (0.01)	1.14 (0.02)	1.14 (0.02)	1.14 (0.05)	1.09 (0.06)
	Mean CFU (SD)	1.20E+08 (2.33E+07)	9.67E+07 (1.42E+07)	6.12E+07 (2.08E+06)	5.72E+07 (2.34E+06)	6.94E+07 (1.26E+07)	4.90E+07 (1.03E+06)	4.61E+07 (1.04E+06)		Mean CFU (SD)	1.71E+08 (1.54E+07)	5.46E+07 (1.29E+07)	1.17E+07 (9.88E+05)	1.17E+07 (1.50E+05)	1.46E+07 (2.63E+06)	1.17E+07 (3.30E+06)	1.01E+07 (2.18E+06)
	% Inhibition (%SD)	- (19.45)	19.16 (14.69)	48.83 (3.39)	52.14 (4.08)	41.97 (18.12)	59.03 (2.09)	61.43 (2.24)		% Inhibition (%SD)	- (9.01)	68.03 (23.53)	93.12 (8.41)	93.14 (1.28)	91.43 (17.94)	93.17 (28.32)	94.11 (21.64)
Candida albicans (V218)	Abs 570 (SD)	1.19 (0.02)	1.18 (0.009)	1.21 (0.007)	1.16 (0.01)	1.19 (0.01)	1.19 (0.01)	1.17 (0.02)	*Candida albicans* (V535)	Abs 570 (SD)	1.22 (0.02)	1.2 (0.009)	1.17 (0.007)	1.17 (0.01)	1.18 (0.01)	1.15 (0.01)	1.14 (0.02)
	Abs 630 (SD)	1.11 (0.03)	1.10 (0.01)	1.13 (0.01)	1.08 (0.01)	1.12 (0.02)	1.11 (0.01)	1.09 (0.03)		Abs 630 (SD)	1.14 (0.03)	1.12 (0.01)	1.10 (0.01)	1.09 (0.01)	1.10 (0.02)	1.09 (0.01)	1.07 (0.03)
	Mean CFU (SD)	8.86E+07 (9.83E+06)	4.73E+07 (6.49E+06)	2.10E+07 (2.88E+06)	3.15E+07 (1.95E+06)	1.11E+07 (1.88E+06)	1.31E+07 (2.68E+06)	1.39E+07 (2.85E+06)		Mean CFU (SD)	7.42E+07 (8.31E+06)	4.57E+07 (6.00E+05)	1.74E+07 (1.06E+06)	1.14E+07 (1.60E+06)	1.22E+07 (2.69E+06)	6.01E+06 (2.38E+05)	5.41E+06 (4.13E+05)
	% Inhibition (%SD)	- (11.08)	46.64 (13.71)	76.30 (13.69)	64.45 (6.19)	87.50 (16.93)	85.24 (20.45)	84.37 (20.57)		% Inhibition (%SD)	- (11.20)	38.36 (1.31)	76.59 (6.11)	84.63 (14.03)	83.57 (22.05)	91.89 (3.95)	92.70 (7.62)
Candida albicans (V252)	Abs 570 (SD)	0.97 (0.23)	0.93 (0.16)	0.92 (0.20)	0.94 (0.18)	0.93 (0.19)	0.85 (0.21)	0.87 (0.21)	*Candida albicans* (V540)	Abs 570 (SD)	1.16 (0.03)	1.14 (0.01)	1.08 (0.01)	1.09 (0.01)	1.09 (0.04)	1.04 (0.03)	0.96 (0.02)
	Abs 630 (SD)	0.93 (0.14)	0.89 (0.21)	0.88 (0.19)	0.90 (0.17)	0.89 (0.17)	0.81 (0.20)	0.83 (0.19)		Abs 630 (SD)	1.08 (0.03)	1.02 (0.01)	1.02 (0.01)	1.04 (0.01)	1.03 (0.04)	0.98 (0.04)	0.91 (0.02)
	Mean CFU (SD)	3.67E+08 (8.16E+06)	1.70E+08 (7.64E+06)	1.64E+08 (1.24E+07)	5.09E+07 (1.40E+07)	3.68E+07 (3.83E+06)	1.88E+07 (2.58E+06)	9.95E+06 (1.15E+06)		Mean CFU (SD)	5.24E+07 (3.03E+06)	1.70E+07 (3.50E+05)	1.49E+07 (7.75E+05)	1.09E+07 (1.50E+05)	7.55E+06 (2.75E+05)	9.09E+06 (2.13E+05)	4.41E+06 (1.13E+05)
	% Inhibition (%SD)	- (2.22)	53.64 (4.48)	55.31 (7.53)	86.14 (27.42)	89.98 (10.39)	94.89 (13.73)	97.29 (11.5)		% Inhibition (%SD)	- (5.77)	67.63 (2.06)	71.50 (5.19)	79.28 (1.38)	85.58 (3.64)	82.64 (2.33)	91.57 (2.54)
Candida albicans (V449)	Abs 570 (SD)	1.18 (0.07)	1.10 (0.17)	1.10 (0.15)	1.03 (0.18)	1.03 (0.20)	1.05 (0.20)	1.02 (0.20)									
	Abs 630 (SD)	1.13 (0.07)	1.06 (0.16)	1.06 (0.14)	1 (0.17)	0.99 (0.17)	1 (0.19)	0.98 (0.19)									
	Mean CFU (SD)	1.16E+08 (1.72E+07)	7.08E+07 (1.39E+07)	5.34E+07 (3.13E+05)	2.09E+07 (1.64E+06)	1.57E+07 (3.88E+05)	1.14E+07 (1.36E+06)	9.18E+06 (2.25E+05)									
	% Inhibition (%SD)	- (14.80)	38.96 (19.64)	53.95 (0.58)	81.98 (7.83)	86.48 (2.47)	90.18 (11.96)	92.09 (2.45)									
**Mixed Infection**	**Parameter**	**Control**	**4**	**8**	**16**	**32**	**64**	**128**	**Mixed Infection**	**Parameter**	**Control**	**4**	**8**	**16**	**32**	**64**	**128**

Candida albicans Candidiasis - *Aerobic vaginitis* (V415)	Abs 570 (SD)	1.12 (0.03)	1.08 (0.05)	1.11 (0.02)	1.07 (0.06)	1.04 (0.09)	0.96 (0.02)	0.91 (0.07)
	Abs 630 (SD)	1.07 (0.03)	1.03 (0.03)	1.04 (0.03)	1.02 (0.04)	1.00 (0.07)	0.91 (0.03)	0.86 (0.05)
	Mean CFU (SD)	3.22E+07 (4.79E+06)	8.08E+06 (1.50E+05)	6.81E+06 (1.88E+05)	4.91E+06 (8.75E+04)	4.50E+06 (2.50E+04)	3.91E+06 (1.13E+05)	6.88E+05 (3.13E+05)
	% Inhibition (%SD)	- (14.86)	74.93 (1.85)	78.85 (2.75)	84.74 (1.78)	86.03 (0.55)	87.85 (2.87)	97.86 (45.45)
Candida albicans Candidiasis - *Aerobic vaginitis* (V527)	Abs 570 (SD)	1.28 (0.03)	1.05 (0.07)	0.82 (0.37)	0.79 (0.34)	0.85 (0.06)	0.88 (0.19)	0.79 (0.17)
	Abs 630 (SD)	1.21 (0.03)	1.01 (0.06)	0.78 (0.36)	0.75 (0.32)	0.81 (0.05)	0.84 (0.18)	0.76 (0.16)
	Mean CFU (SD)	3.94E+07 (1.50E+06)	8.48E+06 (2.25E+05)	7.90E+06 (3.50E+05)	4.05E+06 (2.50E+05)	4.09E+06 (1.13E+05)	4.09E+06 (5.38E+05)	3.64E+06 (5.13E+05)
	% Inhibition (%SD)	- (3.80)	78.50 (2.65)	79.96 (4.43)	89.72 (6.17)	89.63 (2.75)	89.63 (13.14)	90.77 (14.08)
Candida glabrata Candidiasis - *Aerobic vaginitis* (V601)	Abs 570 (SD)	1.21 (0.01)	1.20 (0.007)	1.21 (0.008)	1.21 (0.005)	1.19 (0.02)	0.09 (0.015)	0.04 (0.001)
	Abs 630 (SD)	1.15 (0.008)	1.14 (0.005)	1.15 (0.007)	1.15 (0.006)	1.14 (0.01)	0.06 (0.01)	0.03 (0.001)
	Mean CFU (SD)	7.83E+07 (7.21E+06)	3.52E+07 (1.29E+06)	1.11E+07 (2.89E+06)	9.14E+06 (1.59E+06)	4.34E+06 (1.13E+05)	3.56E+06 (6.25E+04)	7.63E+05 (1.25E+04)
	% Inhibition (%SD)	- (9.20)	55.11 (3.66)	85.78 (25.92)	88.33 (17.37)	94.46 (2.59)	95.45 (1.75)	99.02 (1.63)

Biofilm Inhibitory concentration								
Micafungin								
Reference control strain	Parameter	Concentration (µg/ml)						
		Control	0.15	0.3	0.6	1	2	4
Candida albicans ATCC 10231	Abs 570 (SD)	1.34 (0.02)	1.31 (0.03)	1.35 (0.02)	1.33 (0.02)	1.31 (0.03)	0.43 (0.01)	0.44 (0.14)
	Abs 630 (SD)	1.31 (0.02)	1.28 (0.03)	1.32 (0.02)	1.30 (0.02)	1.28 (0.03)	0.43 (0.01)	0.41 (0.13)
	Mean CFU (SD)	2.35E+08 (4.85E+07)	4.41E+07 (1.24E+07)	4.25E+07 (1.37E+07)	2.85E+07 (2.50E+04)	2.69E+07 (1.26E+06)	1.83E+06 (1.28E+06)	1.13E+05 (3.75E+04)
	% Inhibition (%SD)	- (20.66)	81.21 (28.11)	81.89 (32.13)	87.87 (0.08)	88.54 (4.69)	99.22 (69.86)	99.95 (33.33)

Healthy Microbiota	Parameter	Concentration (µg/ml)							Healthy Microbiota	Parameter	Concentration (µg/ml)						
		Control	0.15	0.3	0.6	1	2	4			Control	0.15	0.3	0.6	1	2	4
Candida albicans (V130)	Abs 570 (SD)	1.29 (0.015)	1.24 (0.013)	1.24 (0.007)	1.24 (0.009)	1.2 (0.01)	1.2 (0.01)	1.23 (0.01)	*Candida albicans* (V251)	Abs 570 (SD)	1.2 (0.01)	1.14 (0.008)	1.162 (0.01)	1.15 (0.01)	1 (0.02)	1.1 (0.01)	0.99 (0.02)
	Abs 630 (SD)	1.2 (0.01)	1.1 (0.01)	1.1 (0.007)	1.1 (0.02)	1.19 (0.01)	1.1 (0.01)	1.1 (0.01)		Abs 630 (SD)	1.11 (0.01)	1.09 (0.006)	1.1 (0.01)	1.09 (0.01)	1 (0.02)	1.1 (0.01)	0.98 (0.02)
	Mean CFU (SD)	1.45E+08 (4.62E+07)	8.05E+07 (1.65E+07)	4.03E+07 (8.41E+06)	2.34E+07 (3.38E+05)	2.34E+07 (4.31E+06)	1.77E+07 (1.58E+06)	1.55E+07 (4.25E+06)		Mean CFU (SD)	8.34E+07 (5.35E+06)	5.97E+07 (1.32E+07)	4.24E+07 (1.26E+06)	1.43E+07 (8.25E+05)	6.23E+06 (2.43E+06)	8.65E+06 (1.15E+06)	4.60E+06 (5.25E+05)
	% Inhibition (%SD)	- (31.93)	44.37 (20.45)	72.13 (20.85)	83.82 (1.44)	83.81 (18.4)	87.79 (8.91)	89.29 (27.41)		% Inhibition (%SD)	- (6.41)	28.38 (22.14)	49.14 (2.97)	82.88 (5.77)	92.53 (38.95)	89.62 (13.29)	94.48 (11.41)
Candida albicans (V134)	Abs 570 (SD)	1.2 (0.01)	1.19 (0.008)	1.16 (0.01)	1.15 (0.01)	1.1 (0.02)	1.1 (0.01)	1.1 (0.02)	*Candida albicans* (V448)	Abs 570 (SD)	1.08 (0.02)	0.59 (0.07)	0.70 (0.10)	0.65 (0.20)	0.78 (0.15)	0.69 (0.16)	0.68 (0.04)
	Abs 630 (SD)	1.17 (0.01)	1.11 (0.006)	1.1 (0.01)	1.09 (0.01)	1 (0.02)	1 (0.01)	0.97 (0.02)		Abs 630 (SD)	1.02 (0.02)	0.56 (0.07)	0.68 (0.10)	0.62 (0.20)	0.75 (0.15)	0.66 (0.16)	0.65 (0.04)
	Mean CFU (SD)	2.96E+08 (2.84E+07)	6.22E+07 (1.35E+07)	4.71E+07 (8.25E+05)	2.78E+07 (1.32E+07)	1.45E+07 (1.78E+06)	1.20E+07 (1.50E+05)	7.58E+06 (5.75E+05)		Mean CFU (SD)	1.33E+08 (1.73E+07)	7.60E+07 (7.25E+05)	4.85E+07 (9.75E+05)	4.46E+07 (9.63E+05)	1.37E+07 (1.49E+06)	9.48E+06 (4.00E+05)	7.71E+06 (2.21E+06)
	% Inhibition (%SD)	- (9.58)	79.00 (21.63)	84.11 (1.75)	90.62 (47.36)	95.09 (12.22)	95.96 (1.25)	97.44 (7.59)		% Inhibition (%SD)	- (12.99)	42.91 (0.95)	63.62 (2.01)	66.52 (2.15)	89.68 (10.82)	92.88 (4.22)	94.20 (28.68)
Candida albicans (V196)	Abs 570 (SD)	1.19 (0.06)	1.13 (0.03)	1.15 (0.03)	1.13 (0.04)	1.16 (0.03)	1.18 (0.03)	1.14 (0.04)	*Candida albicans* (V451)	Abs 570 (SD)	1.12 (0.02)	1.09 (0.02)	1.11 (0.02)	1.07 (0.02)	1.09 (0.03)	1.11 (0.03)	1.06 (0.06)
	Abs 630 (SD)	1.1 (0.06)	1.05 (0.03)	1.07 (0.03)	1.05 (0.04)	1.09 (0.04)	1.01 (0.03)	1.07 (0.04)		Abs 630 (SD)	1.06 (0.02)	1.03 (0.02)	1.05 (0.02)	1.01 (0.02)	1.03 (0.03)	1.05 (0.03)	1.00 (0.06)

	Mean CFU (SD)	1.62E+08 (2.80E+07)	7.80E+07 (1.36E+06)	5.18E+07 (1.19E+06)	2.13E+07 (1.25E+05)	1.38E+07 (2.13E+05)	7.40E+06 (1.48E+06)	4.31E+06 (1.88E+05)		Mean CFU (SD)	2.30E+08 (1.84E+07)	5.29E+07 (1.04E+06)	4.78E+07 (6.75E+05)	5.09E+07 (4.64E+06)	1.40E+07 (2.66E+06)	9.39E+06 (1.11E+06)	3.98E+06 (1.50E+05)
	% Inhibition (%SD)	- (17.31)	51.83 (1.74)	68.03 (2.29)	86.82 (0.58)	91.48 (1.54)	95.42 (19.93)	97.33 (4.34)		% Inhibition (%SD)	- (7.98)	76.99 (1.96)	79.20 (1.41)	77.87 (9.11)	93.89 (18.96)	95.91 (11.85)	98.27 (3.77)
Candida glabrata (V197)	Abs 570 (SD)	1.08 (0.02)	0.59 (0.07)	0.70 (0.10)	0.65 (0.20)	0.65 (0.15)	0.61 (0.16)	0.58 (0.04)	*Candida albicans* (V580)	Abs 570 (SD)	1.21 (0.003)	1.21 (0.01)	1.14 (0.04)	1.18 (0.03)	1.20 (0.19)	1.19 (0.02)	1.21 (0.02)
	Abs 630 (SD)	1.02 (0.02)	0.56 (0.07)	0.68 (0.10)	0.62 (0.20)	0.62 (0.15)	0.6 (0.16)	0.55 (0.04)		Abs 630 (SD)	1.15 (0.002)	1.15 (0.01)	1.09 (0.05)	1.13 (0.03)	1.14 (0.02)	1.13 (0.02)	1.15 (0.02)
	Mean CFU (SD)	1.77E+08 (8.53E+06)	3.65E+07 (1.47E+07)	2.76E+07 (1.51E+07)	1.43E+07 (7.13E+05)	1.33E+07 (1.59E+06)	1.22E+07 (6.25E+04)	9.84E+06 (2.61E+06)		Mean CFU (SD)	4.54E+07 (6.63E+06)	4.52E+07 (5.13E+05)	4.22E+07 (9.00E+05)	3.80E+07 (1.14E+07)	4.03E+07 (9.25E+05)	6.73E+07 (2.61E+07)	4.55E+07 (1.22E+07)
	% Inhibition (%SD)	- (4.81)	79.40 (40.21)	84.42 (54.85)	91.91 (4.97)	92.49 (11.94)	93.11 (0.51)	94.44 (26.55)		% Inhibition (%SD)	- (14.60)	0.30 (1.13)	7.10 (2.13)	16.25 (30)	11.23 (2.29)	48.31 (38)	0.16 (26)
Candida albicans (V202)	Abs 570 (SD)	1.12 (0.02)	1.09 (0.02)	1.1 (0.02)	1.07 (0.02)	1.11 (0.03)	1.1 (0.03)	1.06 (0.06)									
	Abs 630 (SD)	1.06 (0.02)	1.03 (0.02)	1.05 (0.02)	1.01 (0.02)	1.05 (0.03)	1.05 (0.03)	1.00 (0.06)									
	Mean CFU (SD)	1.46E+08 (7.85E+06)	9.98E+07 (4.88E+07)	1.11E+08 (1.63E+07)	9.03E+07 (2.87E+07)	9.47E+07 (2.93E+06)	6.65E+07 (2.35E+07)	5.10E+07 (1.27E+07)									
	% Inhibition (%SD)	- (5.37)	31.68 (48.92)	24.23 (14.77)	38.20 (31.74)	35.16 (3.08)	54.46 (35.28)	65.12 (24.92)									
**Intermediate Microbiota**	**Parameter**	**Control**	**0.15**	**0.3**	**0.6**	**1**	**2**	**4**	**Intermediate Microbiota**	**Parameter**	**Control**	**0.15**	**0.3**	**0.6**	**1**	**2**	**4**
Candida albicans - Escherichia coli (V118)	Abs 570 (SD)	1.27 (0.05)	1.26 (0.002)	1.21 (0.02)	1.25 (0.02)	1.19 (0.13)	1.14 (0.007)	1.13 (0.01)	*Candida albicans - Gram positive coccus* (V543)	Abs 570 (SD)	1.27 (0.05)	1.26 (0.002)	1.21 (0.02)	1.25 (0.02)	1.19 (0.13)	1.12 (0.007)	1.12 (0.01)
	Abs 630 (SD)	1.2 (0.02)	1.16 (0.02)	1.15 (0.004)	1.18 (0.03)	1.13 (0.11)	1.11 (0.03)	1.1 (0.01)		Abs 630 (SD)	1.2 (0.02)	1.18 (0.02)	1.15 (0.004)	1.16 (0.03)	1.13 (0.11)	1.08 (0.03)	1.07 (0.01)
	Mean CFU (SD)	2.37E+08 (2.82E+07)	2.37E+08 (2.82E+07)	6.95E+07 (1.18E+07)	5.36E+07 (6.38E+05)	2.59E+07 (1.75E+05)	1.87E+07 (1.65E+06)	1.82E+07 (4.13E+05)		Mean CFU (SD)	2.25E+08 (2.58E+07)	9.69E+07 (1.69E+07)	7.12E+07 (1.07E+07)	4.63E+07 (5.41E+06)	2.24E+07 (3.99E+06)	1.36E+07 (1.40E+06)	7.38E+06 (8.75E+05)
	% Inhibition (%SD)	- (11.90)	- (11.91)	70.67 (17.02)	77.38 (1.18)	89.07 (0.67)	92.11 (8.82)	92.30 (2.26)		% Inhibition (%SD)	- (11.46)	56.88 (17.40)	68.31 (15)	79.41 (11.69)	90.04 (17)	93.95 (10.31)	96.71 (11.86)
**Candidiasis**	**Parameter**	**Control**	**0.15**	**0.3**	**0.6**	**1**	**2**	**4**	**Candidiasis**	**Parameter**	**Control**	**0.15**	**0.3**	**0.6**	**1**	**2**	**4**

Candida albicans (V161)	Abs 570 (SD)	1.1 (0.03)	1.0 (0.03)	1.04 (0.03)	1.0 (0.02)	1.0 (0.01)	0.99 (0.07)	0.43 (0.04)
	Abs 630 (SD)	1 (0.03)	0.99 (0.03)	0.97 (0.03)	0.97 (0.03)	0.90 (0.02)	0.92 (0.07)	0.39 (0.04)
	Mean CFU (SD)	1.20E+08 (2.33E+07)	5.71E+07 (2.88E+05)	3.89E+07 (1.14E+07)	5.17E+07 (6.50E+05)	2.20E+07 (1.63E+05)	1.77E+07 (1.34E+06)	1.68E+07 (1.66E+06)
	% Inhibition (%SD)	- (19.45)	52.23 (0.50)	67.48 (29)	56.80 (1.25)	81.56 (0.73)	85.16 (7.54)	85.95 (9.90)
Candida albicans (V218)	Abs 570 (SD)	1.17 (0.07)	1.13 (0.1)	1.13 (0.05)	1.14 (0.09)	1.157 (0.07)	1.04 (0.1)	0.88 (0.03)
	Abs 630 (SD)	1.1 (0.06)	1.06 (0.1)	1.07 (0.05)	1.07 (0.08)	1.08 (0.06)	0.99 (0.1)	0.83 (0.03)
	Mean CFU (SD)	8.86E+07 (9.83E+06)	4.83E+07 (1.28E+06)	1.96E+07 (1.40E+06)	1.79E+07 (3.75E+04)	4.10E+07 (1.48E+06)	9.43E+06 (1.75E+05)	9.89E+06 (2.38E+05)
	% Inhibition (%SD)	- (11.08)	45.52 (2.64)	77.94 (7.16)	79.76 (0.20)	53.70 (3.59)	89.36 (1.85)	88.84 (2.40)
Candida albicans (V252)	Abs 570 (SD)	0.97 (0.16)	0.62 (0.045)	0.69 (0.043)	0.63 (0.044)	0.64 (0.045)	0.62 (0.043)	0.64 (0.001)
	Abs 630 (SD)	0.92 (0.15)	0.60 (0.041)	0.66 (0.041)	0.6 (0.042)	0.59 (0.041)	0.6 (0.020)	0.64 (0.001)
	Mean CFU (SD)	3.67E+08 (8.16E+06)	4.40E+07 (1.01E+06)	5.02E+07 (1.28E+07)	8.64E+06 (1.63E+05)	3.41E+07 (2.03E+06)	3.57E+07 (3.25E+05)	3.15E+07 (2.31E+06)
	% Inhibition (%SD)	- (2.22)	88.01 (2.30)	86.34 (25)	97.64 (1.88)	90.73 (5.94)	90.27 (0.9)	91.42 (7.34)
Candida albicans (V449)	Abs 570 (SD)	0.97 (0.16)	0.62 (0.045)	0.59 (0.043)	0.63 (0.044)	0.66 (0.045)	0.04 (0.043)	0.04 (0.001)
	Abs 630 (SD)	0.92 (0.15)	0.60 (0.041)	0.56 (0.041)	0.6 (0.042)	0.59 (0.041)	0.04 (0.020)	0.11 (0.001)
	Mean CFU (SD)	1.16E+08 (1.72E+07)	5.99E+07 (1.50E+05)	5.00E+07 (1.09E+06)	2.17E+07 (5.35E+06)	3.88E+07 (1.68E+06)	1.11E+07 (3.88E+05)	2.11E+07 (1.14E+07)
	% Inhibition (%SD)	- (14.80)	48.43 (0.25)	56.88 (2.17)	81.30 (24)	66.54 (4.31)	90.44 (3.49)	81.80 (53)

Candida albicans (V450)	Abs 570 (SD)	1.36 (0.01)	1.21 (0.001)	1.30 (0.03)	1.30 (0.02)	1.29 (0.01)	1.26 (0.01)	0.37 (0.05)
	Abs 630 (SD)	1.28 (0.01)	1.13 (0.001)	1.22 (0.03)	1.22 (0.01)	1.21 (0.01)	1.18 (0.01)	0.35 (0.05)
	Mean CFU (SD)	1.71E+08 (1.54E+07)	5.72E+07 (1.49E+07)	3.18E+07 (1.06E+07)	1.52E+07 (1.79E+06)	1.21E+07 (4.00E+05)	7.15E+06 (3.00E+05)	6.74E+06 (4.13E+05)
	% Inhibition (%SD)	- (9.01)	66.53 (23.98)	81.37 (33)	91.10 (11.76)	92.94 (3.31)	95.81 (4.19)	96.05 (6.12)
Candida albicans (V535)	Abs 570 (SD)	1.12 (0.01)	0.85 (0.05)	0.81 (0.04)	0.82 (0.04)	0.8 (0.04)	0.78 (0.04)	0.75 (0.04)
	Abs 630 (SD)	1.07 (0.009)	0.78 (0.04)	0.77 (0.045)	0.78 (0.04)	0.71 (0.04)	0.7 (0.04)	0.7 (0.04)
	Mean CFU (SD)	7.42E+07 (8.31E+06)	1.05E+07 (2.75E+05)	5.19E+06 (5.63E+05)	5.24E+06 (8.75E+04)	1.48E+06 (2.25E+05)	1.29E+06 (1.25E+04)	7.75E+05 (1.00E+05)
	% Inhibition (%SD)	- (11.20)	85 (2.62)	93 (10.8)	92 (1.67)	98 (15)	98 (0.9)	98 (12.9)
Candida albicans (V540)	Abs 570 (SD)	1.12 (0.01)	0.85 (0.05)	0.81 (0.04)	0.82 (0.04)	0.8 (0.04)	0.78 (0.04)	0.75 (0.04)
	Abs 630 (SD)	1.07 (0.009)	0.7 (0.04)	0.77 (0.045)	0.78 (0.04)	0.72 (0.04)	0.74 (0.04)	0.71 (0.04)
	Mean CFU (SD)	5.24E+07 (3.03E+06)	9.58E+06 (1.75E+05)	6.81E+06 (8.38E+05)	5.24E+06 (2.38E+05)	1.98E+06 (7.50E+04)	1.29E+06 (2.88E+05)	4.63E+05 (1.38E+05)
	% Inhibition (%SD)	- (5.77)	81.71 (1.82)	86.99 (12)	90 (4.53)	96 (3.79)	97 (22)	99.11 (29)

Mixed Infection	Parameter	Control	0.15	0.3	0.6	1	2	4
Candida albicans Candidiasis - Aerobic vaginitis (V415)	Abs 570 (SD)	1.10 (0.02)	1.08 (0.01)	1.09 (0.04)	1.09 (0.01)	1.07 (0.02)	1.09 (0.04)	1.01 (0.07)
	Abs 630 (SD)	1.04 (0.02)	1.03 (0.02)	1.0 (0.02)	1.01 (0.01)	1.02 (0.03)	0.99 (0.02)	0.96 (0.09)
	Mean CFU (SD)	3.22E+07 (4.79E+06)	2.01E+07 (1.36E+06)	1.52E+07 (3.25E+05)	1.68E+07 (2.11E+06)	8.55E+06 (1.20E+06)	6.21E+06 (1.88E+05)	2.15E+06 (4.00E+05)
	% Inhibition (%SD)	- (14.86)	37 (6.76)	52 (2.14)	47 (12.5)	73.4 (140	80 (3.01)	93.3 (18)
Candida albicans Candidiasis - Aerobic vaginitis (V527)	Abs 570 (SD)	1.23 (0.007)	1.19 (0.01)	1.15 (0.01)	1.2 (0.02)	1.15 (0.01)	1.15 (0.007)	1.12 (0.01)
	Abs 630 (SD)	1.17 (0.01)	1.15 (0.01)	1.09 (0.01)	1.19 (0.02)	1.05 (0.01)	1.09 (0.004)	1.04 (0.01)
	Mean CFU (SD)	3.94E+07 (1.50E+06)	2.01E+07 (2.00E+05)	1.43E+07 (1.50E+06)	2.48E+07 (4.60E+06)	9.43E+06 (2.25E+05)	1.67E+07 (2.30E+06)	6.35E+06 (6.50E+05)
	% Inhibition (%SD)	- (3.80)	49 (0.992	63 (10.4)	37 (18)	76.0 (2.38)	57.6 (13.7)	83.8 (10.2)

Mixed Infection	Parameter	Control	0.15	0.3	0.6	1	2	4
Candida glabrata Candidiasis - Aerobic vaginitis (V601)	Abs 570 (SD)	1.21 (0.005)	1.2 (0.02)	1.19 (0.02)	1.17 (0.02)	1.15 (0.01)	0.12 (0.02)	0.05 (0.003)
	Abs 630 (SD)	1.16 (0.008)	1.16 (0.01)	1.15 (0.02)	1.14 (0.01)	1.13 (0.01)	0.096 (0.02)	0.048 (0.005)
	Mean CFU (SD)	7.83E+07 (7.21E+06)	4.15E+07 (4.91E+06)	1.09E+07 (1.66E+06)	5.85E+06 (1.50E+05)	4.91E+06 (8.75E+04)	4.40E+06 (5.00E+04)	2.53E+06 (2.25E+05)
	% Inhibition (%SD)	- (9.20)	47 (11.8)	86 (15)	92 (2.56)	93.7 (1.78)	94.3 (1.13)	96 (8.91)

Biofilm Inhibitory concentration								
Flucytosine								
Reference control strain	Parameter	Concentration (µg/ml)						
		Control	16	32	64	128	256	512
Candida albicans ATCC 10231	Abs 570 (SD)	1.3 (0.01)	1.3 (0.05)	1.26 (0.03)	1.26 (0.01)	1.25 (0.01)	1.23 (0.09)	0.95 (0.05)
	Abs 630 (SD)	1.27 (0.010)	1.26 (0.05)	1.23 (0.03)	1.23 (0.02)	1.22 (0.01)	1.20 (0.09)	0.90 (0.06)
	Mean CFU (SD)	2.35E+08 (4.85E+07)	1.30E+08 (1.22E+07)	9.33E+07 (1.38E+07)	7.41E+07 (2.71E+07)	6.97E+07 (5.38E+05)	5.39E+07 (1.08E+07)	3.46E+07 (8.13E+05)
	% Inhibition (%SD)	- (20.66)	44.79 (9.41)	60.27 (14.78)	68.43 (36.5)	70.30 (0.77)	77.03 (20.10)	85.24 (2.34)

Healthy Microbiota	Parameter	Concentration (µg/ml)							Healthy Microbiota	Parameter	Concentration (µg/ml)						
		Control	16	32	64	128	256	512			Control	16	32	64	128	256	512
Candida albicans (V130)	Abs 570 (SD)	1.46 (0.03)	1.3 (0.004)	1.29 (0.007)	1.25 (0.02)	1.24 (0.01)	1.13 (0.01)	1.12 (0.03)	*Candida albicans* (V251)	Abs 570 (SD)	1.20 (0.01)	1.19 (0.012)	1.19 (0.01)	1.17 (0.01)	1.19 (0.01)	1 (0.005)	1.01 (0.02)
	Abs 630 (SD)	1.30 (0.03)	1.28 (0.007)	1.27 (0.005)	1.23 (0.022)	1.2 (0.01)	1.12 (0.03)	1.12 (0.03)		Abs 630 (SD)	1.15 (0.009)	1.13 (0.01)	1.14 (0.01)	1.11 (0.01)	1.13 (0.01)	1 (0.008)	1 (0.02)
	Mean CFU (SD)	1.45E+08 (4.62E+07)	5.31E+07 (1.09E+06)	2.39E+07 (2.16E+06)	2.22E+07 (3.30E+06)	1.99E+07 (2.03E+06)	1.18E+07 (5.49E+06)	1.10E+07 (5.28E+06)		Mean CFU (SD)	8.34E+07 (5.35E+06)	4.72E+07 (4.78E+06)	5.42E+07 (1.54E+07)	4.68E+07 (1.05E+07)	3.65E+07 (1.34E+06)	6.21E+06 (1.63E+05)	7.36E+06 (8.63E+05)
	% Inhibition (%SD)	- (31.39)	63.33 (2.05)	83.48 (9.04)	84.70 (14.90)	86.27 (10.19)	91.84 (46.46)	92.42 (48.06)		% Inhibition (%SD)	- (6.41)	43.38 (10.11)	34.98 (28.40)	43.93 (22.48)	56.19 (3.66)	92.55 (2.62)	91.17 (2.62)
Candida albicans (V134)	Abs 570 (SD)	1.20 (0.01)	1.17 (0.012)	1.16 (0.01)	1.15 (0.01)	1.09 (0.01)	1.09 (0.005)	1.07 (0.02)	*Candida albicans* (V448)	Abs 570 (SD)	1.10 (0.01)	0.63 (0.10)	0.55 (0.15)	0.50 (0.13)	0.74 (0.02)	0.69 (0.09)	0.69 (0.15)
	Abs 630 (SD)	1.15 (0.009)	1.11 (0.01)	1.10 (0.01)	1.09 (0.01)	1.03 (0.01)	1.03 (0.008)	1.01 (0.02)		Abs 630 (SD)	1.04 (0.01)	0.61 (0.09)	0.53 (0.14)	0.48 (0.12)	0.71 (0.01)	0.67 (0.09)	0.66 (0.14)
	Mean CFU (SD)	2.96E+08 (2.84E+07)	6.56E+07 (1.54E+06)	5.79E+07 (1.34E+06)	5.22E+07 (1.56E+06)	2.49E+07 (6.50E+05)	1.98E+07 (1.16E+06)	1.45E+07 (3.49E+06)		Mean CFU (SD)	1.33E+08 (1.73E+07)	5.12E+07 (8.16E+06)	5.81E+07 (4.14E+06)	4.68E+07 (1.46E+06)	3.90E+07 (2.79E+06)	1.78E+07 (2.73E+06)	1.48E+07 (1.39E+06)
	% Inhibition (%SD)	- (9.59)	77.85 (2.34)	80.46 (2.31)	82.39 (2.99)	91.61 (2.62)	93.32 (5.87)	95.10 (24.03)		% Inhibition (%SD)	- (13.00)	61.57 (15.95)	56.37 (7.12)	64.87 (3.13)	70.71 (7.15)	86.65 (15.33)	88.90 (9.38)
Candida albicans (V196)	Abs 570 (SD)	1.21 (0.05)	1.15 (0.02)	1.17 (0.02)	1.17 (0.03)	1.19 (0.02)	1.02 (0.11)	1.02 (0.05)	*Candida albicans* (V451)	Abs 570 (SD)	1.19 (0.02)	1.10 (0.03)	1.10 (0.04)	1.04 (0.03)	1.1 (0.03)	1.01 (0.02)	1 (0.01)
	Abs 630 (SD)	1.23 (0.05)	1.07 (0.01)	1.10 (0.02)	1.09 (0.03)	1.1 (0.02)	1.02 (0.10)	1.01 (0.05)		Abs 630 (SD)	1.13 (0.02)	1.04 (0.03)	1.04 (0.04)	1.03 (0.03)	1.03 (0.02)	1.01 (0.02)	0.96 (0.01)
	Mean CFU (SD)	1.62E+08	6.51E+07	5.65E+07	5.05E+07+	1.82E+07 (1.04E+06)	1.40E+07	7.74E+06		Mean CFU (SD)	2.30E+08 (1.84E+07)	7.25E+07	6.00E+07	5.41E+07	4.79E+07	1.58E+07	8.71E+06 (1.56E+06)

		(2.80E+07)	(1.60E+06)	(1.64E+06)	(5.25E+05)		(2.50E+05)	(1.38E+05)				(3.58E+06)	(9.75E+05)	(1.14E+06)	(1.65E+06)	(2.75E+06)	
	% Inhibition (%SD)	- (17.32)	59.79 (2.46)	65.08 (2.90)	68.83 (1.04)	88.77 (5.70)	91.35 (1.79)	95.22 (1.78)		% Inhibition (%SD)	- (7.98)	68.47 (4.93)	73.90 (1.62)	76.49 (2.10)	79.16 (3.44)	93.15 (17.46)	96.21 (17.93)
Candida glabrata (V197)	Abs 570 (SD)	1.10 (0.01)	0.63 (0.10)	0.55 (0.15)	0.50 (0.13)	0.74 (0.02)	0.69 (0.09)	0.69 (0.15)	*Candida albicans* (V580)	Abs 570 (SD)	1.29 (0.01)	1.18 (0.02)	1.23 (0.009)	1.24 (0.012)	1.25 (0.02)	1.16 (0.006)	1.16 (0.01)
	Abs 630 (SD)	1.04 (0.01)	0.61 (0.09)	0.53 (0.14)	0.48 (0.12)	0.71 (0.01)	0.67 (0.09)	0.66 (0.14)		Abs 630 (SD)	1.24 (0.01)	1.2 (0.02)	1.13 (0.04)	1.17 (0.004)	1.18 (0.01)	1 (0.02)	1.1 (0.01)
	Mean CFU (SD)	1.77E+08 (8.53E+06)	4.57E+07 (4.73E+06)	2.67E+07 (1.25E+07)	1.32E+07 (3.63E+05)	1.27E+07 (4.88E+05)	9.60E+06 (4.75E+05)	7.46E+06 (1.71E+06)		Mean CFU (SD)	4.54E+07 (6.63E+06)	1.25E+07 (3.68E+06)	7.61E+06 (1.38E+05)	1.00E+07 (1.86E+06)	1.14E+07 (2.81E+06)	4.50E+06 (2.50E+05)	7.10E+06 (2.35E+06)
	% Inhibition (%SD)	- (4.82)	74.22 (10.35)	84.93 (46.77)	92.52 (2.74)	92.83 (3.84)	94.58 (4.95)	95.79 (22.95)		% Inhibition (%SD)	- (14.60)	72.45 (29.40)	83.22 (1.81)	77.88 (18.56)	74.85 (24.64)	90.08 (5.56)	84.35 (33.10)
Candida albicans (V202)	Abs 570 (SD)	1.19 (0.02)	1.10 (0.03)	1.10 (0.04)	1.04 (0.03)	1.1 (0.03)	1.1 (0.02)	1.1 (0.01)									
	Abs 630 (SD)	1.13 (0.02)	1.04 (0.03)	1.04 (0.04)	0.98 (0.03)	1.05 (0.02)	1.05 (0.02)	1.04 (0.01)									
	Mean CFU (SD)	1.46E+08 (7.85E+06)	3.32E+07 (9.03E+06)	2.16E+07 (1.64E+06)	1.78E+07 (1.24E+05)	1.43E+07 (2.88E+05)	1.09E+07 (1.53E+06)	1.10E+07 (2.75E+05)									
	% Inhibition (%SD)	- (5.37)	77.29 (27.20)	85.24 (7.59)	87.83 (0.70)	90.24 (2.02)	92.54 (13.99)	92.45 (2.49)									
**Intermediate Microbiota**	**Parameter**	**Control**	**16**	**32**	**64**	**128**	**256**	**512**	**Intermediate Microbiota**	**Parameter**	**Control**	**16**	**32**	**64**	**128**	**256**	**512**
Candida albicans - Escherichia coli (V118)	Abs 570 (SD)	1.33 (0.04)	1.23 (0.04)	1.17 (0.07)	1.20 (0.1)	0.14 (0.04)	1.10 (0.16)	0.93 (0.5)	*Candida albicans - Gram positive coccus* (V543)	Abs 570 (SD)	1.33 (0.04)	1.23 (0.04)	1.17 (0.07)	1.20 (0.1)	0.94 (0.144	0.92 (0.16)	0.91 (0.5)
	Abs 630 (SD)	1.26 (0.02)	1.17 (0.03)	1.11 (0.04)	1.1 (0.09)	1.08 (0.13)	1.03 (0.13)	0.87 (0.4)		Abs 630 (SD)	1.26 (0.02)	1.17 (0.03)	1.11 (0.04)	1.1 (0.09)	1.08 (0.13)	1.03 (0.13)	0.87 (0.4)
	Mean CFU (SD)	2.37E+08 (2.82E+07)	7.32E+07 (7.53E+06)	4.87E+07 (1.49E+06)	4.58E+07 (1.77E+06)	2.48E+07 (6.39E+06)	2.28E+07 (3.04E+06)	1.91E+07 (3.11E+06)		Mean CFU (SD)	2.25E+08 (2.58E+07)	1.10E+08 (1.55E+07)	7.28E+07 (6.95E+06)	4.78E+07 (9.55E+06)	1.99E+07 (1.93E+06)	1.82E+07 (3.30E+06)	1.14E+07 (8.63E+05)
	% Inhibition (%SD)	- (11.91)	69.12 (10.28)	79.47 (3.06)	80.69 (3.86)	89.55 (25.77)	90.40 (13.34)	91.94 (16.29)		% Inhibition (%SD)	- (11.47)	50.93 (14.08)	67.60 (9.55)	78.75 (19.99)	91.16 (9.69)	91.90 (18.13)	94.93 (7.57)
**Candidiasis**	**Parameter**	**Control**	**16**	**32**	**64**	**128**	**256**	**512**	**Candidiasis**	**Parameter**	**Control**	**16**	**32**	**64**	**128**	**256**	**512**
	Abs 570 (SD)	0.83 (0.04)	0.76 (0.04)	0.78 (0.04)	0.8 (0.04)	0.8 (0.04)	0.7 (0.04)	0.40 (0.03)		Abs 570 (SD)	1.52 (0.1)	1.3 (0.1)	1.32 (0.05)	1.29 (0.04)	1.2 (0.08)	1.23 (0.03)	1.21 (0.07)

Candida albicans (V161)	Abs 630 (SD)	0.80 (0.03)	0.68 (0.03)	0.7 (0.04)	0.7 (0.03)	0.7 (0.04)	0.7 (0.04)	0.37 (0.03)
	Mean CFU (SD)	1.20E+08 (2.33E+07)	6.22E+07 (1.48E+07)	2.43E+07 (1.73E+06)	2.45E+07 (1.03E+06)	3.15E+07 (1.08E+06)	3.27E+07 (5.76E+06)	1.72E+07 (3.96E+06)
	% Inhibition (%SD)	- (19.46)	47.97 (23.85)	79.65 (7.09)	79.53 (4.19)	73.67 (3.42)	72.66 (17.63)	85.58 (22.99)
Candida albicans (V218)	Abs 570 (SD)	1.28 (0.01)	1.19 (0.06)	1.21 (0.10)	1.20 (0.09)	1.25 (0.07)	1.19 (0.01)	1.12 (0.01)
	Abs 630 (SD)	1.16 (0.08)	1.12 (0.05)	1.1 (0.02)	1.1 (0.07)	1.1 (0.02)	1.1 (0.01)	1.1 (0.006)
	Mean CFU (SD)	8.86E+07 (9.83E+06)	5.38E+07 (2.80E+06)	5.50E+07 (2.66E+06)	1.83E+07 (1.80E+06)	1.43E+07 (1.13E+05)	1.34E+07 (2.83E+06)	6.24E+06 (1.14E+06)
	% Inhibition (%SD)	- (11.09)	39.35 (5.21)	37.90 (4.84)	79.41 (9.86)	83.88 (0.79)	84.85 (21.04)	92.96 (18.24)
Candida albicans (V252)	Abs 570 (SD)	0.95 (0.18)	0.83 (0.045)	0.91 (0.043)	0.63 (0.046)	0.63 (0.045)	0.06 (0.044)	0.04 (0.002)
	Abs 630 (SD)	0.91 (0.16)	0.8 (0.02)	0.88 (0.02)	0.60 (0.02)	0.6 (0.02)	0.06 (0.02)	0.04 (0.002)
	Mean CFU (SD)	3.67E+08 (8.16E+06)	1.01E+08 (2.39E+07)	1.87E+08 (4.00E+07)	2.40E+08 (2.65E+07)	1.83E+07 (2.31E+06)	5.50E+06 (5.00E+04)	4.33E+06 (1.00E+05)
	% Inhibition (%SD)	- (2.22)	72.53 (23.69)	49.04 (21.35)	34.57 (11.02)	95.01 (12.61)	98.50 (0.91)	98.82 (2.31)
Candida albicans (V449)	Abs 570 (SD)	0.95 (0.18)	0.63 (0.045)	0.63 (0.043)	0.63 (0.046)	0.63 (0.045)	0.62 (0.044)	0.04 (0.002)
	Abs 630 (SD)	0.91 (0.16)	0.6 (0.02)	0.61 (0.02)	0.60 (0.02)	0.6 (0.02)	0.6 (0.02)	0.04 (0.002)
	Mean CFU (SD)	1.16E+08 (1.72E+07)	8.61E+07 (1.14E+06)	5.53E+07 (1.43E+06)	4.23E+07 (5.26E+06)	1.65E+07 (2.75E+06)	1.48E+07 (5.50E+05)	9.71E+06 (1.01E+06)
	% Inhibition (%SD)	- (14.81)	25.83 (1.32)	52.37 (2.58)	63.59 (12.45)	85.81 (16.69)	87.23 (3.71)	91.63 (10.42)
**Mixed Infection**	**Parameter**	**Control**	**16**	**32**	**64**	**128**	**256**	**512**

Candida albicans (V450)	Abs 630 (SD)	1.44 (0.08)	1.2 (0.1)	1.2 (0.1)	1.21 (0.04)	1.15 (0.07)	1.19 (0.04)	1.17 (0.08)
	Mean CFU (SD)	1.71E+08 (1.54E+07)	1.14E+08 (9.51E+06)	9.58E+07 (2.35E+07)	2.30E+07 (2.70E+06)	7.33E+06 (2.25E+05)	1.44E+07 (2.99E+06)	1.11E+07 (1.59E+06)
	% Inhibition (%SD)	- (9.02)	33.50 (8.38)	43.92 (24.52)	86.57 (11.77)	95.71 (3.07)	91.59 (20.80)	93.49 (14.29)
Candida albicans (V535)	Abs 570 (SD)	1.10 (0.05)	0.86 (0.05)	0.86 (0.04)	0.88 (0.05)	0.84 (0.04)	0.83 (0.04)	0.77 (0.04)
	Abs 630 (SD)	1.05 (0.04)	0.82 (0.048)	0.82 (0.04)	0.84 (0.048)	0.83 (0.04)	0.79 (0.04)	0.73 (0.04)
	Mean CFU (SD)	7.42E+07 (8.31E+06)	1.64E+07 (1.44E+06)	1.24E+07 (9.38E+05)	9.95E+06 (3.50E+05)	7.09E+06 (2.13E+05)	6.43E+06 (7.50E+04)	5.09E+06 (2.88E+05)
	% Inhibition (%SD)	- (11.20)	77.84 (8.75)	83.27 (7.55)	86.59 (3.52)	90.45 (3.00)	91.34 (1.17)	93.14 (5.65)
Candida albicans (V540)	Abs 570 (SD)	1.10 (0.05)	0.86 (0.05)	0.86 (0.04)	0.88 (0.05)	0.8 (0.04)	0.83 (0.04)	0.77 (0.04)
	Abs 630 (SD)	1.05 (0.04)	0.82 (0.048)	0.82 (0.04)	0.84 (0.048)	0.83 (0.04)	0.79 (0.04)	0.73 (0.04)
	Mean CFU (SD)	5.24E+07 (3.03E+06)	1.24E+07 (6.75E+05)	8.98E+06 (8.50E+05)	7.90E+06 (1.00E+05)	7.86E+06 (4.13E+05)	6.96E+06 (2.88E+05)	4.53E+06 (2.75E+05)
	% Inhibition (%SD)	- (5.78)	76.28 (5.43)	82.86 (9.47)	84.92 (1.27)	84.99 (5.25)	86.71 (4.13)	91.36 (6.08)
**Mixed Infection**	**Parameter**	**Control**	**16**	**32**	**64**	**128**	**256**	**512**

Candida albicans Candidiasis - Aerobic vaginitis (V415)	Abs 570 (SD)	1.18 (0.02)	1.07 (0.01)	1.07 (0.04)	1.00 (0.04)	1.08 (0.03)	1.08 (0.03)	1.07 (0.04)
	Abs 630 (SD)	1.12 (0.02)	1.02 (0.02)	1.02 (0.03)	0.95 (0.03)	1.03 (0.02)	1.03 (0.02)	1.02 (0.02)
	Mean CFU (SD)	3.22E+07 (4.79E+06)	2.20E+07 (1.66E+06)	1.38E+07 (1.91E+06)	9.45E+06 (1.20E+06)	7.46E+06 (1.63E+05)	5.39E+06 (8.63E+05)	5.05E+06 (2.75E+05)
	% Inhibition (%SD)	- (14.86)	31.59 (7.54))	57.28 (13.90)	70.66 (12.70)	76.83 (2.18)	83.28 (16.01)	84.32 (5.45)
Candida albicans Candidiasis - Aerobic vaginitis (V527)	Abs 570 (SD)	1.26 (0.01)	1.18 (0.006)	1.17 (0.02)	1.17 (0.01)	1.15 (0.008)	1.16 (0.01)	1.13 (0.004)
	Abs 630 (SD)	1.20 (0.01)	1.13 (0.006)	1.12 (0.02)	1.12 (0.01)	1.1 (0.008)	1.1 (0.01)	1.1 (0.003)
	Mean CFU (SD)	3.94E+07 (1.50E+06)	1.64E+07 (2.53E+06)	1.43E+07 (2.75E+05)	1.29E+07 (1.13E+06)	1.00E+07 (1.53E+06)	1.07E+07 (1.90E+06)	7.49E+06 (4.88E+05)
	% Inhibition (%SD)	- (3.80)	58.34 (15.37)	63.79 (1.93)	67.34 (8.74)	74.57 (15.21)	72.86 (17.76)	81.01 (6.51)

Candida glabrata Candidiasis - Aerobic vaginitis (V601)	Abs 570 (SD)	1.30 (0.01)	1.25 (0.02)	1.28 (0.01)	1.23 (0.01)	1.22 (0.02)	1.07 (0.02)	0.13 (0.01)
	Abs 630 (SD)	1.24 (0.06)	1.19 (0.02)	1.22 (0.01)	1.22 (0.01)	1.17 (0.02)	1.01 (0.01)	0.10 (0.008)
	Mean CFU (SD)	7.83E+07 (7.21E+06)	2.09E+07 (1.53E+06)	1.73E+07 (1.53E+06)	1.39E+07 (1.38E+05)	8.03E+06 (2.75E+05)	4.45E+06 (1.50E+05)	2.11E+06 (3.63E+05)
	% Inhibition (%SD)	- (9.21)	73.29 (7.29)	77.95 (8.83)	82.24 (0.99)	89.76 (3.43)	94.32 (3.37)	97.30 (17.16)

Summary table with the absorbance values at 570 and 630 nm, CFU, percentage of inhibition and their respective standard deviation values obtained in Biofilm tests carried out on 21 samples of different medical diagnoses plus a *Candida albicans* ATCC control strain, carried out with 3 commercial antifungals of different chemical families (1. Fluconazole, 2. Micafungin, 3. Flucytosin).

Supplementary Table S6. Overall results of biofilm eradication assays by biomass and CFU counting methodologies using fluconazole, micafungin, and flucytosine.

Eradication								
Fluconazole								
Reference control strain	Parameter	Concentration (µg/ml)						
		Control	4	8	16	32	64	128
Candida albicans ATCC 10231	Abs 570 (SD)	1.34 (0.02)	1.27 (0.03)	1.25 (0.02)	1.00 (0.05)	0.80 (0.06)	0.60 (0.05)	0.34 (0.03)
	Abs 630 (SD)	1.27 (0.02)	1.23 (0.03)	1.22 (0.02)	0.97 (0.05)	0.78 (0.06)	0.58 (0.05)	0.31 (0.03)
	Mean CFU (SD)	3.33E+08 (1.45E+07)	2.27E+08 (2.24E+07)	1.46E+08 (3.35E+07)	9.31E+07 (1.16E+06)	7.32E+07 (1.08E+07)	5.32E+07 (2.63E+06)	1.82E+07 (1.61E+06)
	% Eradication (%SD)	- (4.35)	31.88 (9.85)	56.12 (22.89)	72.05 (1.25)	78.04 (14.74)	84.04 (4.94)	94.53 (8.85)

Healthy Microbiota	Parameter	Concentration (µg/ml)						
		Control	4	8	16	32	64	128
Candida albicans (V130)	Abs 570 (SD)	1.24 (0.013)	1.22 (0.008)	1.14 (0.02)	1.21 (0.006)	1.18 (0.015)	1.17 (0.02)	1.12 (0.02)
	Abs 630 (SD)	1.18 (0.012)	1.16 (0.009)	1.09 (0.020)	1.16 (0.007)	1.13 (0.018)	1.12 (0.017)	1.07 (0.02)
	Mean CFU (SD)	1.46E+08 (4.52E+07)	6.16E+07 (1.76E+07)	4.34E+07 (3.39E+06)	1.67E+07 (7.25E+05)	1.86E+07 (5.23E+06)	2.20E+07 (2.20E+06)	1.44E+07 (4.86E+06)
	% Eradication (%SD)	- (30.89)	57.86 (28.50)	70.33 (7.81)	88.58 (4.34)	87.31 (28.17)	84.95 (10.00)	90.14 (33.74)
Candida albicans (V134)	Abs 570 (SD)	1.19 (0.01)	1.11 (0.02)	1.1 (0.03)	1.08 (0.003)	0.89 (0.02)	0.67 (0.005)	0.53 (0.003)
	Abs 630 (SD)	1.12 (0.01)	1.06 (0.02)	1 (0.03)	0.97 (0.003)	0.84 (0.01)	0.64 (0.004)	0.49 (0.004)
	Mean CFU (SD)	2.96E+08 (2.84E+07)	3.12E+07 (1.41E+06)	2.60E+07 (1.00E+05)	1.89E+07 (1.50E+05)	1.19E+07 (8.75E+04)	6.15E+06 (4.75E+05)	3.13E+06 (1.00E+05)
	% Eradication (%SD)	- (9.59)	89.47 (4.53)	91.24 (0.39)	93.62 (0.79)	95.98 (0.73)	97.92 (7.72)	98.95 (3.20)
Candida albicans (V196)	Abs 570 (SD)	1.21 (0.08)	1.17 (0.009)	1.15 (0.02)	0.8 (0.43)	0.78 (0.40)	0.74 (0.07)	0.71 (0.03)
	Abs 630 (SD)	1.13 (0.07)	1.09 (0.008)	1.07 (0.02)	0.77 (0.40)	0.74 (0.38)	0.71 (0.06)	0.69 (0.06)

Healthy Microbiota	Parameter	Concentration (µg/ml)						
		Control	4	8	16	32	64	128
Candida albicans (V251)	Abs 570 (SD)	1.24 (0.03)	1.23 (0.01)	1.25 (0.02)	1.22 (0.01)	1.19 (0.01)	1.12 (0.02)	1.1 (0.02)
	Abs 630 (SD)	1.19 (0.02)	1.18 (0.01)	1.20 (0.02)	1.17 (0.01)	1.14 (0.02)	1.1 (0.02)	1.07 (0.02)
	Mean CFU (SD)	8.34E+07 (5.35E+06)	5.29E+07 (2.86E+06)	1.88E+07 (2.34E+06)	1.52E+07 (2.88E+05)	1.07E+07 (1.50E+05)	6.76E+06 (2.88E+05)	5.71E+06 (1.63E+05)
	% Eradication (%SD)	- (6.41)	36.62 (5.41)	77.47 (12.44)	81.73 (1.89)	87.17 (1.40)	91.89 (4.25)	93.15 (2.84)
Candida albicans (V448)	Abs 570 (SD)	1.18 (0.01)	1.11 (0.02)	1.15 (0.03)	1.08 (0.002)	0.87 (0.02)	0.67 (0.004)	0.51 (0.003)
	Abs 630 (SD)	1.13 (0.01)	1.06 (0.02)	1.09 (0.03)	1.03 (0.003)	0.82 (0.01)	0.64 (0.004)	0.48 (0.004)
	Mean CFU (SD)	1.35E+08 (1.84E+07)	8.74E+07 (2.75E+05)	5.28E+07 (1.21E+06)	4.99E+07 (5.10E+06)	2.64E+07 (1.00E+05)	1.99E+07 (3.00E+05)	1.01E+07 (5.91E+06)
	% Eradication (%SD)	- (13.70)	35.03 (0.31)	60.75 (2.30)	62.92 (10.22)	80.36 (0.38)	85.21 (1.51)	92.50 (58.61)
Candida albicans (V451)	Abs 570 (SD)	1.21 (0.07)	1.16 (0.007)	1.18 (0.02)	1.12 (0.33)	0.97 (0.30)	0.89 (0.05)	0.85 (0.01)
	Abs 630 (SD)	1.13 (0.06)	1.1 (0.006)	1.1 (0.02)	1.09 (0.30)	0.96 (0.26)	0.82 (0.04)	0.8 (0.04)

	Mean CFU (SD)	1.62E+08 (2.80E+07)	2.36E+07 (1.59E+06)	1.92E+07 (1.38E+05)	1.32E+07 (1.38E+05)	9.94E+06 (3.13E+05)	5.68E+06 (2.75E+05)	3.59E+06 (1.13E+05)		Mean CFU (SD)	2.30E+08 (1.84E+07)	1.31E+08 (1.40E+06)	9.57E+07 (1.34E+06)	6.39E+07 (2.39E+06)	5.35E+07 (1.81E+06)	1.72E+07 (9.38E+05)	1.07E+07 (6.50E+05)
	% Eradication (%SD)	- (17.32)	85.42 (6.72)	88.16 (0.72)	91.86 (1.04)	93.86 (3.14)	96.50 (4.85)	97.78 (3.14)		% Eradication (%SD)	- (7.98)	42.90 (1.07)	58.38 (1.40)	72.22 (3.74)	76.72 (3.39)	92.53 (5.45)	95.37 (6.10)
Candida glabrata (V197)	Abs 570 (SD)	1.09 (0.02)	0.83 (0.08)	0.73 (0.03)	0.59 (0.16)	0.52 (0.07)	0.5 (0.15)	0.47 (0.008)	*Candida albicans* (V580)	Abs 570 (SD)	1.18 (0.02)	1.11 (0.03)	1.13 (0.02)	1.03 (0.06)	1.03 (0.10)	1.02 (0.06)	0.98 (0.07)
	Abs 630 (SD)	1 (0.02)	0.8 (0.08)	0.7 (0.03)	0.57 (0.16)	0.52 (0.07)	0.51 (0.15)	0.46 (0.008)		Abs 630 (SD)	1.11 (0.02)	1.06 (0.03)	1.08 (0.02)	1.05 (0.05)	0.98 (0.10)	0.97 (0.05)	0.93 (0.07)
	Mean CFU (SD)	1.80E+08 (1.14E+07)	7.26E+07 (1.31E+07)	6.63E+07 (1.07E+07)	3.38E+07 (1.48E+07)	1.89E+07 (1.55E+06)	1.36E+07 (6.25E+05)	7.54E+06 (1.51E+06)		Mean CFU (SD)	1.58E+08 (5.88E+06)	3.23E+07 (1.45E+06)	4.98E+07 (2.25E+05)	4.74E+07 (1.99E+06)	4.19E+07 (3.94E+06)	1.80E+07 (2.68E+06)	8.64E+06 (8.63E+05)
	% Eradication (%SD)	- (6.31)	59.63 (17.99)	63.16 (16.10)	81.24 (43.80)	89.52 (8.22)	92.43 (4.59)	95.81 (20.07)		% Eradication (%SD)	- (3.72)	79.54 (4.49)	68.47 (0.45)	69.97 (4.19)	73.44 (9.39)	88.61 (14.88)	94.53 (9.99)
Candida albicans (V202)	Abs 570 (SD)	1.14 (0.03)	1.11 (0.008)	1.1 (0.02)	1 (0.01)	1.09 (0.04)	0.98 (0.03)	0.95 (0.02)									
	Abs 630 (SD)	1.09 (0.03)	1.06 (0.008)	1.07 (0.02)	1.02 (0.01)	1 (0.03)	0.92 (0.03)	0.89 (0.02)									
	Mean CFU (SD)	2.44E+08 (2.78E+07)	4.67E+07 (1.19E+06)	1.86E+07 (3.13E+05)	1.28E+07 (2.63E+05)	9.40E+06 (2.75E+05)	5.79E+06 (1.88E+05)	5.11E+06 (1.88E+05)									
	% Eradication (%SD)	- (11.39)	80.86 (2.54)	82.37 (1.68)	94.76 (2.05)	96.15 (2.93)	97.63 (3.24)	97.90 (3.67)									
**Intermediate Microbiota**	**Parameter**	**Control**	**4**	**8**	**16**	**32**	**64**	**128**	**Intermediate Microbiota**	**Parameter**	**Control**	**4**	**8**	**16**	**32**	**64**	**128**
Candida albicans - Escherichia coli (V118)	Abs 570 (SD)	1.23 (0.04)	1.21 (0.01)	1.2 (0.02)	1.04 (0.007)	0.24 (0.02)	0.2 (0.02)	0.12 (0.04)	*Candida albicans - Gram positive coccus* (V543)	Abs 570 (SD)	1.26 (0.02)	1.22 (0.01)	1.25 (0.01)	1.19 (0.04)	1.06 (0.03)	1 (0.05)	0.96 (0.02)
	Abs 630 (SD)	1.17 (0.04)	1.16 (0.01)	1.16 (0.02)	0.98 (0.008)	0.21 (0.02)	0.18 (0.02)	0.1 (0.04)		Abs 630 (SD)	1.18 (0.02)	1.15 (0.01)	1.15 (0.009)	1.12 (0.01)	1.04 (0.01)	0.98 (0.04)	0.92 (0.01)
	Mean CFU (SD)	2.37E+08 (2.82E+07)	3.45E+07 (7.88E+05)	2.42E+07 (1.46E+06)	9.41E+06 (5.88E+05)	4.65E+06 (1.00E+05)	1.94E+06 (1.13E+05)	6.63E+05 (1.13E+05)		Mean CFU (SD)	2.25E+08 (2.58E+07)	5.85E+07 (2.28E+06)	2.79E+07 (1.06E+06)	2.97E+07 (1.08E+06)	1.29E+07 (7.38E+05)	5.74E+06 (1.13E+05)	3.28E+06 (1.50E+05)
	% Eradication (%SD)	- (11.91)	85.44 (2.28)	89.81 (6.05)	96.03 (6.24)	98.04 (2.15)	99.18 (5.81)	99.72 (16.98)		% Eradication (%SD)	- (11.47)	73.96 (3.89)	87.57 (3.80)	86.79 (3.62)	94.24 (5.70)	97.45 (1.96)	98.54 (4.58)
**Candidiasis**	**Parameter**	**Control**	**4**	**8**	**16**	**32**	**64**	**128**	**Candidiasis**	**Parameter**	**Control**	**4**	**8**	**16**	**32**	**64**	**128**

Candida albicans (V161)	Abs 570 (SD)	1.07 (0.09)	1.07 (0.02)	0.99 (0.004)	0.97 (0.01)	0.96 (0.04)	0.98 (0.01)	0.94 (0.01)
	Abs 630 (SD)	0.95 (0.01)	0.94 (0.02)	0.93 (0.006)	0.90 (0.01)	0.92 (0.03)	0.91 (0.01)	0.9 (0.008)
	Mean CFU (SD)	1.20E+08 (2.33E+07)	1.14E+08 (1.13E+05)	8.70E+07 (9.88E+05)	5.60E+07 (9.88E+05)	6.76E+07 (8.79E+06)	5.49E+07 (2.16E+06)	5.02E+07 (2.63E+05)
	% Eradication (%SD)	- (19.46)	4.93 (0.09)	27.20 (1.13)	53.19 (1.76)	43.43 (12.99)	54.07 (3.93)	58 (0.52)
Candida albicans (V218)	Abs 570 (SD)	1.19 (0.02)	1.17 (0.009)	1.16 (0.007)	1.16 (0.01)	1.1 (0.01)	1.1 (0.01)	1.1 (0.02)
	Abs 630 (SD)	1.11 (0.03)	1.10 (0.01)	1.1 (0.01)	1.08 (0.01)	1.02 (0.02)	1.01 (0.01)	1.01 (0.03)
	Mean CFU (SD)	1.53E+08 (1.44E+07)	7.03E+07 (1.30E+07)	5.74E+07 (1.30E+06)	5.45E+07 (2.13E+05)	4.91E+07 (1.44E+06)	2.09E+07 (6.13E+05)	1.87E+07 (2.00E+05)
	% Eradication (%SD)	- (9.41)	54.15 (18.46)	62.58 (2.26)	64.41 (0.38)	67.95 (2.92)	86.35 (2.92)	87.83 (1.07)
Candida albicans (V252)	Abs 570 (SD)	0.97 (0.23)	0.94 (0.16)	0.92 (0.20)	0.91 (0.18)	0.9 (0.19)	0.65 (0.21)	0.57 (0.21)
	Abs 630 (SD)	0.93 (0.14)	0.90 (0.21)	0.89 (0.19)	0.88 (0.17)	0.85 (0.17)	0.61 (0.20)	0.53 (0.19)
	Mean CFU (SD)	3.67E+08 (8.16E+06)	2.07E+08 (1.14E+06)	1.94E+08 (1.24E+07)	8.12E+07 (1.40E+07)	6.73E+07 (3.86E+06)	2.29E+07 (2.36E+06)	1.33E+06 (1.11E+06)
	% Eradication (%SD)	- (2.22)	43.7 (0.55)	47.18 (6.36)	77.89 (17.27)	81.67 (5.73)	93.77 (10.32)	96.37 (8.34)
Candida albicans (V449)	Abs 570 (SD)	1.17 (0.07)	1.15 (0.17)	1.13 (0.15)	1.03 (0.18)	1.03 (0.20)	1.01 (0.20)	0.94 (0.20)
	Abs 630 (SD)	1.13 (0.07)	1.12 (0.16)	1.1 (0.14)	0.99 (0.17)	0.99 (0.17)	0.92 (0.20)	0.91 (0.19)
	Mean CFU (SD)	1.16E+08 (1.72E+07)	1.04E+08 (1.14E+07)	8.52E+07 (1.34E+06)	2.83E+07 (1.14E+06)	2.37E+07 (2.38E+05)	1.87E+07 (1.55E+06)	1.26E+07 (1.36E+06)
	% Eradication (%SD)	- (14.80)	10.76 (11)	26.58 (1.56)	75.58 (4)	79.59 (1)	83.91 (8.29)	89.13 (10.80)
Candida albicans (V450)	Abs 570 (SD)	1.35 (0.02)	0.27 (0.01)	0.26 (0.01)	0.21 (0.02)	0.21 (0.02)	0.22 (0.05)	0.16 (0.06)
	Abs 630 (SD)	1.27 (0.02)	0.20 (0.02)	0.19 (0.01)	0.14 (0.02)	0.14 (0.02)	0.14 (0.05)	0.09 (0.06)
	Mean CFU (SD)	2.44E+09 (6.30E+07)	5.11E+07 (9.88E+05)	4.55E+07 (1.38E+05)	4.80E+07 (1.01E+06)	1.34E+07 (1.39E+06)	1.48E+07 (1.35E+06)	1.04E+07 (2.75E+05)
	% Eradication (%SD)	- (2.58)	97.90 (1.93)	98.13 (0.30)	98.03 (2.10)	99.45 (10.33)	99.39 (9.10)	99.58 (2.65)
Candida albicans (V535)	Abs 570 (SD)	1.22 (0.02)	1.22 (0.009)	1.2 (0.007)	1.17 (0.01)	1.16 (0.01)	1.15 (0.01)	1.13 (0.02)
	Abs 630 (SD)	1.14 (0.03)	1.14 (0.01)	1.10 (0.01)	1.09 (0.01)	1.07 (0.02)	1.05 (0.01)	1.02 (0.03)
	Mean CFU (SD)	7.42E+07 (8.31E+06)	7.44E+07 (1.11E+06)	4.61E+07 (1.15E+06)	1.62E+07 (1.38E+05)	1.38E+07 (1.38E+06)	9.38E+06 (1.00E+05)	5.99E+06 (1.13E+05)
	% Eradication (%SD)	- (11.20)	- (1.50)	37.89 (2.50)	78.21 (0.85)	81.47 (10.00)	87.36 (1.07)	91.93 (1.88)
Candida albicans (V540)	Abs 570 (SD)	1.14 (0.03)	1.1 (0.01)	1.08 (0.01)	1.07 (0.01)	1.05 (0.04)	1 (0.03)	0.96 (0.02)
	Abs 630 (SD)	1.06 (0.03)	1.02 (0.01)	1.02 (0.01)	1.01 (0.01)	1 (0.04)	0.98 (0.04)	0.91 (0.02)
	Mean CFU (SD)	5.24E+07 (3.03E+06)	1.96E+07 (1.140E+06)	1.48E+07 (2.75E+05)	1.28E+07 (1.45E+06)	1.03E+07 (1.50E+05)	6.81E+06 (1.13E+05)	3.61E+06 (1.38E+05)
	% Eradication (%SD)	- (5.78)	62.55 (5.80)	71.79 (1.86)	75.66 (11.37)	80.38 (1.46)	86.99 (1.65)	93.10 (3.81)

Mixed Infection	Parameter	Control	4	8	16	32	64	128
Candida albicans Candidiasis - Aerobic vaginitis (V415)	Abs 570 (SD)	1.73 (0.028)	1.71 (0.03)	1.67 (0.08)	1.68 (0.12)	1.48 (0.06)	1.48 (0.06)	1.24 (0.09)
	Abs 630 (SD)	1.67 (0.04)	1.66 (0.03)	1.53 (0.08)	1.59 (0.12)	1.41 (0.06)	1.4 (0.06)	1.18 (0.09)
	Mean CFU (SD)	5.95E+07 (5.73E+06)	5.83E+07 (1.11E+07)	2.37E+07 (1.50E+05)	2.79E+07 (1.53E+07)	1.40E+07 (2.63E+05)	1.60E+07 (1.08E+06)	5.23E+06 (1.48E+06)
	% Eradication (%SD)	- (9.62)	1.95 (19)	60 (0.63)	53.17 (54.84)	76.48 (1.87)	73.09 (6.71)	91.21 (28.22)
Candida albicans Candidiasis - Aerobic vaginitis (V527)	Abs 570 (SD)	1.55 (0.20)	1.39 (0.17)	1.29 (0.12)	1.13 (0.17)	1.09 (0.15)	0.95 (0.10)	0.85 (0.05)
	Abs 630 (SD)	1.49 (0.15)	1.31 (0.17)	1.22 (0.12)	1.15 (0.17)	1.01 (0.14)	0.97 (0.14)	0.88 (0.05)
	Mean CFU (SD)	1.04E+08 (1.35E+07)	4.68E+07 (2.39E+06)	1.38E+07 (2.25E+05)	1.02E+07 (8.50E+05)	7.74E+06 (6.88E+05)	6.09E+06 (4.88E+05)	4.95E+06 (7.50E+04)
	% Eradication (%SD)	- (12.92)	55 (5.10)	86.8 (1.63)	90.2 (8.31)	92.59 (8.88)	94.17 (8.00)	95.25 (1.51)

Mixed Infection	Parameter	Control	4	8	16	32	64	128
Candida glabrata Candidiasis - Aerobic vaginitis (V601)	Abs 570 (SD)	1.21 (0.01)	1.20 (0.007)	1.21 (0.008)	0.12 (0.005)	0.11 (0.02)	0.09 (0.015)	0.04 (0.001)
	Abs 630 (SD)	1.15 (0.008)	1.14 (0.005)	1.15 (0.007)	0.07 (0.006)	0.06 (0.01)	0.06 (0.01)	0.03 (0.001)
	Mean CFU (SD)	7.83E+07 (7.21E+06)	3.52E+07 (1.29E+06)	1.11E+07 (2.89E+06)	9.14E+06 (1.59E+06)	4.34E+06 (1.13E+05)	3.56E+06 (6.25E+04)	7.63E+05 (1.25E+04)
	% Eradication (%SD)	- (9.02)	42.69 (12)	71.79 (2.49)	91.96 (10)	94.84 (1.25)	94.80 (46.95)	97.16 (2.28)

Eradication								
**Micafungin**								
**Reference control strain**	**Parameter**	**Concentration (µg/ml)**						
		**Control**	**0.15**	**0.3**	**0.6**	**1**	**2**	**4**
Candida albicans ATCC 10231	Abs 570 (SD)	1.34 (0.02)	1.31 (0.03)	1.35 (0.02)	1.33 (0.02)	1.31 (0.03)	1.23 (0.01)	0.44 (0.14)
	Abs 630 (SD)	1.31 (0.02)	1.28 (0.03)	1.32 (0.02)	1.30 (0.02)	1.28 (0.03)	1.20 (0.01)	0.41 (0.13)
	Mean CFU (SD)	3.33E+08 (1.45E+07)	4.44E+07 (1.26E+07)	4.27E+07 (1.38E+07)	2.85E+07 (2.50E+04)	2.70E+07 (1.28E+06)	1.89E+06 (1.26E+06)	2.38E+05 (1.25E+04)
	% Eradication (%SD)	- (4.35)	86.66 (28.44)	87.19 (32.30)	91.44 (0.09)	91.91 (4.73)	99.43 (66.89)	99.93 (5.26)

**Healthy Microbiota**	**Parameter**	**Concentration (µg/ml)**						
		**Control**	**0.15**	**0.3**	**0.6**	**1**	**2**	**4**
Candida albicans (V130)	Abs 570 (SD)	1.28 (0.01)	1.25 (0.011)	1.22 (0.006)	1.2 (0.009)	1.2 (0.01)	1.19 (0.01)	1.13 (0.01)
	Abs 630 (SD)	1.2 (0.01)	1.1 (0.01)	1.1 (0.007)	1.1 (0.02)	1.1 (0.01)	1.08 (0.01)	1.05 (0.01)
	Mean CFU (SD)	1.46E+08 (4.52E+07)	8.29E+07 (1.51E+07)	4.30E+07 (8.84E+06)	2.44E+07 (1.36E+06)	2.37E+07 (2.78E+06)	1.63E+07 (2.50E+05)	1.44E+07 (2.86E+06)
	% Eradication (%SD)	- (30.89)	43.30 (18.21)	70.57 (20.53)	83.32 (5.59)	83.79 (11.71)	88.85 (1.53)	90.13 (19.83)
Candida albicans (V134)	Abs 570 (SD)	1.23 (0.01)	1.15 (0.008)	1.13 (0.01)	1.1 (0.01)	1 (0.02)	1 (0.01)	1 (0.02)
	Abs 630 (SD)	1.14 (0.01)	1.09 (0.006)	1.1 (0.01)	1.06 (0.01)	1.04 (0.02)	1.03 (0.01)	1.03 (0.02)
	Mean CFU (SD)	2.96E+08 (2.84E+07)	6.37E+07 (1.39E+07)	4.78E+07 (1.11E+06)	2.88E+07 (1.34E+07)	1.47E+07 (1.14E+06)	9.06E+06 (1.38E+05)	4.04E+06 (4.88E+05)
	% Eradication (%SD)	- (9.59)	78.50 (21.81)	83.85 (2.33)	90.27 (46.42)	95.03 (7.72)	96.94 (1.52)	98.64 (12.07)
Candida albicans (V196)	Abs 570 (SD)	1.20 (0.06)	1.14 (0.03)	1.13 (0.03)	1.12 (0.04)	1.08 (0.03)	1.04 (0.03)	1 (0.04)
	Abs 630 (SD)	1.13 (0.06)	1.08 (0.03)	1.06 (0.03)	1.05 (0.04)	1.02 (0.04)	1.01 (0.03)	1.01 (0.04)
	Mean CFU (SD)	1.62E+08	5.62E+07	5.21E+07	2.18E+07	1.42E+07 (3.63E+05)	8.21E+06	4.95E+06

**Healthy Microbiota**	**Parameter**	**Concentration (µg/ml)**						
		**Control**	**0.15**	**0.3**	**0.6**	**1**	**2**	**4**
Candida albicans (V251)	Abs 570 (SD)	1.2 (0.01)	1.19 (0.008)	1.16 (0.01)	1.15 (0.01)	1.1 (0.02)	1 (0.01)	1.07 (0.02)
	Abs 630 (SD)	1.13 (0.01)	1.12 (0.006)	1.11 (0.01)	1.09 (0.01)	1.1 (0.02)	1 (0.01)	1.05 (0.02)
	Mean CFU (SD)	8.34E+07 (5.35E+06)	7.51E+07 (9.88E+05)	4.58E+07 (8.63E+05)	1.60E+07 (3.88E+05)	9.34E+06 (2.11E+06)	7.91E+06 (3.75E+04)	4.95E+06 (5.25E+05)
	% Eradication (%SD)	- (6.41)	10 (1.32)	45.04 (1.88)	80.77 (2.42)	88.80 (22.62)	90.51 (0.47)	94.06 (10.61)
Candida albicans (V448)	Abs 570 (SD)	1.08 (0.02)	0.89 (0.07)	0.8 (0.10)	0.78 (0.20)	0.75 (0.15)	0.59 (0.16)	0.5 (0.04)
	Abs 630 (SD)	1.02 (0.02)	0.86 (0.07)	0.78 (0.10)	0.72 (0.20)	0.75 (0.15)	0.56 (0.16)	0.5 (0.04)
	Mean CFU (SD)	1.35E+08 (1.84E+07)	7.95E+07 (9.75E+05)	5.01E+07 (1.00E+05)	4.66E+07 (1.50E+05)	4.05E+07 (2.38E+05)	9.55E+06 (1.75E+05)	7.29E+06 (1.16E+06)
	% Eradication (%SD)	- (13.70)	40.90 (1.23)	62.79 (0.20)	65.41 (0.32)	69.87 (0.59)	92.90 (1.83)	94.58 (15.95)
Candida albicans (V451)	Abs 570 (SD)	1.13 (0.02)	1.11 (0.02)	1.1 (0.02)	1.08 (0.02)	1 (0.03)	1 (0.03)	0.95 (0.06)
	Abs 630 (SD)	1.09 (0.02)	1.07 (0.02)	1.06 (0.02)	1.03 (0.02)	1 (0.03)	0.99 (0.03)	0.91 (0.06)
	Mean CFU (SD)	2.30E+08 (1.84E+07)	6.30E+07	5.48E+07	4.69E+07	1.73E+07	1.01E+07	5.54E+06 (1.13E+05)

		(2.80E+07)	(1.65E+06)	(1.24E+06)	(1.00E+05)		(1.51E+06)	(1.00E+05)				(3.59E+06)	(1.43E+06)	(2.61E+06)	(1.41E+06)	(1.63E+05)	
	% Eradication (%SD)	- (17.32)	65.27 (2.93)	67.80 (2.37)	86.52 (0.46)	91.24 (2.56)	94.93 (18.42)	96.94 (2.02)		% Eradication (%SD)	- (7.98)	72.59 (5.69)	76.16 (2.60)	79.61 (5.57)	92.48 (8.17)	95.61 (1.61)	97.59 (2.03)
Candida glabrata (V197)	Abs 570 (SD)	1.08 (0.02)	0.75 (0.07)	0.7 (0.10)	0.65 (0.20)	0.6 (0.15)	0.59 (0.16)	0.48 (0.04)	*Candida albicans* (V580)	Abs 570 (SD)	1.21 (0.003)	1.18 (0.01)	1.14 (0.04)	1.14 (0.03)	1.13 (0.19)	1.19 (0.02)	1.16 (0.02)
	Abs 630 (SD)	1.02 (0.02)	0.73 (0.06)	0.67 (0.10)	0.63 (0.20)	0.6 (0.15)	0.56 (0.16)	0.45 (0.04)		Abs 630 (SD)	1.15 (0.002)	1.13 (0.01)	1.09 (0.05)	1.08 (0.03)	1.09 (0.02)	1.14 (0.02)	1.1 (0.02)
	Mean CFU (SD)	1.80E+08 (1.14E+07)	5.43E+07 (3.51E+06)	4.30E+07 (3.63E+05)	1.75E+07 (3.91E+06)	1.30E+07 (8.75E+04)	1.18E+07 (1.75E+05)	8.10E+06 (1.40E+06)		Mean CFU (SD)	1.58E+08 (5.88E+06)	4.79E+07 (6.13E+05)	4.66E+07 (6.38E+05)	4.08E+07 (1.14E+07)	4.37E+07 (5.38E+05)	7.02E+07 (2.62E+07)	4.84E+07 (1.21E+07)
	% Eradication (%SD)	- (6.31)	69.83 (6.47)	76.13 (0.84)	90.30 (22.41)	92.78 (0.67)	93.46 (1.49)	95.50 (17.28)		% Eradication (%SD)	- (3.72)	69.65 (1.28)	70.46 (1.37)	74.16 (27.94)	72.33 (1.23)	55.53 (37.36)	69.37 (24.94)
Candida albicans (V202)	Abs 570 (SD)	1.13 (0.02)	1.09 (0.02)	1.1 (0.02)	1.07 (0.02)	1.09 (0.03)	1.01 (0.03)	1 (0.06)									
	Abs 630 (SD)	1.09 (0.02)	1.07 (0.02)	1.09 (0.02)	1.03 (0.02)	1.05 (0.03)	1.02 (0.03)	1 (0.06)									
	Mean CFU (SD)	2.44E+08 (2.78E+07)	1.28E+08 (4.89E+07)	1.38E+08 (1.65E+07)	1.18E+08 (2.88E+07)	1.23E+08 (2.93E+06)	9.44E+07 (2.34E+07)	7.87E+07 (1.27E+07)									
	% Eradication (%SD)	- (11.39)	47.69 (38.28)	43.38 (11.91)	51.67 (24.41)	49.80 (2.39)	61.30 (24.73)	67.73 (16.11)									
**Intermediate Microbiota**	**Parameter**	**Control**	**0.15**	**0.3**	**0.6**	**1**	**2**	**4**	**Intermediate Microbiota**	**Parameter**	**Control**	**0.15**	**0.3**	**0.6**	**1**	**2**	**4**
Candida albicans - Escherichia coli (V118)	Abs 570 (SD)	1.27 (0.05)	1.25 (0.002)	1.21 (0.02)	1.19 (0.02)	1.09 (0.13)	1.09 (0.007)	1.07 (0.01)	*Candida albicans - Gram positive coccus* (V543)	Abs 570 (SD)	1.27 (0.05)	1.22 (0.002)	1.2 (0.02)	1.19 (0.02)	1.15 (0.13)	1.05 (0.007)	0.98 (0.01)
	Abs 630 (SD)	1.23 (0.02)	1.19 (0.02)	1.18 (0.004)	1.18 (0.03)	1.06 (0.11)	1.06 (0.03)	1.05 (0.01)		Abs 630 (SD)	1.2 (0.02)	1.17 (0.02)	1.16 (0.004)	1.15 (0.03)	1.13 (0.11)	1.02 (0.03)	0.97 (0.01)
	Mean CFU (SD)	2.37E+08 (2.82E+07)	8.27E+07 (1.48E+06)	5.54E+07 (4.00E+05)	2.66E+07 2.75E+05)	1.96E+07 (1.39E+06)	1.90E+07 (2.25E+05)	9.88E+06 (5.75E+05)		Mean CFU (SD)	2.25E+08 (2.58E+07)	9.97E+07 (1.49E+07)	7.28E+07 (9.95E+06)	4.88E+07 (5.23E+06)	2.31E+07 (4.10E+06)	1.41E+07 (1.63E+06)	8.81E+06 (2.38E+05)
	% Eradication (%SD)	- (11.91)	65.14 (1.78)	76.66 (0.72)	88.79 (1.03)	91.73 (7.07)	91.98 (1.18)	95.84 (5.82)		% Eradication (%SD)	- (11.47)	55.65 (14.91)	67.63 (13.68)	78.30 (10.71)	89.74 (17.79)	93.71 (11.50)	96.08 (2.70)
**Candidiasis**	**Parameter**	**Control**	**0.15**	**0.3**	**0.6**	**1**	**2**	**4**	**Candidiasis**	**Parameter**	**Control**	**0.15**	**0.3**	**0.6**	**1**	**2**	**4**
	Abs 570 (SD)	1.1 (0.03)	1.0 (0.03)	1.04 (0.03)	1.0 (0.02)	0.99 (0.01)	0.98 (0.07)	0.95 (0.04)		Abs 570 (SD)	1.36 (0.01)	0.21 (0.001)	0.13 (0.03)	0.12 (0.02)	0.1 (0.01)	0.1 (0.01)	0.09 (0.05)

Candida albicans (V161)	Abs 630 (SD)	1 (0.03)	0.99 (0.03)	0.97 (0.03)	0.97 (0.03)	0.92 (0.02)	0.90 (0.07)	0.89 (0.04)
	Mean CFU (SD)	1.20E+08 (2.33E+07)	1.02E+08 (1.27E+07)	5.71E+07 (1.48E+06)	5.97E+07 (1.35E+06)	2.43E+07 (2.40E+06)	2.05E+07 (2.43E+06)	1.81E+07 (1.00E+05)
	% Eradication (%SD)	- (19.45)	14 (12.37)	52.24 (2.58)	50.08 (2.26)	79.65 (9.86)	82.83 (11.81)	84.90 (0.55)
Candida albicans (V218)	Abs 570 (SD)	1.17 (0.07)	1.13 (0.1)	1.1 (0.05)	1.09 (0.09)	1.05 (0.07)	1 (0.1)	0.88 (0.03)
	Abs 630 (SD)	1.1 (0.06)	1.09 (0.1)	1.07 (0.05)	1.06 (0.08)	1.03 (0.06)	0.99 (0.1)	0.83 (0.03)
	Mean CFU (SD)	1.53E+08 (1.44E+07)	8.62E+07 (5.76E+06)	3.16E+07 (6.00E+05)	2.44E+07 2.60E+06)	1.77E+07 (9.75E+05)	1.01E+07 (1.18E+06)	1.23E+07 (7.50E+04)
	% Eradication (%SD)	- (9.41)	43.76 (6.68)	79.38 (1.89)	84.11 (10.67)	88.43 (5.50)	93.42 (11.66)	91.95 (0.60)
Candida albicans (V252)	Abs 570 (SD)	0.97 (0.16)	0.62 (0.05)	0.63 (0.04)	0.53 (0.04)	0.56 (0.05)	0.47 (0.04)	0.04 (0.001)
	Abs 630 (SD)	0.92 (0.15)	0.60 (0.01)	0.61 (0.04)	0.51 (0.04)	0.52 (0.04)	0.44 (0.02)	0.04 (0.001)
	Mean CFU (SD)	3.67E+08 (8.16E+06)	7.64E+07 (1.39E+06)	8.18E+07 (1.26E+07)	1.37E+07 (1.13E+05)	6.36E+07 (1.74E+06)	3.90E+07 (2.00E+05)	3.49E+07 (4.86E+06)
	% Eradication (%SD)	- (2.22)	79.20 (1.81)	77.72 (15.41)	86.26 (0.81)	82.67 (2.73)	89.37 (0.51)	90.49 (13.92)
Candida albicans (V449)	Abs 570 (SD)	0.97 (0.16)	0.82 (0.05)	0.8 (0.04)	0.79 (0.04)	0.67 (0.05)	0.58 (0.03)	0.4 (0.01)
	Abs 630 (SD)	0.92 (0.15)	0.79 (0.04)	0.76 (0.04)	0.74 (0.04)	0.62 (0.01)	0.51 (0.02)	0.34 (0.01)
	Mean CFU (SD)	1.16E+08 (1.72E+07)	6.41E+07 (1.39E+06)	5.58E+07 (1.11E+06)	5.28E+07 (3.64E+06)	4.41E+07 (1.44E+06)	1.46E+07 (1.83E+06)	1.12E+07 (8.75E+04)
	% Eradication (%SD)	- (14.80)	44.73 (2.16)	51.93 (1.99)	54.49 (6.88)	61.99 (3.25)	87.46 (12.54)	90.36 (0.78)
**Mixed Infection**	**Parameter**	**Control**	**0.15**	**0.3**	**0.6**	**1**	**2**	**4**

Candida albicans (V450)	Abs 630 (SD)	1.28 (0.01)	0.13 (0.001)	0.12 (0.03)	0.12 (0.01)	0.09 (0.01)	0.09 (0.01)	0.07 (0.05)
	Mean CFU (SD)	2.44E+09 (6.30E+07)	7.58E+07 (9.75E+05)	6.08E+07 (1.53E+06)	5.83E+07 (2.13E+05)	2.76E+07 (2.40E+06)	3.50E+07 (2.38E+05)	1.74E+07 (1.74E+07)
	% Eradication (%SD)	- (2.58)	96 (1.28)	97.50 (2.50)	97.60 (0.36)	98.86 (8.69)	98.56 (0.67)	99.28 (9.99)
Candida albicans (V535)	Abs 570 (SD)	1.12 (0.01)	0.85 (0.05)	0.81 (0.04)	0.72 (0.04)	0.71 (0.04)	0.68 (0.04)	0.55 (0.04)
	Abs 630 (SD)	1.07 (0.009)	0.79 (0.04)	0.77 (0.045)	0.69 (0.04)	0.67 (0.04)	0.62 (0.04)	0.51 (0.04)
	Mean CFU (SD)	7.42E+07 (8.31E+06)	1.43E+07 (2.38E+05)	8.99E+06 (1.38E+05)	5.65E+06 (1.00E+05)	2.43E+06 (1.00E+05)	1.68E+06 (5.00E+04)	1.14E+06 (1.13E+05)
	% Eradication (%SD)	- (11.20)	80.78 (1.67)	87 (1.53)	92.38 (1.77)	96 (4.12)	97 (2.99)	98.47 (9.89)
Candida albicans (V540)	Abs 570 (SD)	1.11 (0.06)	0.95 (0.05)	0.83 (0.04)	0.82 (0.04)	0.8 (0.04)	0.75 (0.04)	0.7 (0.04)
	Abs 630 (SD)	1.06 (0.008)	0.86 (0.04)	0.79 (0.045)	0.78 (0.04)	0.72 (0.04)	0.71 (0.04)	0.68 (0.04)
	Mean CFU (SD)	5.24E+07 (3.03E+06)	1.27E+07 (1.76E+06)	1.05E+07 (1.15E+06)	8.34E+06 (1.38E+05)	2.35E+06 (2.75E+05)	1.64E+06 (8.75E+04)	9.50E+05 (1.50E+05)
	% Eradication (%SD)	- (5.78)	75.78 (13.89)	80.05 (11)	84.08 (1.65)	95.51 (11.7)	96.87 (5.34)	98.19 (15.79)
**Mixed Infection**	**Parameter**	**Control**	**0.15**	**0.3**	**0.6**	**1**	**2**	**4**

Candida albicans Candidiasis - Aerobic vaginitis (V415)	Abs 570 (SD)	1.76 (0.02)	1.76 (0.15)	1.68 (0.12)	1.66 (0.06)	1.63 (0.04)	1.69 (0.07)	1.6 (0.01)
	Abs 630 (SD)	1.64 (0.09)	1.64 (0.15)	1.60 (0.13)	1.57 (0.06)	1.52 (0.13)	1.61 (0.07)	1.58 (0.02)
	Mean CFU (SD)	5.95E+07 (5.73E+06)	7.94E+07 (1.39E+07)	5.29E+07 (1.16E+06)	4.89E+07 (4.90E+06)	4.22E+07 (2.33E+06)	4.55E+07 (5.25E+05)	1.24E+07 (3.50E+05)
	% Eradication (%SD)	- (9.62)	- (17.46)	11.03 (2.19)	17.86 (10.03)	29.08 (5.51)	23.45 (1.15)	79.23 (2.83)
Candida glabrata Candidiasis - Aerobic vaginitis (V601)	Abs 570 (SD)	1.21 (0.005)	1.18 (0.02)	1.17 (0.02)	1.12 (0.02)	1.1 (0.01)	0.12 (0.02)	0.05 (0.003)
	Abs 630 (SD)	1.16 (0.008)	1.14 (0.01)	1.14 (0.02)	1.05 (0.01)	1.03 (0.01)	0.09 (0.02)	0.03 (0.005)
	Mean CFU (SD)	1.54E+08 (1.39E+07)	7.56E+07 (1.15E+06)	6.93E+07 (2.53E+07)	3.90E+07 (1.10E+06)	1.87E+078 (1.26E+07)	4.99E+06 (1.38E+05)	3.29+06 4.88E+05)
	% Eradication (%SD)	- (9.02)	50.99 (1.52)	55.05 (36)	74.75 (2.82)	87.90 (67.71)	96.77 (2.75)	97.86 (14.82)
Candida albicans Candidiasis - Aerobic vaginitis (V527)	Abs 570 (SD)	1.51 (0.06)	1.31 (0.06)	1.44 (0.04)	1.21 (0.03)	1.14 (0.04)	1.13 (0.03)	1.06 (0.16)
	Abs 630 (SD)	1.42 (0.09)	1.23 (0.06)	1.36 (0.05)	1.19 (0.03)	1.16 (0.03)	1.15 (0.03)	1.03 (0.17)
	Mean CFU (SD)	1.04E+08 (1.35E+07)	3.61E+07 (1.65E+06)	5.51E+07 (1.55E+06)	2.47E+07 (1.74E+06)	1.93E+07 (4.88E+05)	1.25E+07 (8.75E+04)	8.93E+06 (1.75E+05)
	% Eradication (%SD)	- (12.92)	65 (4.56)	47 (2.81)	76.31 (7.02)	81.55 (2.53)	88.01 (0.69)	91.45 (1.96)

Eradication								
**Flucytosine**								
**Reference control strain**	**Parameter**	**Concentration (µg/ml)**						
		**Control**	**16**	**32**	**64**	**128**	**256**	**512**
Candida albicans ATCC 10231	Abs 570 (SD)	1.3 (0.01)	1.3 (0.05)	1.24 (0.03)	1.22 (0.01)	1.15 (0.01)	1.13 (0.09)	0.95 (0.05)
	Abs 630 (SD)	1.27 (0.01)	1.27 (0.05)	1.22 (0.03)	1.2 (0.02)	1.19 (0.01)	1.1 (0.09)	0.90 (0.06)
	Mean CFU (SD)	3.33E+08 (1.45E+07)	1.30E+08 (1.21E+07)	9.34E+07 (1.37E+07)	7.44E+07 (2.68E+07)	7.00E+07 (2.25E+05)	4.14E+07 (1.33E+06)	9.59E+06 (9.63E+05)
	% Eradication (%SD)	- (4.35)	61.01 (9.28)	71.96 (14.64)	77.67 (36.01)	78.99 (0.32)	87.57 (3.20)	97.12 (10.04)

**Healthy Microbiota**	**Parameter**	**Concentration (µg/ml)**						
		**Control**	**16**	**32**	**64**	**128**	**256**	**512**
Candida albicans (V130)	Abs 570 (SD)	1.45 (0.03)	1.28 (0.004)	1.22 (0.007)	1.21 (0.02)	1.2 (0.01)	1.17 (0.01)	1.18 (0.03)
	Abs 630 (SD)	1.31 (0.03)	1.22 (0.007)	1.2 (0.005)	1.2 (0.022)	1.19 (0.01)	1.16 (0.03)	1.17 (0.03)
	Mean CFU (SD)	1.46E+08 (4.52E+07)	5.50E+07 (2.65E+06)	2.50E+07 (2.66E+06)	2.21E+07 (1.53E+06)	1.90E+07 (8.75E+04)	1.10E+07 (3.99E+06)	1.14E+07 (4.89E+06)
	% Eradication (%SD)	- (30.89)	62.42 (4.82)	82.93 (10.67)	84.90 (6.91)	86.98 (0.46)	92.47 (36.21)	92.19 (42.83)
Candida albicans (V134)	Abs 570 (SD)	1.21 (0.01)	1.16 (0.01)	1.16 (0.01)	1.15 (0.01)	1.13 (0.01)	1.1 (0.005)	1 (0.02)
	Abs 630 (SD)	1.16 (0.009)	1.12 (0.01)	1.11 (0.01)	1.11 (0.01)	1.1 (0.01)	1 (0.008)	1 (0.02)
	Mean CFU (SD)	2.96E+08 (2.84E+07)	6.49E+07 (1.13E+05)	5.62E+07 (1.16E+06)	5.01E+07 (1.51E+06)	1.62E+07 (1.13E+05)	1.34E+07 (1.1E+06)	1.11E+07 (3.38E+05)
	% Eradication (%SD)	- (9.59)	78.10 (0.17)	81.03 (2.07)	83.10 (3.02)	94.52 (0.69)	95.47 (8.29)	96.24 (3.03)
Candida albicans (V196)	Abs 570 (SD)	1.25 (0.05)	1.19 (0.02)	1.17 (0.02)	1.17 (0.03)	1.15 (0.02)	1.09 (0.11)	1.07 (0.05)
	Abs 630 (SD)	1.22 (0.05)	1.2 (0.01)	1.18 (0.02)	1.15 (0.03)	1.1 (0.02)	1.07 (0.10)	1 (0.05)
	Mean CFU (SD)	1.62E+08 (2.80E+07)	7.12E+07 (9.88E+05)	6.15E+07 (1.36E+06)	2.72E+07+ (7.25E+05)	2.10E+07 (3.50E+05)	1.54E+07 (2.75E+05)	9.04E+06 (5.88E+05)

**Healthy Microbiota**	**Parameter**	**Concentration (µg/ml)**						
		**Control**	**16**	**32**	**64**	**128**	**256**	**512**
Candida albicans (V251)	Abs 570 (SD)	1.21 (0.01)	1.2 (0.012)	1.17 (0.01)	1.16 (0.01)	1.14 (0.01)	1.1 (0.005)	1.09 (0.02)
	Abs 630 (SD)	1.16 (0.009)	1.15 (0.01)	1.13 (0.01)	1.11 (0.01)	1.13 (0.01)	1.08 (0.007)	1.05 (0.02)
	Mean CFU (SD)	8.34E+07 (5.35E+06)	8.02E+07 (1.13E+05)	4.77E+07 (2.38E+05)	4.22E+07 (1.15E+06)	3.77E+07 (1.25E+04)	9.39E+06 (1.38E+05)	6.26E+06 (1.38E+05)
	% Eradication (%SD)	- (6.41)	3.85 (0.14)	42.79 (0.50)	49.40 (2.73)	54.78 (0.03)	88.74 (1.46)	92.49 (2.20)
Candida albicans (V448)	Abs 570 (SD)	1.11 (0.01)	0.75 (0.10)	0.75 (0.15)	0.66 (0.13)	0.62 (0.02)	0.59 (0.09)	0.56 (0.15)
	Abs 630 (SD)	1.06 (0.01)	0.72 (0.09)	0.73 (0.14)	0.68 (0.12)	0.61 (0.01)	0.57 (0.09)	0.55 (0.14)
	Mean CFU (SD)	1.35E+08 (1.84E+07)	7.02E+07 (6.34E+06)	6.78E+07 (2.26E+06)	4.83E+07 (2.25E+05)	4.19E+07 (2.76E+06)	2.08E+07 (2.75E+06)	1.53E+07 (1.39E+06)
	% Eradication (%SD)	- (13.70)	47.82 (9.03)	49.64 (3.34)	64.09 (0.47)	68.83 (6.59)	84.52 (13.21)	88.60 (9.05)
Candida albicans (V451)	Abs 570 (SD)	1.19 (0.02)	1.1 (0.03)	1.1 (0.04)	1.1 (0.03)	1.1 (0.03)	1.09 (0.02)	1.06 (0.01)
	Abs 630 (SD)	1.14 (0.02)	1.08 (0.03)	1.06 (0.04)	1.07 (0.03)	1.07 (0.02)	1.05 (0.02)	1.03 (0.01)
	Mean CFU (SD)	2.30E+08 (1.84E+07)	7.57E+07 (1.11E+06)	6.16E+07 (2.36E+06)	5.21E+07 (1.24E+06)	4.95E+07 (2.50E+05)	1.72E+07 (1.41E+06)	7.70E+06 (2.25E+05)

	% Eradication (%SD)	- (17.32)	56 (1.39)	62.04 (2.22)	83.20 (2.67)	87.06 (1.67)	90.5 (1.79)	94.42 (6.50)		% Eradication (%SD)	- (7.98)	67.07 (1.47)	73.20 (3.83)	77.36 (2.38)	78.47 (0.50)	92.52 (8.21)	96.65 (2.92)
Candida glabrata (V197)	Abs 570 (SD)	1.11 (0.01)	0.63 (0.10)	0.55 (0.15)	0.50 (0.13)	0.4 (0.02)	0.39 (0.09)	0.39 (0.14)	*Candida albicans* (V580)	Abs 570 (SD)	1.29 (0.01)	1.23 (0.02)	1.1 (0.009)	1.1 (0.012)	1.1 (0.02)	1 (0.006)	1.1 (0.01)
	Abs 630 (SD)	1.05 (0.01)	0.61 (0.09)	0.53 (0.14)	0.48 (0.12)	0.3 (0.01)	0.27 (0.09)	0.26 (0.14)		Abs 630 (SD)	1.25 (0.01)	1.17 (0.02)	1.09 (0.04)	1.09 (0.004)	1.09 (0.01)	1.07 (0.02)	1.08 (0.01)
	Mean CFU (SD)	1.80E+08 (1.14E+07)	6.69E+07 (1.35E+07)	4.95E+07 (6.25E+04)	2.00E+07 (1.40E+06)	1.42E+07 (8.75E+04)	9.53E+06 (2.25E+05)	5.74E+06 (3.63E+06)		Mean CFU (SD)	1.58E+08 (5.88E+06)	4.26E+07 (4.95E+06)	1.33E+07 (2.75E+05)	1.32E+07 (1.49E+06)	1.42E+07 (2.56E+06)	7.48E+06 (7.50E+04)	9.89E+06 (2.36E+06)
	% Eradication (%SD)	- (6.31)	62.82 (20.22)	72.47 (0.13)	88.91 (7.02)	92.10 (0.62)	94.71 (2.36)	96.81 (6.32)		% Eradication (%SD)	- (3.72)	73.05 (11.63)	91.59 (2.07)	91.66 (11.30)	91.01 (18.06)	95.27 (1)	93.74 (23.89)
Candida albicans (V202)	Abs 570 (SD)	1.2 (0.02)	1.10 (0.03)	1.03 (0.04)	1.04 (0.03)	1.01 (0.03)	1 (0.02)	0.68 (0.01)									
	Abs 630 (SD)	1.15 (0.02)	1.04 (0.03)	1.02 (0.04)	0.98 (0.03)	1 (0.02)	1 (0.02)	0.64 (0.01)									
	Mean CFU (SD)	2.44E+08 (2.78E+07)	4.72E+07 (2.23E+06)	2.07E+07 (2.63E+05)	1.66E+07 (1.24E+05)	1.57E+07 (1.88E+05)	1.11E+07 (1.39E+06)	9.68E+06 (1.35E+06)									
	% Eradication (%SD)	- (11.39)	80.67 (4.72)	91.52 (1.27)	93.21 (0.75)	93.58 (1.20)	95.45 (12.49)	96.04 (13.95)									
**Intermediate Microbiota**	**Parameter**	**Control**	**16**	**32**	**64**	**128**	**256**	**512**	**Intermediate Microbiota**	**Parameter**	**Control**	**16**	**32**	**64**	**128**	**256**	**512**
Candida albicans - Escherichia coli (V118)	Abs 570 (SD)	1.33 (0.04)	1.23 (0.04)	1.17 (0.07)	1.16 (0.1)	0.44 (0.04)	0.43 (0.16)	0.33 (0.5)	*Candida albicans - Gram positive coccus* (V543)	Abs 570 (SD)	1.33 (0.04)	1.23 (0.04)	1.17 (0.07)	1.12 (0.1)	0.14 (0.144	0.10 (0.16)	0.09 (0.5)
	Abs 630 (SD)	1.26 (0.02)	1.17 (0.03)	1.11 (0.04)	1.11 (0.09)	0.42 (0.13)	0.41 (0.13)	0.3 (0.4)		Abs 630 (SD)	1.26 (0.02)	1.17 (0.03)	1.11 (0.04)	1.1 (0.09)	0.08 (0.13)	0.04 (0.13)	0.03 (0.4)
	Mean CFU (SD)	2.37E+08 (2.82E+07)	9.41E+07 (1.15E+06)	5.52E+07 (1.03E+06)	4.90E+07 (2.46E+06)	2.96E+07 (4.89E+06)	2.57E+07 (2.93E+06)	2.10E+07 (1.66E+06)		Mean CFU (SD)	2.25E+08 (2.58E+07)	1.12E+08 (1.46E+07)	7.43E+07 (7.65E+06)	5.01E+07 (9.30E+06)	2.13E+07 (1.34E+06)	1.89E+07 (3.30E+06)	1.22E+07 (8.63E+05)
	% Eradication (%SD)	- (11.91)	60.31 (1.22)	76.72 (1.86)	79.32 (5.01)	87.52 (16.52)	89.17 (11.39)	91.13 (7.90)		% Eradication (%SD)	- (11.47)	50.30 (13.11)	66.95 (10.30)	77.69 (18.55)	90.53 (6.28)	91.61 (17.51)	94.58 (7.08)
**Candidiasis**	**Parameter**	**Control**	**16**	**32**	**64**	**128**	**256**	**512**	**Candidiasis**	**Parameter**	**Control**	**16**	**32**	**64**	**128**	**256**	**512**
Candida albicans (V161)	Abs 570 (SD)	0.85 (0.04)	0.76 (0.04)	0.75 (0.04)	0.73 (0.04)	0.7 (0.04)	0.73 (0.04)	0.40 (0.03)	*Candida albicans* (V450)	Abs 570 (SD)	1.55 (0.1)	1.32 (0.1)	1.34 (0.05)	1.33 (0.04)	1.23 (0.07)	1.16 (0.03)	1.1 (0.07)
	Abs 630 (SD)	0.80 (0.03)	0.74 (0.03)	0.7 (0.04)	0.7 (0.03)	0.7 (0.04)	0.7 (0.04)	0.37 (0.03)		Abs 630 (SD)	1.49 (0.08)	1.3 (0.1)	1.32 (0.1)	1.32 (0.04)	1.21 (0.07)	1.15 (0.04)	1.08 (0.08)

	Mean CFU (SD)	1.20E+08 (2.33E+07)	7.34E+07 (1.08E+07)	6.75E+07 (1.18E+07)	3.99E+07 (1.36E+07)	3.43E+07 (1.39E+06)	4.06E+07 (3.24E+06)	2.10E+07 (2.11E+06)		Mean CFU (SD)	2.44E+09 (6.30E+07)	4.74E+08 (2.77E+07)	6.08E+08 (1.02E+07)	5.52E+08 (2.14E+07)	3.90E+07 (3.74E+06)	2.19E+07 (1.11E+06)	4.05E+07 (1.53E+06)
	% Eradication (%SD)	- (19.45)	38.63 (14.68)	43.57 (17.54)	66.61 (33.98)	71.32 (4.04)	66.03 (7.97)	82.44 (10.06)		% Eradication (%SD)	- (2.58)	80 (5.84)	75.07 (1.68)	77.37 (3.87)	98.40 (9.58)	99.10 (5.08)	98.34 (3.77)
Candida albicans (V218)	Abs 570 (SD)	1.29 (0.01)	1.25 (0.06)	1.21 (0.10)	1.20 (0.09)	1.2 (0.07)	1.19 (0.01)	1.12 (0.01)	*Candida albicans* (V535)	Abs 570 (SD)	1.13 (0.05)	0.96 (0.05)	0.94 (0.04)	0.88 (0.05)	0.8 (0.04)	0.73 (0.04)	0.7 (0.04)
	Abs 630 (SD)	1.16 (0.08)	1.13 (0.05)	1.12 (0.02)	1.1 (0.07)	1.1 (0.02)	1.09 (0.01)	1.1 (0.006)		Abs 630 (SD)	1.09 (0.04)	0.88 (0.048)	0.85 (0.04)	0.84 (0.048)	0.83 (0.04)	0.72 (0.04)	0.7 (0.04)
	Mean CFU (SD)	1.53E+08 (1.44E+07)	9.02E+07 (3.13E+05)	7.26E+07 (1.36E+07)	2.96E+07 (3.88E+05)	2.40E+07 (8.75E+04)	2.13E+07 (2.46E+06)	1.36E+07 (6.25E+05)		Mean CFU (SD)	7.42E+07 (8.31E+06)	2.24E+07 (1.34E+06)	1.63E+07 (2.38E+05)	1.44E+07 (2.88E+05)	1.10E+07 (6.25E+04)	7.24E+06 (1.13E+05)	6.14E+06 (1.13E+05)
	% Eradication (%SD)	- (9.41)	41.15 (0.34)	52.66 (18.74)	80.68 (1.30)	84.36 (0.36)	86.09 (11.55)	91.11 (4.58)		% Eradication (%SD)	- (11.20)	69.86 (5.98)	78.05 (1.46)	80.57 (1.99)	85.16 (0.57)	90.24 (1.55)	91.73 (1.83)
Candida albicans (V252)	Abs 570 (SD)	0.95 (0.18)	0.63 (0.05)	0.65 (0.03)	0.68 (0.06)	0.62 (0.05)	0.6 (0.04)	0.04 (0.002)	*Candida albicans* (V540)	Abs 570 (SD)	1.10 (0.05)	0.88 (0.05)	0.88 (0.04)	0.86 (0.05)	0.8 (0.04)	0.79 (0.04)	0.75 (0.04)
	Abs 630 (SD)	0.91 (0.16)	0.6 (0.02)	0.63 (0.02)	0.66 (0.02)	0.6 (0.02)	0.6 (0.02)	0.04 (0.002)		Abs 630 (SD)	1.05 (0.04)	0.85 (0.048)	0.84 (0.04)	0.84 (0.048)	0.83 (0.04)	0.78 (0.04)	0.73 (0.04)
	Mean CFU (SD)	3.67E+08 (8.16E+06)	1.31E+08 (2.64E+07)	2.15E+08 (3.90E+07)	2.45E+08 (5.02E+07)	5.97E+07 (1.40E+07)	8.78E+06 (1.00E+05)	5.43E+06 (1.50E+05)		Mean CFU (SD)	5.24E+07 (3.03E+06)	1.60E+07 (1.50E+05)	1.24E+07 (1.34E+06)	1.07E+07 (8.75E+04)	8.40E+06 (4.00E+05)	7.41E+06 (2.88E+05)	5.15E+06 (1.00E+05)
	% Eradication (%SD)	- (2.22)	64.41 (20.16)	41.56 (18.17)	33.39 (20.53)	83.74 (23.42)	97.61 (1.13)	98.52 (2.76)		% Eradication (%SD)	- (5.78)	69.50 (0.94)	76.30 (10.78)	79.59 (0.82)	83.96 (4.76)	85.85 (3.88)	90.17 (1.94)
Candida albicans (V449)	Abs 570 (SD)	0.95 (0.18)	0.95 (0.045)	0.95 (0.043)	0.88 (0.046)	0.63 (0.045)	0.58 (0.044)	0.4 (0.002)									
	Abs 630 (SD)	0.91 (0.16)	0.91 (0.02)	0.91 (0.02)	0.84 (0.02)	0.6 (0.02)	0.54 (0.02)	0.4 (0.002)									
	Mean CFU (SD)	1.16E+08 (1.72E+07)	1.53E+08 (1.36E+07)	1.21E+08 (1.40E+07)	8.96E+07 (1.14E+07)	4.75E+07 (2.38E+05)	1.78E+07 (1.39E+05)	1.52E+07 (1.13E+05)									
	% Eradication (%SD)	- (14.81)	- (8.88)	- (11.58)	22.82 (12.67)	59.06 (0.49)	84.63 (7.77)	86.87 (0.73)									
**Mixed Infection**	**Parameter**	**Control**	**16**	**32**	**64**	**128**	**256**	**512**	**Mixed Infection**	**Parameter**	**Control**	**16**	**32**	**64**	**128**	**256**	**512**
Candida albicans	Abs 570 (SD)	1.77 (0.05)	1.62 (0.16)	1.65 (0.05)	1.62 (0.09)	1.5 (0.09)	1.61 (0.10)	1.59 (0.06)	*Candida glabrata*	Abs 570 (SD)	1.33 (0.01)	1.25 (0.02)	1.23 (0.01)	1.21 (0.01)	1.2 (0.02)	1.07 (0.02)	0.13 (0.01)

Candidiasis - Aerobic vaginitis (V415)	Abs 630 (SD)	1.66 (0.16)	1.61 (0.16)	1.63 (0.05)	1.61 (0.10)	1.5 (0.09)	1.6 (0.10)	1.58 (0.05)
	Mean CFU (SD)	5.95E+07 (5.73E+06)	1.59E+07 (1.50E+05)	1.63E+07 (3.46E+06)	1.39E+07 (5.16E+06)	5.61E+06 (2.38E+05)	1.31E+07 (1.89E+06)	7.81E+06 (1.94E+06)
	% Eradication (%SD)	- (9.62)	73.26 (0.94)	72.57 (21.22)	76.60 (37.10)	90.56 (4.23)	77.99 (14.42)	86.86 (24.8)
Candida albicans Candidiasis - Aerobic vaginitis (V527)	Abs 570 (SD)	1.82 (0.08)	1.64 (0.07)	1.59 (0.07)	1.52 (0.11)	1.46 (0.09)	1.51 (0.05)	1.44 (0.05)
	Abs 630 (SD)	1.79 (0.08)	1.6 (0.07)	1.55 (0.07)	1.5 (0.11)	1.42 (0.09)	1.5 (0.06)	1.4 (0.05)
	Mean CFU (SD)	1.04E+08 (1.35E+07)	1.64E+07 (2.53E+06)	1.43E+07 (2.75E+05)	1.29E+07 (1.13E+06)	1.00E+07 (1.53E+06)	1.07E+07 (1.90E+06)	7.49E+06 (4.88E+05)
	% Eradication (%SD)	- (12.92)	84 (15.37)	86 (1.92)	87.67 (8.73)	90.39 (15.21)	89.75 (17.75)	92.82 (6.51)

Candidiasis - Aerobic vaginitis (V601)	Abs 630 (SD)	1.29 (0.05)	1.22 (0.02)	1.21 (0.01)	1.2 (0.01)	1.16 (0.02)	1.01 (0.01)	0.10 (0.008)
	Mean CFU (SD)	1.54E+08 (1.39E+07)	5.97E+07 (1.10E+06)	2.83E+07 (1.08E+06)	2.02E+07 (3.00E+05)	1.93E+07 (2.59E+06)	1.21E+07 (1.18E+06)	2.53E+06 (2.25E+05)
	% Eradication (%SD)	- (9.02)	61.33 (1.84)	81.68 (3.80)	86.92 (1.48)	87.51 (13.43)	92.18 (9.75)	98.36 (8.91)

Summary table with the absorbance values at 570 and 630 nm, CFU, eradication percentage and their respective standard deviation values obtained in Biofilm tests carried out on 21 samples of different medical classifications plus an ATCC control strain, carried out with 3 commercial antifungals of different chemical families (1. Fluconazole, 2. Micafungin, 3. Flucytosine).

Supplementary Table S7. Overall results of sensitivity of planktonic cells of all clinical isolates using fluconazole, voriconazole, posaconazole, caspafungin, anidulafungin, micafungin, flucytosine and amphotericin B.

Isolates	Sample	Planktonic	Fluconazole	Voriconzole	Posaconazole	Caspofungin	Anidulafungin	Micafungin	Flucytosine	Amphotericin B
**HEALTHY MICROBIOTA**										
C. albicans	V130	Color	Sensitive	Sensitive	Resistant	Resistant	Sensitive	Resistant	Resistant	Resistant
		MIC (µg/mL)	4	0.06	-	-	0.015	-	-	-
C. albicans	V134	Color	Sensitive	Resistant	Sensitive	Sensitive	Sensitive	Sensitive	Sensitive	Resistant
		MIC (µg/mL)	1	-	0.03	0.03	0.015	0.03	4	-
C. albicans	V196	Color	Sensitive	Resistant	Resistant	Resistant	Sensitive	Resistant	Resistant	Resistant
		MIC (µg/mL)	4	-	-	-	0.015	-	-	-
C. albicans	V197	Color	Resistant	Sensitive	Sensitive	Sensitive	Resistant	Sensitive	Resistant	Resistant
		MIC (µg/mL)	-	0.06	0.03	0.03	-	0.03	-	-
C. albicans	V202	Color	Sensitive	Sensitive	Sensitive	Resistant	Sensitive	Resistant	Sensitive	Resistant
		MIC (µg/mL)	4	0.06	0.06	-	0.015	-	16	-
C. albicans	V251	Color	Sensitive	Resistant	Resistant	Sensitive	Resistant	Sensitive	Resistant	Resistant
		MIC (µg/mL)	4	-	-	0.03	-	0.25	-	-
C. albicans	V448	Color	Resistant	Sensitive	Sensitive	Sensitive	Sensitive	Sensitive	Resistant	Resistant
		MIC (µg/mL)	-	0.25	0.06	0.06	0.015	0.25	-	-
C. albicans	V451	Color	Sensitive	Sensitive	Sensitive	Sensitive	Sensitive	Resistant	Resistant	Resistant
		MIC (µg/mL)	2	0.12	0.03	0.03	0.015	-	-	-
C. albicans	V580	Color	Resistant	Sensitive	Sensitive	Sensitive	Sensitive	Resistant	Resistant	Resistant
		MIC (µg/mL)	-	0.06	0.06	0.03	0.015	-	-	-
**INTERMEDIATE MICROBIOTA**										
C. albicans-E. coli	V118	Color	Sensitive	Sensitive	Sensitive	Sensitive	Sensitive	Sensitive	Sensitive	Resistant
		MIC (µg/mL)	2	0.12	0.03	0.12	0.015	0.25	16	-
	V543	Color	Sensitive	Sensitive	Sensitive	Sensitive	Sensitive	Sensitive	Resistant	Resistant

C. glabrata- Gram positive coccus		MIC (µg/mL)	8	0.06	0.03	0.03	0.015	0.06	-	-
**CANDIDIASIS**										
C. albicans	V161	Color	Resistant	Resistant	Resistant	Resistant	Sensitive	Resistant	Resistant	Resistant
		MIC (µg/mL)	-	-	-	-	0.015	-	-	-
C. albicans	V218	Color	Sensitive	Sensitive	Sensitive	Sensitive	Sensitive	Sensitive	Sensitive	Resistant
		MIC (µg/mL)	4	0.06	0.03	0.12	0.015	0.25	8	-
C. albicans	V252	Color	Sensitive	Sensitive	Sensitive	Sensitive	Sensitive	Sensitive	Resistant	Resistant
		MIC (µg/mL)	4	0.06	0.03	0.03	0.015	0.12	-	-
C. albicans	V449	Color	Sensitive	Resistant	Resistant	Sensitive	Sensitive	Sensitive	Sensitive	Resistant
		MIC (µg/mL)	8	-	-	0.03	0.015	0.12	16	-
C. albicans	V450	Color	Sensitive	Resistant	Sensitive	Sensitive	Sensitive	Sensitive	Resistant	Resistant
		MIC (µg/mL)	4	-	0.03	0.03	0.015	0.25	-	-
C. albicans	V535	Color	Sensitive	Resistant	Resistant	Sensitive	Sensitive	Resistant	Sensitive	Resistant
		MIC (µg/mL)	4	-	-	0.03	0.015	-	32	-
C. albicans	V540	Color	Sensitive	Sensitive	Resistant	Sensitive	Sensitive	Resistant	Sensitive	Resistant
		MIC (µg/mL)	4	0.06	-	0.03	0.015	-	32	-
**MIXED INFECTION**										
C. albicans C.-AV	V415	Color	Sensitive	Sensitive	Sensitive	Resistant	Sensitive	Sensitive	Sensitive	Resistant
		MIC (µg/mL)	8	0.06	0.03	-	0.015	0.12	16	-
C. albicans C.-AV	V527	Color	Sensitive	Sensitive	Resistant	Sensitive	Sensitive	Sensitive	Sensitive	Resistant
		MIC (µg/mL)	2	0.06	-	0.03	0.015	0.12	16	-
C. glabrata C.- BV	V601	Color	Resistant	Resistant	Sensitive	Sensitive	Resistant	Sensitive	Resistant	Resistant
		MIC (µg/mL)	-	-	0.03	0.03	-	0.03	-	-
